# *ENTREP/FAM189A2* encodes a new ITCH ubiquitin ligase activator that is downregulated in breast cancer

**DOI:** 10.15252/embr.202051182

**Published:** 2021-12-20

**Authors:** Takumi Tsunoda, Miho Riku, Norika Yamada, Hikaru Tsuchiya, Takuya Tomita, Minako Suzuki, Mari Kizuki, Akihito Inoko, Hideaki Ito, Kenta Murotani, Hideki Murakami, Yasushi Saeki, Kenji Kasai

**Affiliations:** ^1^ Department of Pathology Aichi Medical University School of Medicine Nagakute Japan; ^2^ Protein Metabolism Project Tokyo Metropolitan Institute of Medical Science Tokyo Japan; ^3^ Division of Cancer Epidemiology and Prevention Aichi Cancer Center Research Institute Nagoya Japan; ^4^ Biostatistics Center Kurume University Kurume Japan

**Keywords:** breast cancer, CXCR4, ENTREP, FAM189A2, ITCH, Cancer, Membranes & Trafficking, Post-translational Modifications & Proteolysis

## Abstract

The HECT‐type ubiquitin E3 ligases including ITCH regulate many aspects of cellular function through ubiquitinating various substrates. These ligases are known to be allosterically autoinhibited and to require an activator protein to fully achieve the ubiquitination of their substrates. Here we demonstrate that FAM189A2, a downregulated gene in breast cancer, encodes a new type of ITCH activator. FAM189A2 is a transmembrane protein harboring PPxY motifs, and the motifs mediate its association with and ubiquitination by ITCH. FAM189A2 also associates with Epsin and accumulates in early and late endosomes along with ITCH. Intriguingly, FAM189A2 facilitates the association of a chemokine receptor CXCR4 with ITCH and enhances ITCH‐mediated ubiquitination of CXCR4. FAM189A2‐knockout prohibits CXCL12‐induced endocytosis of CXCR4, thereby enhancing the effects of CXCL12 on the chemotaxis and mammosphere formation of breast cancer cells. In comparison to other activators or adaptors known in the previous studies, FAM189A2 is a unique activator for ITCH to desensitize CXCR4 activity, and we here propose that FAM189A2 be renamed as ENdosomal TRansmembrane binding with EPsin (ENTREP).

## Graphical abstract

ENTREP is an activator for HECT‐type ubiquitin E3 ligase ITCH, which mediates ubiquitination and endocytosis of a chemokine receptor CXCR4. ENTREP downregulation attenuates CXCR4 desensitization, thereby promoting breast cancer stemness.

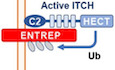

## Introduction

Ubiquitination is the three‐step enzymatic reaction governed by E1 ubiquitin‐activating enzymes, E2 ubiquitin‐conjugating enzymes, and E3 ubiquitin ligases. Among these enzymes, the E3 ubiquitin ligase is key for the specificity of the reaction; E3 recognizes both E2‐bound ubiquitin and a specific substrate and transfers the ubiquitin onto either lysine residues or the amino‐terminus of the substrate. Members of the E3 ubiquitin ligase family are classified into four main types based on their structural similarities and ubiquitination domains: the homologous to E6‐AP carboxyl terminus (HECT) domain‐containing E3 ligases, the really interesting new gene (RING) finger domain‐containing E3 ligases, RING‐in‐between‐RING (RBR) E3 ligases, and the U‐box domain‐containing E3 ligases (Huang *et al*, 2020). In addition, MYCBP2/PHR1 was proposed as a RING‐Cys‐relay (RCR) E3 ligase (Po *et al*, 2010; Pao *et al*, 2018; Mabbitt *et al*, 2020). Among these, HECT‐type E3 ubiquitin ligases have attracted considerable attention because of their critical roles in the maintenance of cellular homeostasis (Bernassola *et al*, 2019).

Upon ligand stimulation, cells transduce the intracellular signals through cell surface receptors to induce the cellular responses. However, the excessive stimulation causes cytotoxicity or impaired cellular function, predisposing to cancer and other diseases. To avoid this, cells attenuate the transmission of intense or prolonged stimulation by means of *desensitization*; endocytosis, and later lysosomal degradation of cell surface receptors (Rajagopal & Shenoy, 2018). Endocytosis starts with a ligand‐induced posttranslational modification, including phosphorylation and ubiquitination, of the receptor. Ubiquitinated receptor is then recognized as a cargo by clathrin‐associated sorting proteins (CLASPs) such as Epsins (EPN1, EPN2), which bridge the cargo to the clathrin coat and actin cytoskeleton required for endocytosis across the plasma membrane (Hawryluk *et al*, 2006; Reider & Wendland, 2011). In particular, the C2‐WW‐HECT (NEDD4‐like) subfamily of the HECT‐type E3 ligases, including ITCH and NEDD4L, exhibits C2 domain‐mediated plasma membrane accumulation (Plant *et al*, 2000; Garrone *et al*, 2009; Wang *et al*, 2010; Lu *et al*, 2011) and participates in receptor ubiquitination and endocytosis of receptors (Rajagopal & Shenoy, 2018; Bernassola *et al*, 2019); ITCH ubiquitinates and enhances endocytosis of a chemokine receptor CXCR4 (Marchese *et al*, 2003; Bhandari *et al*, 2009; Kennedy & Marchese, 2015), the downstream signaling of which facilitates cancer cell migration and stemness (Wang & Knaut, 2014). However, ITCH and many other HECT‐type E3 ligases are allosterically autoinhibited (Riling *et al*, 2015; Attali *et al*, 2017; Chen *et al*, 2017; Sander *et al*, 2017; Zhu *et al*, 2017; Sluimer & Distel, 2018), and the molecular mechanisms underlying the E3 ubiquitin ligase‐mediated endocytosis of receptors are incompletely understood.

We previously identified *FAM189A2/C9orf61/X123* as a downregulated gene in breast cancer, while its function remains unknown (Riku *et al*, 2016). In the present study, we explored the function of the *FAM189A2* gene product; *FAM189A2* encodes a type‐I transmembrane protein primarily expressed at the plasma membrane. FAM189A2 associates with ITCH at the plasma membrane and receives ITCH‐mediated ubiquitination in the PPxY motif‐dependent manner. FAM189A2 also associates with EPN1 and accumulates in early and late endosomes, suggesting the involvement of FAM189A2 in a cargo of the clathrin‐mediated endocytosis. Intriguingly, FAM189A2 enhances CXCR4 binding to the tryptophan‐tryptophan (WW) domain of ITCH as well as ITCH‐mediated ubiquitination of CXCR4. The depletion of *FAM189A2* in breast cancer cells prohibits the CXCL12‐stimulated endocytosis of CXCR4 and, importantly, enhances the cellular function of CXCR4 for the cell migration and stemness. Collectively, these results and the significant correlation of *FAM189A2* downregulation with the poor long‐term prognosis of breast cancer patients illustrate that FAM189A2 is a new activator for ITCH that impacts the biology of cancer cells by regulating the CXCR4 desensitization process. Based on these evidences, we here proposed that *FAM189A2* be renamed ENdosomal TRansmembrane binding with EPsin (*ENTREP*).

## Results

### *FAM189A2/ENTREP* downregulation impacts the prognosis of breast cancer patients

**Figure 1:**
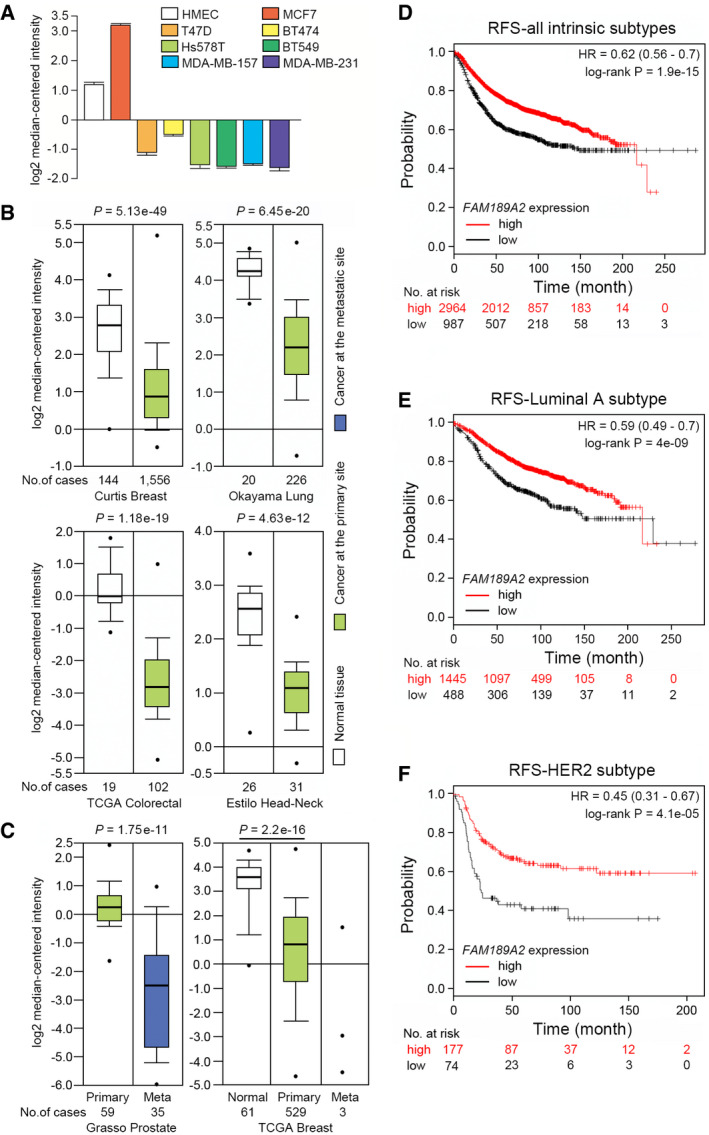
The expression of *FAM189A2/ENTREP* AThe mRNA expression of *FAM189A2*/*ENTREP* in the primary human mammary epithelium HMEC and human breast cancer cell lines. In qRT‐PCR analyses, the expression was normalized to the immortalized normal human mammary epithelium HMEC4*tertshp16*. Relative expression ratios shown as mean + SD from three biological replicates.BRelative expression of *FAM189A2/ENTREP* in the normal (*white*) and cancer (*green*) tissues of breast (Curtis Breast), lung (Okayama Lung), colorectal (TCGA Colorectal), and head and neck (Estilo Head‐Neck). Data were downloaded from the Oncomine database (http://www.oncomine.org). *P*‐values obtained by Student’s *t*‐tests and the number of cases are listed on the top and bottom of figures, respectively. *P* < 0.05 was considered as statistically significant. Box‐whisker plot represents the interquartile range (25^th^ and 75^th^ percentiles) as a box and the median as a line. The maximum and minimum values within 1.5 × interquartile range are shown as whiskers. Outlier data are plotted as dots.CRelative expression of *FAM189A2/ENTREP* in the primary (*green*) and the metastatic sites (*blue*) including lymph node, bone, liver, lung, soft tissues of prostatic cancer (Grasso Prostate) (*left*) and in the normal (*white*), primary (*green*), and metastatic sites (three cases of sentinel lymph node) (*blue*) of breast cancer (TCGA Breast) (*right*). Note that only three cases of the metastatic breast cancer were available in the dataset. Data were downloaded from the Oncomine database (http://www.oncomine.org). *P*‐values obtained by Student’s *t*‐tests and the number of cases are listed on the top and bottom of figures, respectively. *P* < 0.05 was considered as statistically significant. Box‐whisker plot represents the interquartile range (25^th^ and 75^th^ percentiles) as a box and the median as a line. The maximum and minimum values within 1.5 × interquartile range are shown as whiskers. Outlier data are plotted as dots.D–FRelapse‐free survival (RFS) of breast cancer patients. Data were downloaded from the Kaplan–Meier plotter (https://kmplot.com/analysis/). Statistical analysis is described in the Materials and Methods.

**Figure EV1:**
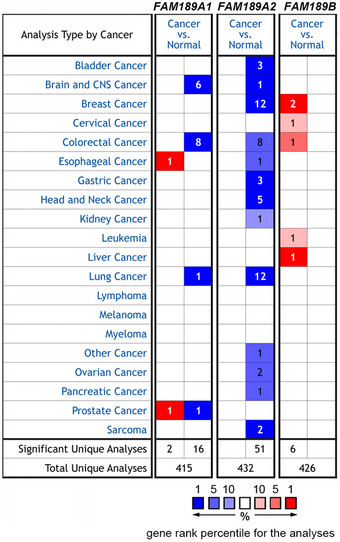
The Oncomine database analyses of *FAM189A1*, *ENTREP/FAM189A2,* and *FAM189B* expression in various types of cancer Number indicates the number of datasets of gene expression analyses.

We previously identified the downregulated genes in breast cancer by comparing publicly available gene expression datasets (Riku *et al*, 2016). By qRT‐PCR (Fig 1A) and the immunoblot analysis (Appendix Fig S1A), one of those genes, *FAM189A2/C9orf61/X123*, was confirmed to be downregulated in several breast cancer cell lines except for MCF‐7, a luminal‐A type cell line. Analysis of the Oncomine database showed that the expression of *FAM189A2*, but neither of its paralogs (*FAM189A1* and *FAM189B*), was downregulated, compared with that in corresponding normal tissues, in various cancers: bladder (Data ref: Leem *et al*, 2008; Lee *et al*, 2010), breast (Curtis *et al*, 2012; Data ref: METABRIC, 2012), lung (Data ref: Kohno, 2011; Okayama *et al*, 2012), colorectal (National Cancer Genome Atlas Network, 2012a, 2012b), gastric (Data ref: Cho *et al*, 2008; Cho *et al*, 2011), and head and neck cancer (Data ref: Singh & Socci, 2008; Estilo *et al*, 2009) (Figs 1B and EV1). Intriguingly, the *FAM189A2* expression level at the metastatic sites of prostatic cancer was lower than that at the primary sites (Grasso *et al*, 2012; Data ref: Tomlins & Chinnaiyan, 2012); this pattern was also observed in breast cancer (National Cancer Genome Atlas Network, 2012b; Data ref: National Cancer Genome Atlas Network, 2012c), although the number of metastatic breast cancers was limited (Fig 1C). To further examine the clinical relevance of *FAM189A2* expression, we analyzed the impact of *FAM189A2* expression on the long‐term prognosis of breast cancer patients using Kaplan–Meier Plotter (https://kmplot.com/analysis/) (Gyorffy *et al*, 2010). We did not find any significant difference in the overall survival (OS) between the groups of breast cancer patients with high and low expression of *FAM189A2*. However, in the relapse‐free survival (RFS) analysis of the combined set of breast cancer patients with all intrinsic subtypes (luminal‐A, luminal‐B, HER2‐enriched, triple‐negative/basal‐like, normal‐like, and claudin‐low) (3,951 at‐risk patients), low expression of *FAM189A2* was significantly correlated with a reduction in the RFS time (HR = 0.62, log‐rank *P* = 1.9e‐15, FDR = 1%); the median RFS times were 148 and 216.66 months for the low‐expression and high‐expression cohorts, respectively (Fig 1D). In addition, in patients with luminal A‐subtype breast cancer (1,933 at‐risk patients), the most common subtype of breast cancer, low expression of *FAM189A2* was associated with a reduced RFS time (HR = 0.59, log‐rank *P* = 4e‐09, FDR = 1%) (Fig 1E). The number of at‐risk patients with HER2 enriched‐subtype breast cancer was lower than that of patients with luminal A‐subtype breast cancer, but the upper quartile of RFS was significantly different: 12.96 months for the low‐*FAM189A2* cohort and 28 months for the high‐*FAM189A2* cohort (HR = 0.45, log‐rank *P* = 4.1e‐05, FDR = 1%) (Fig 1F). However, the outcome of patients with triple‐negative breast cancer was not correlated with *FAM189A2* expression (unpublished observation), indicating that FAM189A2 primarily impacts the biology of the luminal A‐ and HER2‐enriched subtypes of breast cancer.

**Figure 2:**
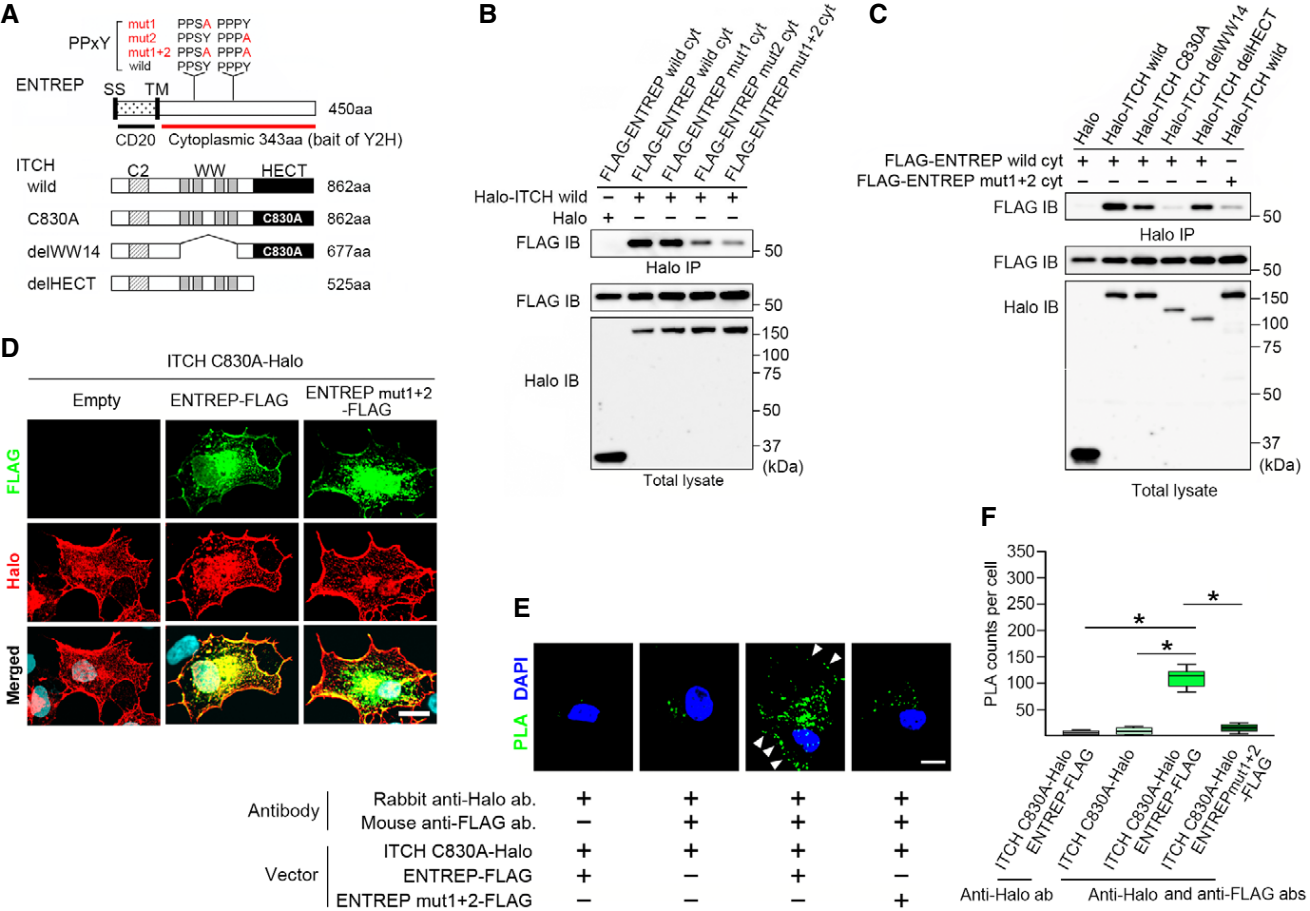
ENTREP associates with ITCH ASchema of the expression vectors of ENTREP and ITCH. SS, signal sequence; CD20, CD20‐homology extracellular domain; TM, transmembrane domain; C2, C2 domain; WW, tryptophan‐tryptophan domain; HECT, the HECT domain; C830A, the catalytically inactive mutation. *Red bar*, cytoplasmic domain of ENTREP and a bait for the yeast two‐hybrid screening.B, CThe immunoprecipitation analysis. Co‐precipitation was impaired by either the WW domain deletion of ITCH or the mutation in the PPxY motifs of ENTREP. Data shown are representative of at least two independent experiments. IP, immunoprecipitation; IB, immunoblot.DThe immunofluorescence staining of Cos7 cells transiently transfected with indicated vectors. *Merged*, merged images of FLAG, Halo and DAPI staining. Scale bar, 20 mm. Data shown are representative of at least three independent experiments.ERepresentative images of the proximity ligation assay (PLA) using Cos7 cells transfected with indicated vectors. PLA signals, granular dots of green color, were presented with DAPI images. *Arrowheads*, the plasma membrane. Scale bar, 10 mm.FThe box‐and‐whisker plot presentation of the PLA. A minimum of 10 cells per condition were counted from two independent experiments. *P*‐values obtained by Student’s *t*‐tests and *P* < 0.05 was considered as statistically significant. *P < 0.05. Box‐whisker plot represents the interquartile range (25^th^ and 75^th^ percentiles) as a box and the median as a line. The maximum and minimum values within 1.5 × interquartile range are shown as whiskers.

To the best of our knowledge, the function of FAM189A2 has not been reported. Therefore, we cloned *FAM189A2* cDNA from MCF‐7 cells and found that this cell line expresses at least two types of *FAM189A2* transcripts. The sequence of one transcript was completely identical to that of *FAM189A2* isoform b (NM_001127608.3), which we proposed in this study to be renamed *ENTREP* based on its subcellular localization and function and registered it under accession number LC496047.1 in the DDBJ/EMBL‐EBI/GenBank database. This *ENTREP/FAM189A2* transcript encoded a type‐I single‐pass transmembrane protein containing 450 aa; structural analyses using the Phobius and CCTOP programs predicted that this protein harbors the signal sequence (SS), a CD20‐homologous extracellular region (55 aa), the transmembrane domain (TM) and the downstream cytoplasmic region (343 aa) at the carboxyl‐terminus (schematic presentation in Fig 2A). The other transcript was found to be the exon 5‐skipping variant of *FAM189A2*, which lacks the entire transmembrane domain (Appendix Fig S1B). When transfected in HEK293T cells, the variant protein was faintly detected, while it increased to the same level of ENTREP/FAM189A2 in the presence of a protease inhibitor MG132 (Appendix Fig S1C). This result indicated protein instability of the exon 5‐skipping variant and prompted us to focus on the cellular function of ENTREP/FAM189A2, but not of the variant.

### ENTREP associates with ITCH and EPN1

To gain insights into the function of ENTREP, we searched for its binding partners by yeast two‐hybrid screening. We used the cytoplasmic region of ENTREP as a bait (Fig 2A) and screened the prey library constructed from cDNAs of human breast cancer cell lines. Many putative binding partners, including HECT‐type E3 ubiquitin ligases (ITCH and NEDD4L) and endocytic adaptor Epsins (EPN1 and EPN2), were identified (Appendix Table S1; the IMEx consortium identifier IM‐29244). Like other HECT E3 ligases, whose C2 domain at the amino‐terminus reportedly participate in their membranous accumulation, ITCH harbors the amino‐terminal C2 domain. EPN1 (and EPN2) is a crucial member of CLASPs for clathrin‐mediated endocytosis of transmembrane receptors (Reider & Wendland, 2011; Messa *et al*, 2014). In addition to these reports, the predicted membrane‐spanning structure of ENTREP prompted us to examine whether ENTREP associates with ITCH and EPN1. We constructed ENTREP and ITCH expression vectors (Fig 2A; Appendix Fig S2 for the schematic summary of the expression vectors used in this study), transfected them into HEK293T cells and subjected the cells to immunoprecipitation analyses. FLAG‐tagged cytoplasmic region of ENTREP (FLAG‐ENTREP wild cyt) co‐precipitated with Halo‐tagged wild‐type ITCH (Halo‐ITCH wild) (Fig 2B). The WW domain of NEDD4‐like subfamily proteins, including ITCH, is reported to interact with proline‐rich PPxY motifs and particular sequences containing phosphorylated serine/threonine residues on substrates (Sudol & Hunter, 2000; Huang *et al*, 2020). ENTREP contains two PPxY motifs (PPSY and PPPY) in the cytoplasmic region (Fig 2A), and as expected, the ENTREP mut1+2 mutant containing PPSA and PPPA sequences co‐precipitated poorly with ITCH (Fig 2B). Consequently, ENTREP co‐precipitated individually with the catalytically inactive mutant (C830A) (Marchese *et al*, 2003) and the HECT domain deletion mutant (delHECT) of ITCH, but not with its tryptophan–tryptophan (WW) domain deletion mutant (delWW14) (Fig 2C), indicating the association between the ENTREP PPxY motifs and the ITCH WW domains. When transiently transfected into Cos7 cells, the carboxyl‐terminal tagged ITCH C830A (ITCH C830A‐Halo), which harbored the intact C2 domain but not E3 activity, was detected at the plasma membrane (Fig 2D; Appendix Fig S2 for the vector information). And the carboxyl‐terminal tagged full‐length ENTREP (ENTREP‐FLAG) and its mut1+2 mutant (ENTREP mut1+2‐FLAG) were expressed at the plasma membrane, where either of these ENTREPs partially overlapped with ITCH C830A‐Halo (Fig 2D). However, co‐transfection of ENTREP‐FLAG, but not of mut1+2 mutant, along with ITCH C830A‐Halo produced many signals of the proximity ligation assay (PLA); these signals were detected in the cell area including at the plasma membrane, indicating the PPxY motif‐dependent association of full‐length ENTREP with ITCH at the plasma membrane (Fig 2E and F for the box‐and‐whisker plot presentation of the PLA).

**Figure 3:**
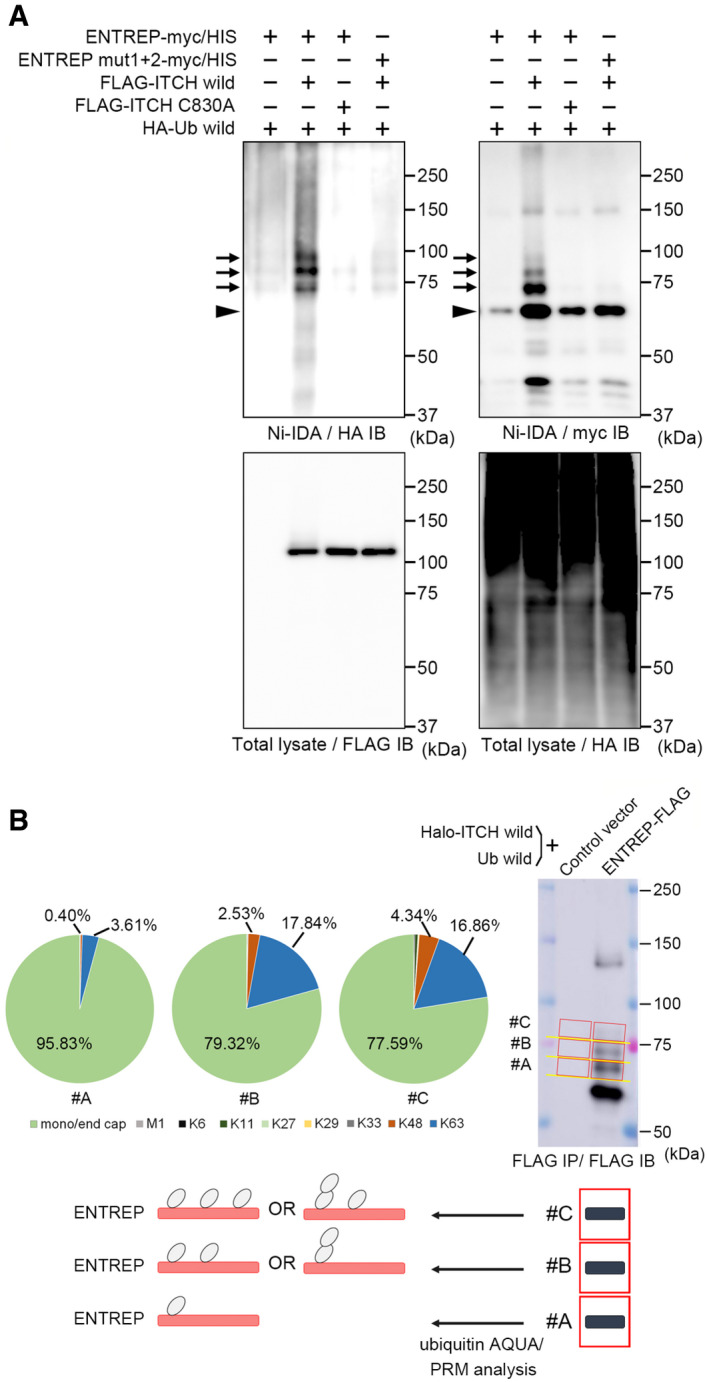
ITCH ubiquitinates ENTREP The nickel‐pull down assay using myc/HIS‐tagged ENTREP along with HA‐tagged ubiquitin. *Arrows* indicate HA‐ubiquitin‐incorporating ENTREP. *Arrowhead* indicates ENTREP without ubiquitination. Data shown are representative of three independent experiments.The ubiquitination of ENTREP. The immunoprecipitated samples using anti‐FLAG antibody under the denaturing condition were separated by the SDS‐PAGE. The gel area corresponding to band #A, #B, and #C of ENTREP‐FLAG were applied to the ubiquitin‐AQUA/PRM analysis. The absolute number of each ubiquitins of ENTREP #A, #B, and #C were calculated by subtraction of those of the corresponding area of the control vector sample (Appendix Table S2 for raw data of the ubiquitin‐AQUA/PRM analysis). Based on the ratios of each ubiquitins to the total ubiquitin of indicated gel area, which are showed as Circular graphs (ENTREP #A, #B, and #C), the number of ENTREP‐linked ubiquitin (Ub) is summarized as schema: #A is modified with one Ub, and about two thirds of #B molecules and half of #C molecules are modified with two and three of single Ubs (multi‐monoubiquitin), respectively. The result was based on three biological replicates.

**Figure EV2:**
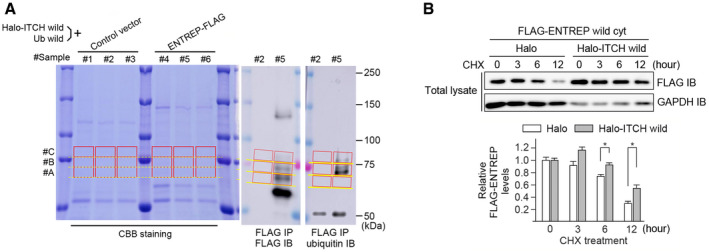
Ubiquitination of ENTREP Sample preparation for the ubiquitin‐AQUA/PRM analysis. The immunoprecipitated samples using anti‐FLAG antibody under the denaturing condition were separated by the SDS‐PAGE and served for the immunoblot analyses and the Coomassie Brilliant Blue (CBB) gel staining. The gel area corresponding to the band #A, #B, and #C of the immunoblot were excised from the CBB‐stained gel and used for ubiquitin‐AQUA/PRM and shotgun MS analyses. The immunoblot image of FLAG IP/FLAG IB is the same with that of Fig 3B. We analyzed samples from three biological replicates (#1‐3 of the control samples and #4‐6 of ENTREP samples). The raw data of the ubiquitin‐AQUA/PRM analysis are listed on Appendix Table S2 and the result of the shotgun MS is in Appendix Fig S3.The cycloheximide chase assay. Twenty‐four hours after transfection, HEK293T cells were incubated with 50 mg/ml cycloheximide for the indicated time periods and served for the immunoblot analyses. The immunoblot analyses were carried out using six independent samples, and their blot bands were semi‐quantified using ImageJ software. The relative FLAG‐ENTREP expression levels were calculated as a ratio of GAPDH‐adjusted FLAG‐ENTREP at each time points and presented as a mean + SD from six biological replicates. *P*‐values obtained by Student’s *t*‐tests and *P* < 0.05 was considered as statistically significant. *P < 0.05. Note that, in the immunoblot analyses, the volume of Halo‐ITCH transfected samples applied was a half of Halo‐transfected samples applied, as indicated by GAPDH.

We next examined whether ITCH ubiquitinates ENTREP. We transiently transfected HEK293T cells with myc/histidine‐tagged full‐length ENTREP vectors (Appendix Fig S2) along with HA‐tagged ubiquitin (UBC) vector and subjected the cells to the nickel‐pull down assay under the denaturing condition. We found that ITCH but not its C830A mutant ubiquitinated ENTREP and that ITCH did not ubiquitinate the mut1+2 mutant of ENTREP (Fig 3A); the immunoblot analyses showed three bands of HA‐ubiquitin‐incorporating ENTREP in ITCH‐transfected cells (Fig 3A, *Arrows*). To examine the ubiquitination status of these, we applied the ubiquitin absolute quantification (AQUA)/parallel reaction monitoring (PRM), a quantitative, high‐resolution mass spectrometry analysis (Tsuchiya *et al*, 2013). We transfected ENTREP‐FLAG or its control vector along with Halo‐ITCH wild as well as ubiquitin without a tag sequence into HEK293T cells. The cells were served to the immunoprecipitation using FLAG‐antibody under the denaturing condition, as our previous reports with slight modifications (Tsuchiya *et al*, 2017; Kaiho‐Soma *et al*, 2021) and analyzed by the immunoblot as well as the Coomassie Brilliant Blue (CBB) gel staining. The gel area corresponding to band #A, #B, and #C of ENTREP‐FLAG were excised from the CBB‐stained gel and used for the ubiquitin‐AQUA/PRM analysis (Figs 3B and EV2A for sample preparation for the ubiquitin‐AQUA/PRM analysis; Appendix Table S2 for the raw data of ubiquitin‐AQUA/PRM analysis). The results revealed that the total amount of ubiquitin of ENTREP #A, B, and C were 36.87, 55.40, and 39.99 fmol, respectively. 95.83, 79.32, and 77.59% of those were found to be monoubiquitin/endo cap (Fig 3B). Considering the band size of #A, #B, and #C, we concluded that ITCH mainly modified ENTREP with a multi‐monoubiquitin and, to a lesser extent, with K63‐linked ubiquitin (schematic presentation in Fig 3B). Shotgun MS analysis of these samples, while the analysis did not cover all of lysine residues of ENTREP, identified ubiquitination of K274, K329 (#A, #B, and #C), and K365 (#C), supporting the modification of ENTREP with a multi‐monoubiquitin (Appendix Fig S3 for the raw data of shotgun MS analysis). We found that co‐transfection of ITCH slightly stabilized ENTREP protein in the cycloheximide chase assay (Fig EV2B), but a role of ENTREP ubiquitination is to be determined in future studies.

**Figure 4:**
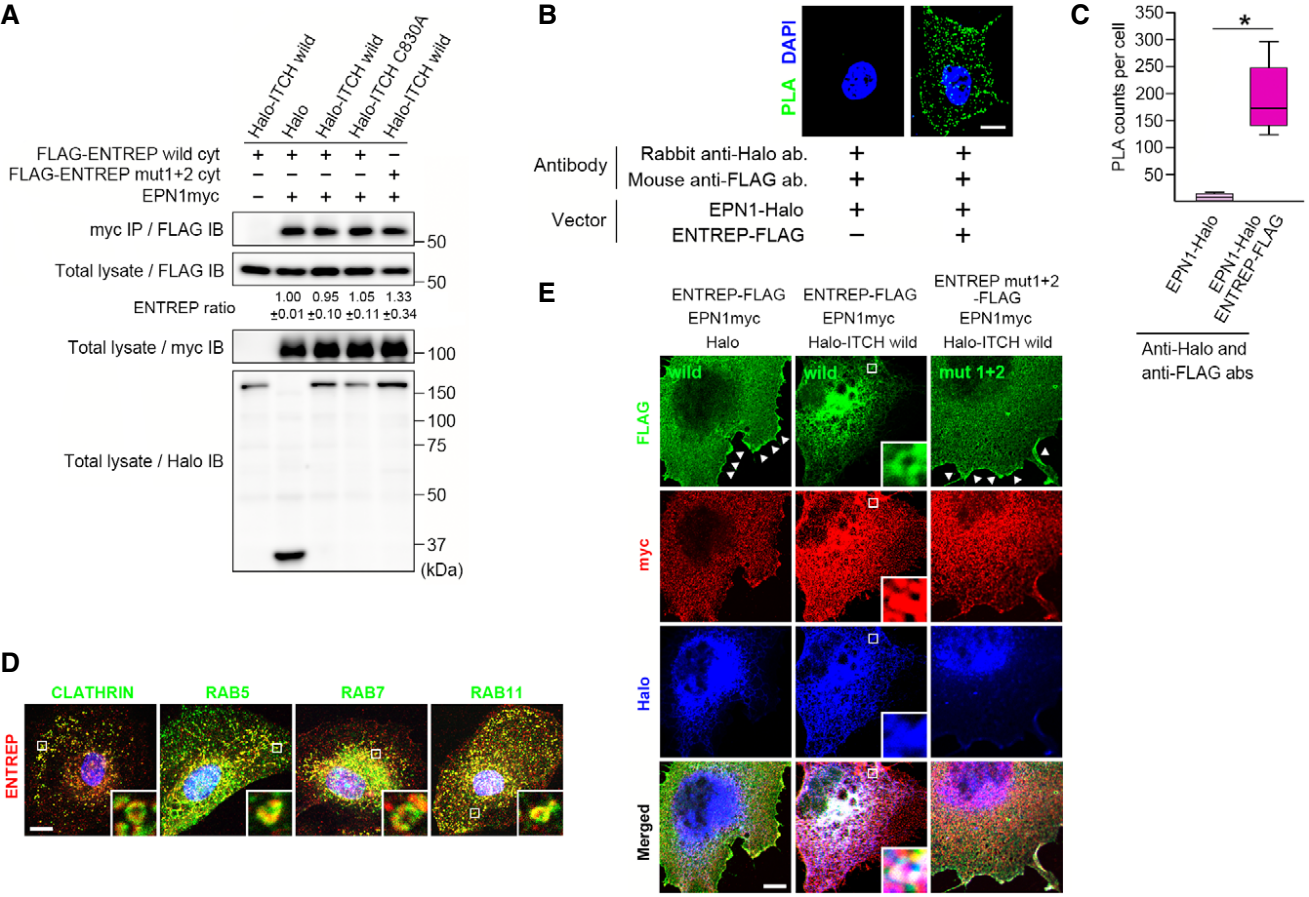
ENTREP associates with EPN1 The immunoprecipitation analysis. The cytoplasmic ENTREP (wild or mut1+2 mutant) co‐precipitated with EPN1myc in the presence of ITCH wild or C830A. The blot bands were semi‐quantified using ImageJ software. ENTREP ratio was calculated as a ratio of ENTREP in the myc‐coprecipitaed sample to that of the total lysate. The ratios shown as mean ± SD from three biological replicates. *P*‐values were obtained by Student’s *t*‐tests and *P* < 0.005 was considered as statistically significant. No significant difference was observed.Representative images of the proximity ligation assay (PLA) using Cos7 cells transfected with indicated vectors. PLA signals, granular dots of green color, were presented with DAPI images. Scale bar, 10 mm.The box‐and‐whisker plot presentation of the PLA. A minimum of 10 cells per condition were counted from two independent experiments. *P*‐values obtained by Student’s *t*‐tests and *P* < 0.05 was considered as statistically significant. *P < 0.05. Box‐whisker plot represents the interquartile range (25^th^ and 75^th^ percentiles) as a box and the median as a line. The maximum and minimum values within 1.5 × interquartile range are shown as whiskers.The expression of endogenous ENTREP in HMEC. ENTREP co‐localized with Clathrin, RAB5, RAB7, and RAB11. *Inserts*, the ring‐shaped structures of the marked areas. The images are representative of at least two independent experiments. Scale bar 10 mm.Co‐localization of ENTREP with ITCH and EPN1. Cos7 cells transfected with indicated vectors were stained with specific antibodies and observed under a laser confocal microscope. *Arrowheads*, full‐length ENTREP (wild or mut1+2 mutant) expression at the plasma membrane. *Inserts*, the high‐magnification images of the marked area indicating the co‐localization of ENTREP‐FLAG, EPN1myc and Halo‐ITCH wild. The images are representative of at least two independent experiments. Scale bar 10 mm.

We next examined whether ENTREP associates with EPN1. The immunoprecipitation analyses revealed the association between EPN1 and the cytoplasmic region of ENTREP (Fig 4A). And the PLA revealed the association between EPN1 and the full length of ENTREP in transfected Cos7 cells (Fig 4B and C for the box‐and‐whisker plot presentation of the PLA). EPN1 (and EPN2) harbors the ubiquitin‐interacting motifs (UIMs) responsible for the association with ubiquitinated proteins (Appendix Fig S2); the UIM of EPN1 reportedly binds K63‐linked polyubiquitin chains and it shows extremely poor affinity for monoubiquitin (Hawryluk *et al*, 2006). As showed in Fig 4A, co‐transfection of neither wild type nor C830A mutant of ITCH changed in the co‐precipitation of ENTREP with EPN1, and the co‐precipitation of the PPxY‐motif mutated ENTREP mut1+2 was not obviously reduced than that of wild‐type ENTREP in the presence of wild‐type ITCH. These evidences indicated that the association of ENTREP with EPN1 was not dependent on the ubiquitination of ENTREP.

### Subcellular localization of ENTREP

Based on the results that ENTREP is a membrane‐spanning protein and associates with EPN1, we speculated that ENTREP is incorporated into endosomes. In primary human mammary epithelial cells (HMECs), endogenous ENTREP was detected faintly at the cell surface and dominantly in tiny ring‐shaped structures in the cytoplasm; these structures were also marked with Clathrin, RAB5 (the early endosome marker), RAB7 (the late endosome marker) and, to a lesser extent, with RAB11 (the marker for endosomal recycling to the cell surface), indicating the incorporation of ENTREP in endosomes (Fig 4D). When co‐transfected with EPN1myc and control Halo vectors into Cos7 cells, the expression of ENTREP‐FLAG was detected at the plasma membrane, where it partially overlapped with EPN1myc (Fig 4E, *arrowheads* in left panel). When co‐transfected with ENP1myc and Halo‐ITCH wild, however, ENTREP‐FLAG was not detected at the plasma membrane but instead observed in the cytoplasm, where it co‐localized with ITCH and EPN1 (Fig 4E, middle panel); this suggested ITCH‐induced endocytosis of ENTREP. Intriguingly, ENTREP mut1+2‐FLAG, which associated with EPN1 but not ITCH (Fig 2B and C, and E, and F for ITCH association; Fig 4A for EPN1 association), was detected at the plasma membrane even when co‐transfected with EPN1myc and Halo‐ITCH wild (Fig 4E, *arrowheads* in right panel). In light of the ubiquitination‐independent ENTREP association of EPN1 (Fig 4A), these evidences suggested that the association with ITCH, but not the ubiquitination by ITCH, enhanced endocytosis of ENTREP into endosomes.

In sum, the transmembrane protein ENTREP is associated with ITCH and EPN1 and endocytosed into endosomes. This report is the first to demonstrate the structure and function of the *FAM189A2* gene product; therefore, we here proposed to rename *FAM189A2 ENTREP* (i.e., ENdosomal TRansmembrane binding with EPsin).

### ENTREP enhances the ubiquitination of CXCR4

**Figure 5:**
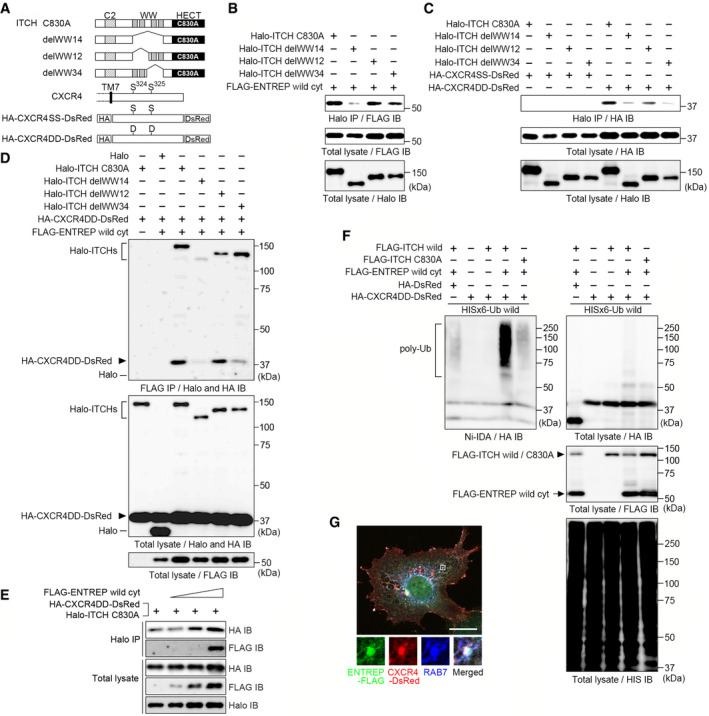
ENTREP enhances the ubiquitination of CXCR4 Schema of the expression vectors of ITCH deletion mutants and the carboxyl‐terminal tail of CXCR4. TM7, the seventh transmembrane domain of CXCR4; HA, hemagglutinin tag; DsRed, DsRed‐momoner (Clontech/TAKARA).The immunoprecipitation analysis. Cytoplasmic ENTREP co‐precipitated with either ITCH C830A, delWW12, or delWW34 but not with delWW14. Data shown are representative of at least two independent experiments.The immunoprecipitation analysis. HA‐CXCR4SS‐DsRed hardly co‐precipitated with either ITCHC830A or delWW mutants. HA‐CXCR4DD‐DsRed co‐precipitated with ITCH C830A and delWW12 but not with delWW14 and delWW34. Data shown are representative of at least two independent experiments.The immunoprecipitation analysis. HA‐CXCR4DD‐DsRed co‐precipitated with ENTREP when co‐transfected with either ITCH C830A or delWW12. Data shown are representative of at least two independent experiments.The immunoprecipitation analysis. The increased transfection of *ENTREP* vector enhanced precipitation of HA‐CXCR4DD‐DsRed with ITCH C830A. Data shown are representative of at least two independent experiments.The nickel‐pull down assay using indicated vectors along with HISx6‐tagged wild‐type ubiquitin vector. HA‐tagged DsRed was used as a control for HA‐CXCR4DD‐DsRed. *Arrowhead*, FLAG‐tagged ITCH (wild or C830A); *arrow*, FLAG‐ENTREP wild cyt. Data shown are representative of at least two independent experiments.Co‐localization of ENTREP and CXCR4 in the endosome. Cos7 cells were transfected with ENTREP‐FLAG, CXCR4‐DsRed, and Halo‐ITCH wild vectors and treated with CXCL12. ENTREP‐FLAG and CXCR4‐DsRed co‐localized in the RAB7‐positive endosome. *Inserts*, the high‐magnification images of the marked area indicating the colocalization of ENTREP‐FLAG, CXCR4‐DsRed, and RAB7. Scale bar 10 mm. The image is representative of at least two independent experiments.

**Figure EV3:**
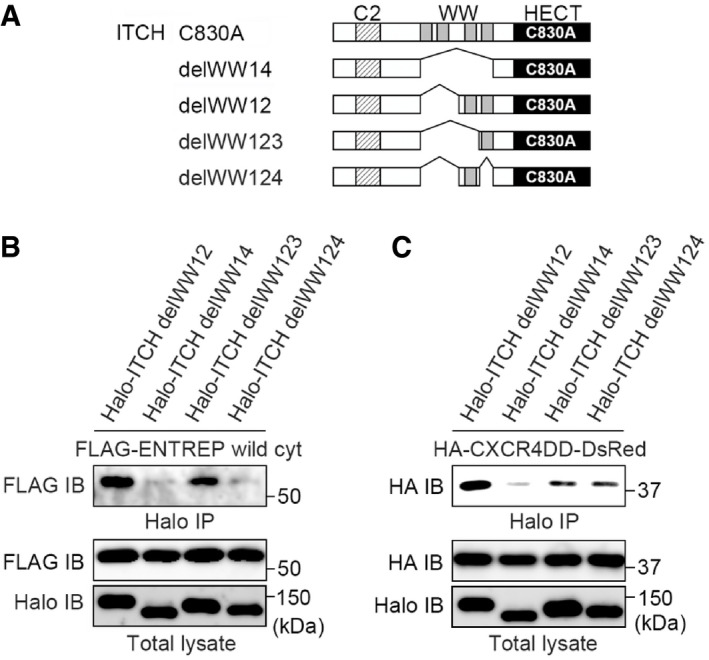
The ITCH WW domain responsible for the association with ENTREP and CXCR4 ASchema of the expression vectors of ITCH deletion mutants.B, CThe immunoprecipitation analysis. FLAG‐ENTREP wild cyt co‐precipitated with Halo‐ITCH delWW123 but not with delWW124, whereas HA‐CXCR4DD‐DsRed co‐precipitated equally with either Halo‐ICTH delWW123 or delWW124. Data shown are representative of at least two independent experiments.

The requirement of the ITCH association for ENTREP endocytosis suggested a possibility that another substrate of ITCH is involved as a cargo protein in ENTREP‐containing endosomes. To date, many proteins were reported to be ubiquitinated by ITCH. Among those, we focused on CXCR4 as a candidate of ITCH substrate in ENTREP‐containing endosomes. Dysregulated activation of CXCR4 has attracted considerable attention in cancer research; CXCR4 expression promotes survival of breast cancer cells and is required for the generation of migrating cancer stem cells (Smith *et al*, 2004; Wang & Knaut, 2014; Mukherjee *et al*, 2016). A meta‐analysis demonstrated the clinically significant correlation between CXCR4 overexpression and poor prognosis of breast cancer patients as well as patients with many other malignancies, including hematological, colorectal, esophageal, head and neck, lung, and gynecologic malignancies (Zhao *et al*, 2015). Importantly, ITCH was reported to regulate the ubiquitination and endocytosis of CXCR4; stimulation by the ligand CXCL12 activates intracellular CXCR4 signaling and subsequently induces the phosphorylation of the Ser324 and Ser325 residues at the carboxyl‐terminal tail of CXCR4 by activating the G‐protein‐coupled receptor kinases (GRKs) and protein kinase C (PKC). CXCR4 does not contain a PPxY motif. Instead, phosphorylation of Ser324/325 was reported to be responsible for the ITCH‐mediated ubiquitination and endocytosis of CXCR4 (Marchese *et al*, 2003; Kennedy & Marchese, 2015). Because the WW domains of ITCH reportedly contribute to its association with CXCR4, we sought to identify the WW domain that is involved in the ENTREP association. We constructed expression vectors for ITCH WW‐deletion mutants (delWW12, with deletion of both WW1 and WW2; delWW34, with deletion of both WW3 and WW4) with the C830A mutation to prevent an indirect interaction *via* ubiquitination (Fig 5A; Appendix Fig S2). In the immunoprecipitation analyses, ENTREP co‐precipitated individually with the ITCH delWW12 and delWW34 mutants, indicating that both the WW3‐WW4 and WW1‐WW2 domains enable the association of ITCH with ENTREP (Fig 5B). And we constructed vectors expressing the HA‐tagged carboxyl‐terminal tail of CXCR4 harboring either wild‐type S324/325 (HA‐CXCR4SS‐DsRed) or the phosphorylation‐mimicking S324/325D mutation (HA‐CXCR4DD‐DsRed) and used them for immunoprecipitation analyses (Fig 5A and C). We hardly detect the association of HA‐CXCR4SS‐DsRed with any ITCH constructs. However, HA‐CXCR4DD‐DsRed co‐precipitated with ITCH C830A and delWW12 but poorly with ITCH delWW14 and delWW34, indicating that, at least in our system, the carboxyl‐terminal tail of CXCR4 harboring the phosphorylation‐mimicking S324/S325D mutation was likely to associate with the WW3 and WW4 domains of ITCH (Fig 5C). We then examined whether ENTREP associates with CXCR4. As shown in Fig 5D, ENTREP did not co‐precipitate with HA‐CXCR4DD‐DsRed in cells co‐transfected with the control Halo vector. However, in the presence of Halo‐ITCH C830A, ENTREP co‐precipitated with HA‐CXCR4DD‐DsRed, indicating that ENTREP associates indirectly with CXCR4 *via* ITCH. Moreover, co‐transfection of ITCH delWW12 but not of either delWW14 or delWW34 contributed to HA‐CXCR4DD‐DsRed precipitation of ENTREP, suggesting that the WW3‐WW4 domains of ITCH mediate the formation of the ENTREP‐ITCH‐CXCR4 ternary complex. To detail the responsible domain for the association, we constructed ITCH delWW123 (deletion of WW1, WW2, and WW3) and delWW124 (deletion of WW1, WW2, and WW4) and used them for immunoprecipitation analyses (Fig EV3A–C). We found that ENTREP co‐precipitated with Halo‐ICTH delWW123 but not with delWW124, whereas HA‐CXCR4DD‐DsRed co‐precipitated equally with delWW123 and delWW124. These evidences suggested the possibility that in the ternary complex ITCH used WW4 domain for the association with ENTREP and WW3 domain for the association with CXCR4.

The WW domain of ITCH was reported to interact intramolecularly with its HECT domain, resulting in the closed conformation and allosteric autoinhibition of ITCH activity (Riling *et al*, 2015; Chen *et al*, 2017; Zhu *et al*, 2017). And the PPxY motif‐containing proteins such as NDFIP1 was found to function as an activator for ITCH (Mund & Pelham, 2009; Riling *et al*, 2015; Zhu *et al*, 2017; Sluimer & Distel, 2018); NDFIP1 binds the WW domain, opens the intramolecular structure, and enhances the catalytic activity of ITCH, by allowing the substrates to access to the HECT domain. Based on the result that ENTREP formed a complex with ITCH and CXCR4, we sought to determine whether ENTREP functions as an ITCH activator for CXCR4 ubiquitination. As co‐transfection of ENTREP increased, so did the precipitation of HA‐CXCR4DD‐DsRed with ITCH C830A, indicating that ENTREP potentiates the CXCR4 binding to ITCH (Fig 5E). In the nickel‐pull down assay under the denaturing condition, ITCH did not appreciably ubiquitinate HA‐CXCR4DD‐DsRed; however, co‐transfection of ENTREP enhanced ITCH‐mediated polyubiquitination of HA‐CXCR4DD‐DsRed (Fig 5F). It was well‐known that ITCH‐mediated ubiquitination is key for the CXCL12‐induced endocytosis of CXCR4. Concordantly, we observed that ENTREP‐FLAG and DsRed‐tagged full‐length CXCR4 were colocalized in RAB7‐positive endosomes in ITCH‐transfected CXCL12‐treated Cos7 cells (Fig 5G). Taken together, our evidences revealed that ENTREP enhances the ITCH association and ubiquitination of CXCR4, indicating that ENTREP functions as an ITCH activator for CXCR4 ubiquitination.

### ENTREP regulates endocytosis and function of CXCR4

**Figure 6:**
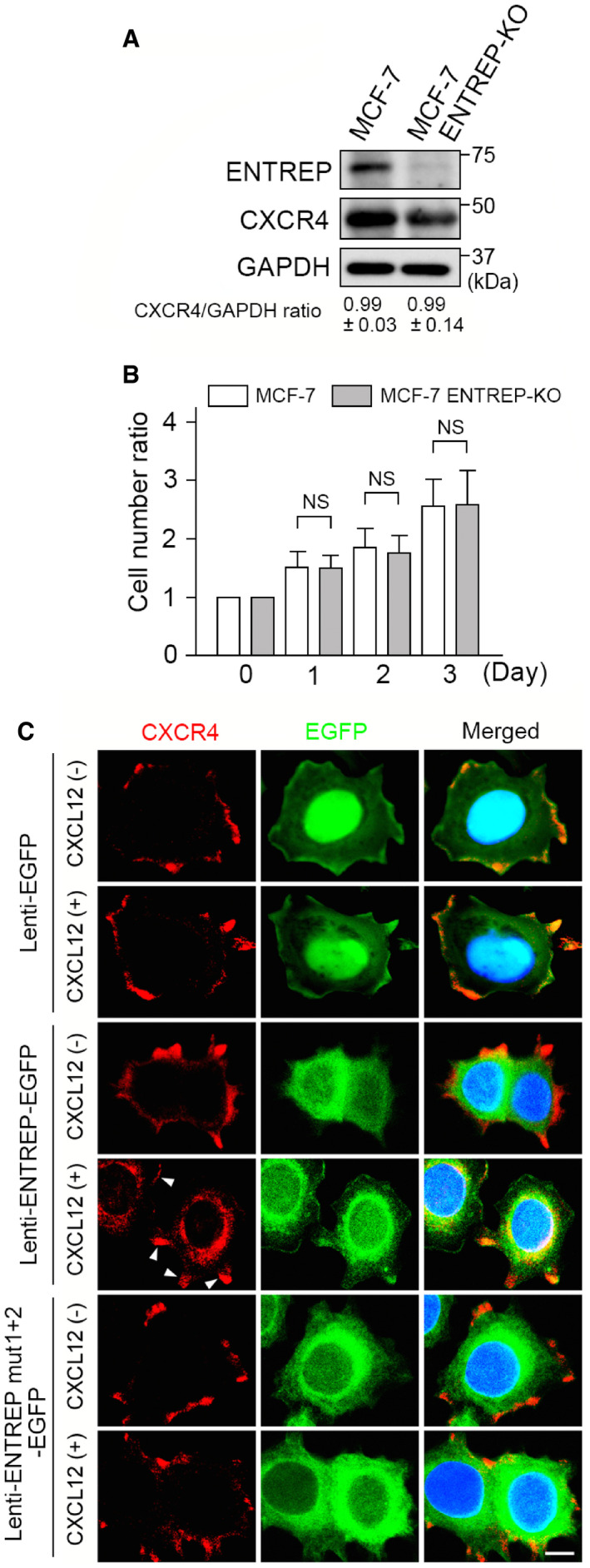
ENTREP participates in CXCL12‐induced endocytosis of CXCR4 The immunoblot analyses of MCF‐7 ENTREP‐KO and parent MCF‐7 cells. Note that the amount of CXCR4 protein was not changed. The immunoblot analyses were carried out using six independent samples, and their blot bands were semi‐quantified using ImageJ software. CXCR4/GAPDH ratio was calculated as a ratio of GAPDH‐adjusted CXCR4 expression. CXCR4/GAPDH ratios shown as mean ± SD from at least four biological replicates. *P*‐values were obtained by Student’s *t*‐tests and *P* < 0.005 was considered as statistically significant. No significant difference was observed.Cell proliferation analysis. Cells were cultured in DMEM supplemented with 10% FBS and counted at day1 to day3. Data are shown as mean ± SD from six biological replicates. *P*‐values were obtained by Student’s *t*‐tests and *P* < 0.005 was considered as statistically significant. NS, not significant.The expression of endogenous CXCR4 in lentivirally transduced MCF‐7 ENTREP‐KO cells. CXCL12 (+) and (−) indicate the presence and absence of CXCL12 treatment. In CXCL12‐treated ENTREP‐EGFP expressing cells, a non‐negligible amount of CXCR4 was observed in the cytoplasm, in which CXCR4 was overlapped with ENTREP‐EGFP. CXCR4 expression on the cell surface still remained (*arrows*). Scale bar 10 mm. The images are representative of at least three independent experiments.

To further examine whether ENTREP participates in the regulation of CXCR4, we established *ENTREP*‐knockout MCF‐7 (MCF‐7 ENTREP‐KO) cells using the CRISPR/CAS9 system. When cultured in DMEM medium supplemented with 10% FBS, CXCR4 expression and the cellular proliferation rate of MCF‐7 ENTREP‐KO cells were not significantly different from those of parent MCF‐7 cells (Fig 6A and B). To examine the localization of endogenous CXCR4, we lentivirally transduced one of ENTREP‐EGFP, ENTREP mut1+2‐EGFP, and control EGFP into MCF‐7 ENTREP‐KO cells and treated them with CXCL12 for 1 h. In either control EGFP‐ or ENTREP mut1+2‐EGFP‐expressing cells, endogenous CXCR4 was detected primarily at the cell surface even with CXCL12 treatment. However, in ENTREP‐EGFP‐expressing cells, a non‐negligible amount of CXCR4 was observed in the cytoplasm when the cells were treated with CXCL12 (Fig 6C), indicating the indispensable role of ENTREP in CXCR4 endocytosis. The immunoblot analyses showed no obvious change in the amount of CXCR4 between any of these transduced cells with or without 1‐h treatment of CXCL12 (Appendix Fig S4), therefore in our experiment system, we were not able to verify whether ENTREP participates in the degradation of CXCR4.

**Figure 7:**
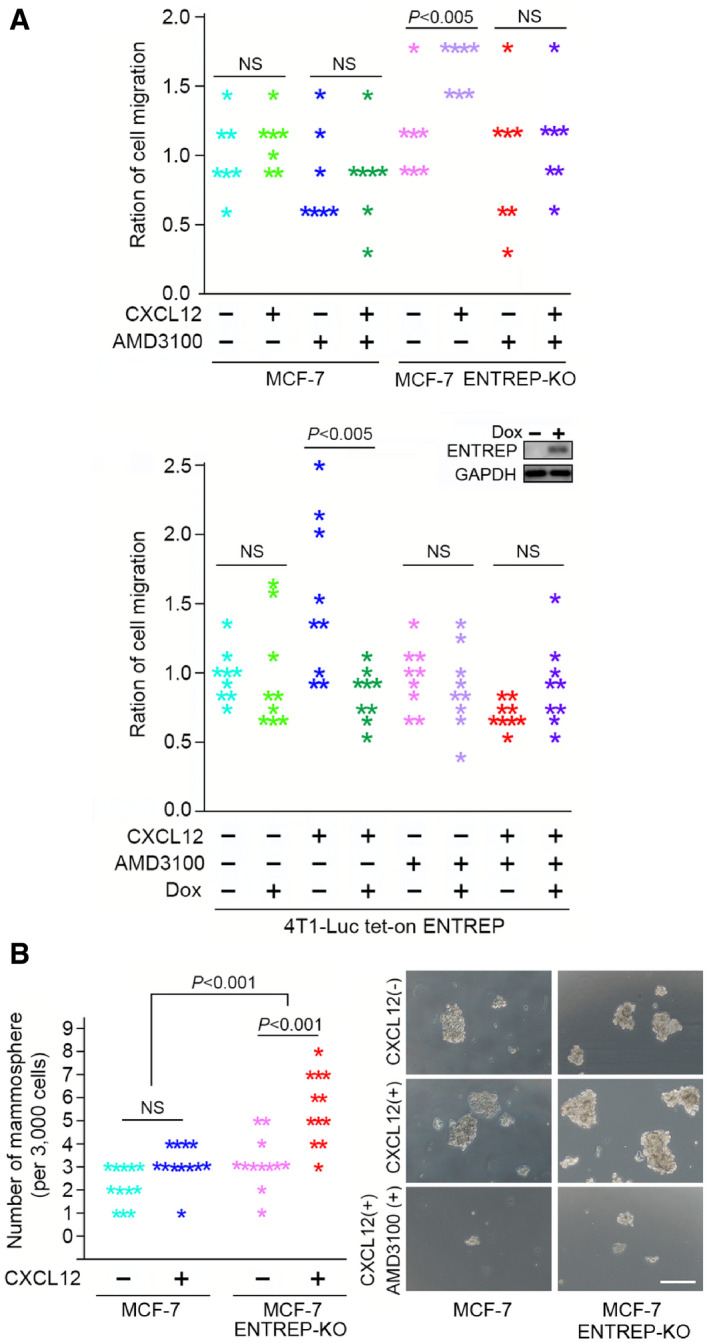
ENTREP fine‐tunes CXCR4 function Dot plot presentation of the chemotaxis analyses using the Boyden chamber. CXCL12 (+) and (−) indicate the presence and absence of CXCL12 as an attractant in the bottom chambers. *ENTREP*‐knockout enhanced the chemotaxis toward CXCL12, which was blocked by pre‐treatment with AMD3100 (*upper* panel). The doxycyclin (DOX)‐induced ENTREP expression suppressed the chemotaxis of mouse 4T1‐Luc cells toward CXCL12 (*lower* panel). Ratio of migrated cells into the bottom chamber against the applied cells on the top chambers was normalized by the results of non‐treated cells (*n* = 7, *upper* panel, and *n* = 9, *lower* panel, from at least two independent experiments, respectively). *P*‐values were obtained by Student’s *t*‐tests and *P* < 0.005 was considered as statistically significant. *Insert*, the immunoblot analysis of the DOX‐induced ENTREP expression in 4T1‐Luc cells. NS, not significant.Dot plot presentation of mammosphere assay (*left* panel) and the images of mammosphere (*right* panel). Each mark in dot plot presentation indicates the number of mammosphere from 3,000 cells *per* well in an ultra‐low attachment plate from two independent experiments. To estimate the effect of CXCL12/CXCR4 pathway, 100 ng/ml of CXCL12 and 12 μM of AMD3100 were applied as indicated. For statistical analysis, variance was assessed using two‐way ANOVA and significance was calculated using Tukey post hoc test correcting for multiple comparison. NS, not significant. Scale bar in the images of mammosphere 100 mm.

CXCL12/CXCR4 signaling induces the migration and stemness of cancer cells (Wang & Knaut, 2014). As we previously reported (Inaguma *et al*, 2015), the chemotaxis assay showed that parental MCF‐7 cells responded only slightly to CXCL12 (Fig 7A, *upper* panel). However, MCF‐7 ENTREP‐KO cells showed enhanced chemotaxis toward CXCL12, and this enhancement was suppressed by pre‐treatment with AMD3100, a specific inhibitor of CXCR4. In addition, doxycycline‐induced ENTREP expression suppressed the chemotaxis of mouse breast cancer 4T1‐Luc cells toward CXCL12. (Fig 7A, *lower* panel). And as previously reported (Ablett *et al*, 2014), parental MCF‐7 cells exhibited only a subtle effect of CXCL12 on increasing sphere formation in the mammosphere assay (Fig 7B, *left* panel; the image of mammosphere in *right* panel). However, MCF‐7 ENTREP‐KO cells showed a significant increase in sphere formation by CXCL12 treatment, and this increase was abolished by treatment with AMD3100 (the image in Fig 7B, *right* panel). Statistical analysis using two‐way ANOVA followed by Tukey multiple comparison test revealed the interaction between CXCL12 treatment and the status of *ENTREP* gene in the mammosphere formation (*P* = 0.0226). These evidences suggest that depletion of *ENTREP* enhances the CXCL12/CXCR4‐mediated chemotaxis and stemness of MCF‐7. Based on these results, we hypothesize that low expression of *ENTREP* potentiates the increase in cancer stem cells (which probably shortens the relapse‐free survival of patients) but not the secondary transformation or chemoresistance of them (which probably shortens the overall survival of patients).

## Discussion

**Figure EV4:**
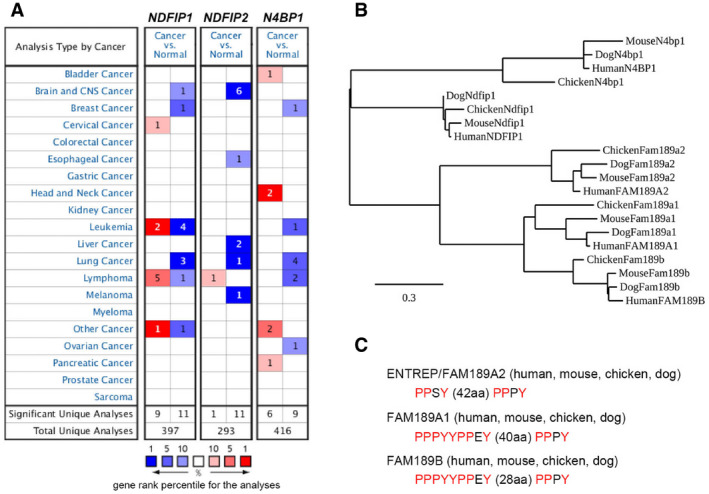
Comparison of *NDFIP1*, *N4BP1,* and *FAM189 family* The Oncomine database analysis of *NDFIP1*, *NDFIP2,* and *N4BP1* expression in various types of cancer. Number indicates the number of datasets of gene expression analyses.Phylogenetic analysis of *FAM189A1*, *ENTREP/FAM189A2*, *FAM189B, NDFIP1,* and *N4BP1*. The coding DNA sequences of these genes were analyzed using Phylogeny.fr software (https://www.phylogeny.fr/). Analyzed sequences were as follows: human *FAM189A1*, NM_015307.1; mouse *Fam189a1*, NM_183087.4; chicken *Fam189a1*, XM_025154007.1; dog *Fam189a1*, XM_025438066.1; human *FAM189A2*, NM_001127608.2; mouse *Fam189a2*, NM_001114174.1; chicken *Fam189a2*, XM_424828.6; dog *Fam189a2*, XM_022421428.1; human *FAM189B*, NM_006589.3; mouse *Fam189b*, NM_001014995.2; chicken *Fam189b*, XM_025143513.1; dog *Fam189b*, XM_005622739.2; human *N4BP1*, NM_153029.4; mouse *N4bp1*, NM_030563.2; chicken *N4bp1*, NM_001030570.1; dog *N4bp1*, XM_022411581.1; human *NDFIP1*, NM_030571.4; mouse *Ndfip1*, NM_001355749.1; chicken *Ndfip1*, XM_414658.5; dog *Ndfip1*, XM_022408883.1.The comparison of PPxY motif. FAM189A1 and FAM189B contain the overlapped PPxY sequences which are separated with PPPY by 40 aa and 28 aa, respectively.

To date, several proteins, such as NDFIP1, NDFIP2, and N4BP1, have been reported to serve as activators or adaptors for HECT‐type E3 ubiquitin ligases (Oberst *et al*, 2007; Mund & Pelham, 2009; Riling *et al*, 2015; Zhu *et al*, 2017; Sluimer & Distel, 2018). The common feature among those proteins is that they contain a motif for binding to the WW domain of the ligase, that is, the PPxY motif in NDFIP1 and NDFIP2 and the noncanonical proline‐rich sequence in N4BP1. Accordingly, the PPxY motifs of ENTREP are responsible for its association with ITCH (Fig 2B and C, and E and F). However, we recognized that ENTREP is quite different from those activators; ENTREP has a single transmembrane domain and is primarily localized at the plasma membrane (Fig 2D), whereas NDFIP1 and NDFIP2 contain three transmembrane domains and are localized in the Golgi apparatus (Harvey *et al*, 2002), and N4BP1 does not have a transmembrane domain and accumulates in the nucleus (Sharma *et al*, 2010). The differences between these activator proteins in their structure and subcellular localization may indicate their distinct roles under physiological and pathological conditions. Indeed, downregulation of *ENTREP* is seen in various types of cancer, whereas *NDFIP1*, *NDFIP2,* and *N4BP1* are infrequently downregulated (Fig EV4A).

**Figure 8:**
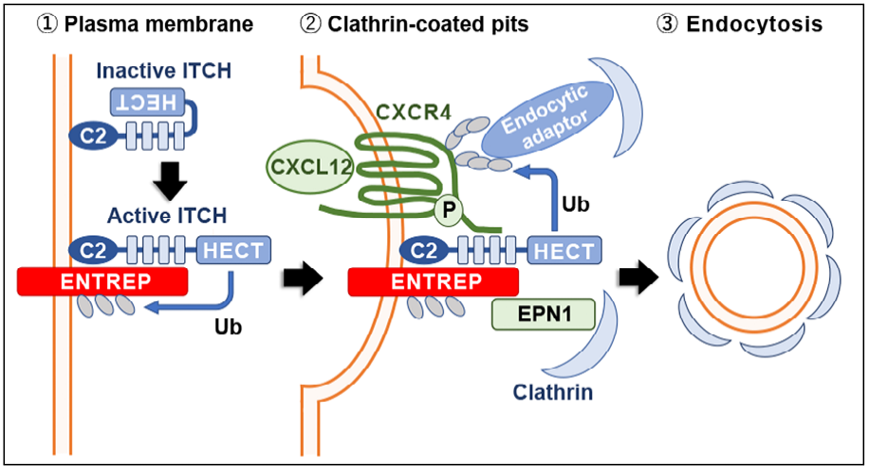
Schematic presentation of the proposed function of ENTREP Plasma membrane‐localized ENTREP associates with ITCH by binding to its WW domain. ITCH modifies ENTREP mainly through the attachment of multi‐monoubiquitin. In addition, ENTREP enhances the ITCH association and polyubiquitination of ligand‐stimulated CXCR4, which leads to the attachment of endocytic adaptors. In collaboration with ENTREP‐associated EPN1 and CXCR4‐attached endocytic adaptors, the ENTREP‐ITCH‐CXCR4 ternary complex is efficiently endocytosed in clathrin‐coated endosomes, leading to desensitization of CXCR4.

Public databases list *FAM189A1* and *FAM189B* as relatives of *ENTREP/FAM189A2*. However, the phylogenetic analysis indicated that *ENTREP/FAM189A2* is not so close to *FAM189A1* or *FAM189B* as *FAM189A1* and *FAM189B* are close to each other. (Fig EV4B). Regarding the PPxY motif in FAM189 family members, ENTREP/FAM189A2 in humans, mice, chickens, and dogs contains conserved PPSY and PPPY motifs, which are separated by 42 aa (Fig EV4C). FAM189A1 and FAM189B in those species contain conserved PPxY motifs, but those motifs are different from those in ENTREP/FAM189A2; FAM189A1 and FAM189B harbor overlapping PPxY sequences (PPPYYPPEY), which are separated from the other PPPY motif by 40 aa and 28 aa, respectively (Fig EV4C). We have not examined whether FAM189A1 and FAM189B are associated with ITCH. However, considering that *FAM189A1* and *FAM189B*, as well as *NDFIP1*, *NDFIP2,* and *N4BP1*, are infrequently downregulated in cancers (Figs EV1 and EV4A), we concluded that *ENTREP/FAM189A2* encodes a unique member of a new class of ITCH activator proteins that are downregulated in various cancers, such as breast cancer. And based on above results and informations, we propose the function of ENTREP in the desensitization of CXCR4 as shown in Fig 8: *via* this process, ENTREP functions as an ITCH activator to *entrap* CXCR4 in endosomes.

ITCH has been reported to ubiquitinate various molecules, and ENTREP may be involved in ITCH‐mediated ubiquitination of other substrates in addition to CXCR4. To date, the consensus sequences exhibiting the binding affinity with the WW domain are grouped as follows: the PPxY or LPxY motifs, the PPLP motif, the proline‐rich sequence with arginine residues (PR motif), the phosphorylated serine/threonine‐proline sequence (p(S/T)P motif) and the PPPPP sequence (Huang *et al*, 2020). For instance, ERBB4/HER4 is a PPxY motif‐containing ITCH substrate (Omerovic *et al*, 2007); transgenic mice expressing the JM‐a/CYT‐1 isoform of the erbb4 gene develop mammary tumors (Wali *et al*, 2014), and sustained expression of ERBB4 activates the MAPK ERK1/2 pathway in response to its ligand heregulin (Vidal *et al*, 2005). In addition, The LPxY motif‐containing tumor suppressor WW domain‐containing oxidoreductase (WWOX) is also an ITCH substrate; depletion of *WWOX* induces anchorage‐independent proliferation of MCF‐7 cells, and knockout mice of this gene develop mammary tumors (Abdeen *et al*, 2011). ITCH induces K63‐linked ubiquitination of WWOX; therefore, ITCH‐mediated ubiquitination performs a tumor‐suppressive role *via* impaired degradation of WWOX (Abu‐Odeh *et al*, 2014). It may be possible that ENTREP participates in the ubiquitination of ITCH substrates such as ERBB4 and WWOX and impacts on the breast cancer biology.

Our yeast two‐hybrid screening (Appendix Table S1; the IMEx consortium identifier IM‐29244) and subsequent immunoprecipitation analyses (unpublished observation) revealed the association of ENTREP with NEDD4L, another member of the HECT‐type E3 ligase family. The WW domains of NEDD4L recognize the PPxY motif. Intriguingly, NEDD4L has been reported to induce the ubiquitination and degradation of the epidermal growth factor receptor (EGFR)‐associated protein ACK1 (Chan *et al*, 2009). ACK1 normally protects EGFR from ubiquitination and endocytosis induced by the RING‐type E3 ubiquitin ligase CBL. Upon EGF stimulation, NEDD4L induces the ubiquitination and degradation of ACK1, leading to CBL‐mediated ubiquitination and endocytosis of EGFR. Considering that ENTREP may function as an activator for NEDD4L, it may be possible that *ENTREP* downregulation impairs the ubiquitination and degradation of ACK1, leading to sustained expression of EGFR at the plasma membrane and the elevated baseline level of downstream signaling.

In conclusion, *ENTREP* encodes an activator protein that regulate ITCH‐mediated CXCR4 ubiquitination and possibly other processes. Because low expression of *ENTREP* significantly impacts the long‐term prognosis of breast cancer patients, the target substrates regulated by the ENTREP‐E3 ligase system as well as the molecular mechanism underlying the downregulation of *ENTREP* would be crucial for further understanding of the biology of breast cancer.

## Materials and Methods

### Cells and quantitative RT‐PCR (qRT‐PCR)

Human breast cancer cell lines were obtained from the American Type Culture Collection and Japanese Collection of Research Bioresources Cell Bank. Intrinsic subtypes of breast cancer cell lines are as follows: MCF‐7, T47D (luminal‐A); BT474 (luminal‐B); and Hs578T, BT549, MDA‐MB‐157, MDA‐MB‐231 (triple‐negative/basal‐like). Primary human mammary epithelial cells (HMECs) were purchased from LONZA. Immortalized normal human mammary epithelial (HMEC4*htertshp16*) cells were a kind gift from Dr. Tohru Kiyono (National Cancer Center Research Institute, Japan) and Dr. Denis Galloway (Fred Hutchinson Cancer Research Center). *ENTREP*‐knockout MCF‐7 cells were generated using the Guide‐it CRISPR‐CAS9 system (TAKARA/Clontech); in brief, the oligonucleotides 5′‐ccgggatttatcctaggctgccaa‐3′ and 5′‐aaacttggcagcctaggataaatc‐3′ were annealed and used as a guide DNA. The linearized pGuide‐it‐tdTomato vector was ligated with the guide DNA to construct the knockdown plasmid. MCF‐7 cells were transiently transfected with the knockdown plasmid. tdTomato‐positive cells were selected using a cell sorter (FACSAria, BD Biosciences) and were cloned *via* the single‐cell cloning method. The established clone, MCF‐7 ENTREP‐KO, was evaluated by genomic DNA sequencing and immunoblot analysis. The stable cell line for doxycycline‐induced expression of ENTREP was established from the mouse breast cancer cell line 4T1‐Luc using the Tet‐on system (TAKARA/Clontech) according to the manufacturer’s protocol. Short tandem repeat (STR) analysis was performed using the GenePrint 10 system (Promega) for authentication of human cell lines. qRT‐PCR was performed using the StepOnePlus real‐time PCR system (Applied Biosystems) and probes for TaqMan Gene Expression Assays (Applied Biosystems) according to the manufacturer's protocol.

### cDNAs, plasmids, lentivirus, and transfection

*ENTREP* cDNA was amplified from MCF‐7 cells using high‐fidelity PrimeSTAR GXL DNA polymerase (TAKARA) and cloned into the pBluescript II plasmid (Agilent). The following PCR primers were used for amplification: forward primer, 5′‐ccggaattcggtcgccaccatgatactcctggtaaacctctttgtg‐3′; reverse primer, 5′‐acgcgtcgactcacaggacagtctctcggatgac‐3′. The sequence was identical to that of *FAM189A2* isoform b (NM_001127608.3) and was registered as *ENTREP* under accession number LC496047.1 in the DDBJ/EMBL‐EBI/GenBank database. The pRK5‐HA‐Ubiquitin plasmids were kind gifts from Dr. Ted Dawson (Johns Hopkins University) through Addgene. The plasmid vectors encoding human ENTREP, ITCH, EPN1, and CXCR4 were constructed from pcDNA3.1‐myc/HIS (Invitrogen), pCMVTNT, pFN21A HaloTag CMV Flexi (Promega), pEGFP, and pDsRed‐monomer vectors (Clontech). The expression vectors were schematically presented in Appendix Fig S2. The lentiviral expression vector pCSII‐CMV‐MCS‐IRES2‐Bsd, as well as pCAG‐HIVgp and pCAG‐VSV‐G‐RSV‐REV, was a kind gift from Dr. Hiroyuki Miyoshi (Riken BioResource Research Center, Japan). Transient transfection of plasmids was carried out using Lipofectamine PLUS reagent (Invitrogen) or Viafect (Promega) according to the manufacturer’s protocol.

### Yeast two‐hybrid screening

Yeast two‐hybrid screening was performed by Hybrigenics Services (Paris, France) (http://www.hybrigenics‐services.com). The coding sequence containing amino acids (aa) 104‐450 of ENTREP was cloned into either the pB27 vector as a LexA C‐terminal fusion or the pB66 vector as a GAL4 C‐terminal fusion. These constructs were sequence verified and used as baits to screen the prey library derived from cDNAs of human breast cancer cell lines. In total, 69.2 million (pB27; LexA‐ENTREP) and 65.3 million (pB66; GAL4‐ENTREP) interactions were examined, and 6 and 335 clones were processed, respectively. The screening results are presented in Appendix Table S1. The protein interactions from the screening have been submitted to the IMEx (http://www.imexconsortium.org) consortium through IntAct (Orchard *et al*, 2014) and assigned the identifier IM‐29244.

### Immunoprecipitation, nickel‐pull down assay, and immunoblot analysis

For the immunoprecipitation, transiently transfected HEK293T cells were lysed in the lysis buffer (20 mM Tris pH 7.5, 150 mM NaCl, 1 mM EDTA, 1% Triton X‐100, 0.5% sodium deoxycholate) containing Complete mini protease inhibitor (Roche). Cell extracts were incubated with indicated antibodies and Dynabeads Protein G (ThermoFisher), washed twice in the lysis buffer and then five times in the wash buffer (50 mM Tris pH 7.5, 150 mM NaCl, 1 mM EDTA, 0.05% Tween‐20) and eluted with SDS sample buffer. For the ubiquitin‐AQUA/PRM analysis, ENTREP‐FLAG was immunoprecipitated under the denaturing condition, as previously reported with slight modifications (Tsuchiya *et al*, 2017; Kaiho‐Soma *et al*, 2021). Briefly, HEK293T cells were transfected with indicated vectors, treated with MG132 and lysed in 1% SDS‐containing buffer (20 mM Tris pH 7.5, 150 mM NaCl, 1 mM EDTA, 1% SDS, 10 mM N‐ethylmaleimide and Complete mini protease inhibitor). After sonication, the lysates were diluted 10‐fold in Triton X‐100‐containing lysis buffer (20 mM Tris pH 7.5, 150 mM NaCl, 1 mM EDTA, 1% Triton X‐100, 10 mM N‐ethylmaleimide, and Complete mini protease inhibitor) and chilled on ice for 30 min. After centrifugation, the supernatants were served for the immunoprecipitation using FLAG‐antibody and elution with SDS sample buffer. The nickel‐pull down assay was conducted under the denatured condition. Twenty‐four hours after transfection with HISx6 tag‐expression vector, HEK293T cells were treated with MG132 and then lysed in the guanidine buffer (6 M guanidine‐HCl, 20 mM phosphate buffer pH 8, 10 mM imidazole), sonicated and incubated with nickel iminodiacetic acid (Ni‐IDA) resin (His60 Ni magnetic beads, TaKaRa) overnight. The resins were then washed eight times in the urea buffer (8 M urea, 20 mM phosphate buffer pH 6.8, 10 mM imidazole) and eluted in the elution buffer (5 M urea, 20 mM Tris buffer pH 6.8, 500 mM imidazole). For the immunoblot analysis, the protein samples were separated in SDS‐PAGE, transferred onto Immunobilon‐P PVDF membrane (Merck) and incubated with indicated antibodies, followed by the HRP‐signal detection using Clarity Max Western ECL substrate (Bio‐Rad).

### Mass‐spectrometric analyses

Mass spectrometry (MS)–based quantification of ubiquitin chains (ubiquitin‐AQUA/PRM) was performed essentially as previously described (Tsuchiya *et al*, 2013, 2017; Kaiho‐Soma *et al*, 2021). Gel regions corresponding to ubiquitinated ENTREP were excised and extensively washed subsequently in 50 mM ammonium bicarbonate (AMBC)/30% acetonitrile (ACN) and 50 mM AMBC/50% ACN. In‐gel trypsin digestion was performed by incubation at 37°C for 15 h with 20 ng/μl Trypsin Gold (Promega) in 50 mM AMBC, 5% ACN, pH 8.0. After trypsin digestion, a mixture of AQUA peptides (25 fmol/injection) was added to the extracted peptides, and the concentrated peptides were diluted with 20 μl of 0.1% TFA containing 0.05% H_2_O_2_, followed by incubation at 4°C for 4 h. For liquid chromatography–tandem mass spectrometry (LC‐MS/MS) analysis, Easy nLC 1200 (Thermo Fisher Scientific) was connected online to Orbitrap Fusion LUMOS (Thermo Fisher Scientific) with a nanoelectrospray ion source (Thermo Fisher Scientific). For targeted acquisition of MS/MS spectra (parallel reaction monitoring) for ubiquitin chain‐derived signature peptides, the Orbitrap Fusion LUMOS instrument was operated in targeted MS/MS mode by Xcalibur software, and peptides were separated using a 55‐min gradient (solvent A, 0.1% FA; solvent B, 80% ACN/0.1% FA) on a C18 analytical column (IonOpticks, Aurora Series Emitter Column, AUR2‐25075C18A 25 cm × 75 μm 1.6 μm FSC C18 with nanoZero fitting). The peptides were fragmented by higher‐energy collisional dissociation (HCD) with a normalized collision energy of 28, and fragment ions were detected by Orbitrap. Data were processed using the PinPoint software 1.3 (Thermo Fisher Scientific), and peptide abundance was calculated based on the integrated area under the curve (AUC) of the selected fragment ions. Means and SD were calculated from three biological replicates. Raw data of the ubiquitin‐AQUA/PRM analysis are listed in Appendix Table S2. Shotgun MS analysis was performed using the same instrumental setting, as previously reported (Kaiho‐Soma *et al*, 2021). The peptides were separated using a 40‐min gradient (solvent A, 0.1% FA; solvent B, 80% ACN/0.1% FA) and MS/MS spectra were collected by data‐dependent acquisition mode with the following setting; the most intense ions (Cycle Time: 3 s) with charge state +2 to +7 were selected for fragmentation by HCD with a normalized collision energy of 30, and detected using either Orbitrap or Ion Trap. Resolution and isolation window were set to 120,000 and 1.6 m/z, respectively. The data were analyzed using a Sequest HT search program in Proteome Discoverer 2.4 (Thermo Fisher Scientific). MS/MS spectra were searched against the SwissProt‐reviewed Homo sapiens reference proteome (UniProt v2017‐10‐25). Intensity‐based non‐label protein quantification was performed using a Precursor Ions Quantifier node in Proteome Discoverer 2.4. The mass tolerances for the precursor and fragment were 10 ppm and 0.02 Da (for Orbitrap) or 0.6 Da (for Ion Trap), respectively. Maximum missed cleavage sites of trypsin were set to 2. Oxidation (Met), GlyGly (Lys), deamidation (Gln, Asn), and acetylation (protein N terminus) were selected as variable modifications. Peptide identification was filtered at FDR < 0.01. The result of the shotgun MS is in Appendix Fig S3.

### Immunofluorescent staining, proximity ligation assay, and antibodies

After fixation in 4% paraformaldehyde/PBS and permeabilization, cells were incubated with antibodies diluted in PBS containing 1% BSA (Nacalai) and 0.02% Tween‐20 for 1 h. Nuclear staining was performed using DAPI (Dojindo). Images of stained cells were acquired with a laser confocal microscope (Carl Zeiss LSM710 and LSM800 or Olympus FV3000). In case of CXCL12 treatment, cells were cultured in DMEM supplemented with 0.05% BSA and treated with 100 ng/ml CXCL12. The proximity ligation assay (PLA) of transfected Cos7 cells was done using anti‐Halo rabbit antibody (Promega) and anti‐FLAG M2 mouse antibody (Sigma‐Aldrich) along with Duolink PLA kit (Sigma‐Aldrich) according to the manufacture’s protocol. The used antibodies were as follows: anti‐X123 (3C7), anti‐Epsin1 (C‐11), anti‐CXCR4 (12G5), and anti‐GAPDH (0411) antibodies (Santa‐Cruz Biotechnology); anti‐Clathrin (D3C6), anti‐Rab5 (E6N8S), anti‐Rab7 (D95F2), anti‐Rab11 (D4F5), anti‐ITCH (D8Q6D), anti‐HA tag, anti‐FLAG (DYKDDDDK), and anti‐Myc tag antibodies (Cell Signaling Technology); anti‐CXCR4 antibody (Abcam); anti‐ubiquitin (FK2) antibody (MBL); specific secondary antibodies conjugated to Alexa Fluor 350, 488, 555 or Alexa Fluor Plus 647 (Invitrogen).

### Chemotaxis assay and mammosphere assay

Chemotaxis assay was performed using Boyden chambers (BD Biosciences) as previously described (Inaguma *et al*, 2011). In brief, cells pre‐treated with either AMD3100 (SIGMA) or vehicle were resuspended in DMEM containing 0.5% BSA and seeded in the top chamber. Medium containing 100 ng/ml CXCL12 was added to the bottom chamber as an attractant. Twenty‐four hours (MCF‐7) or 18 h (4T1‐Luc) after application, migrated cells were stained and counted. For mammosphere assay, 3,000 cells were resuspended in 400 ml of MEBM basal medium (LONZA) supplemented with bovine pituitary extract, human epidermal growth factor, insulin, hydrocortisone, gentamicin, and amphotericin (SingleQuots supplement pack, LONZA) and cultured in 24‐well ultra‐low attachment plate (Costar). To estimate the effect of CXCL12/CXCR4 pathway, 100 ng/ml of CXCL12 and 12 mM of AMD3100 were applied. After 6 days, the number of mammosphere with a diameter more than 100 mm was counted.

### Database analyses and statistics

Gene expression data were obtained from the Oncomine database (http://www.oncomine.org). The protein structure of ENTREP was analyzed using the topology predictors Phobius (http://phobius.sbc.su.se/) (Kall *et al*, 2004) and CCTOP (http://cctop.enzim.ttk.mta.hu/) (Dobson *et al*, 2015). Phylogenetic analysis of FAM189s, NDFIP1, and N4BP1 was carried out using Phylogeny.fr (https://www.phylogeny.fr/) (Dereeper *et al*, 2008); in brief, the coding sequences were aligned (MUSCLE) and curated (Gblocks); a phylogenetic tree based on the maximum likelihood principle was then constructed from these sequences (PhyML‐aLRT) and visualized (TreeDyn). The impact of *ENTREP* expression on the long‐term prognosis of breast cancer patients was estimated in a dataset with Kaplan–Meier Plotter (https://kmplot.com/analysis/) (Gyorffy *et al*, 2010). Cutoff values were set to “auto select best cutoff”; all possible cutoff values between the lower and upper quartiles were tested, and the best‐performing threshold was used as the cutoff value. Statistical analysis was described in each of the figure legends.

## Author contributions

Takumi Tsunoda and KK conceived and designed the study. Takumi Tsunoda, NY, MS, MK, AI, and KK performed the experiments. MR, HI, KM, HM, and KK analyzed the data. Takuya Tomita, HT, and YS for ubiquitin‐AQUA/PRM and shotgun MS analyses. Takumi Tsunoda and KK wrote the manuscript. All the authors discussed the results and commented the manuscript.

## Supporting information



Appendix

Expanded View Figures PDF

## Data Availability

*ENTREP* cDNA sequence found in this study is available at the GenBank, the EMBL‐EBI, and the DDBJ under the accession number LC496047.1 (https://www.ncbi.nlm.nih.gov/nuccore/LC496047.1/). The result of yeast two‐hybrid screening has been submitted to the IMEx consortium through IntAct (Orchard *et al*, 2014) and assigned the identifier IM‐29244 (https://www.ebi.ac.uk/intact/imex/main.xhtml?query=IM-29244).
