# Supplementary material for: ENTREP/FAM189A2 encodes a new ITCH ubiquitin ligase activator that is downregulated in breast cancer
Source: EMBO Rep. 2021 Dec 20;23(2):e51182. doi: 10.15252/embr.202051182 (PMC8811627; doi:10.15252/embr.202051182)
Supplement: Supplementary file 1 — Appendix [file EMBR-23-e51182-s002.pdf]

Expanded View Level3 (Appendix) of

ENTREP/FAM189A2 downregulated in breast cancer encodes a new activator for ITCH ubiquitin ligase to regulate ubiquitination and endocytosis of CXCR4.

by Takumi Tsunoda, et al.

Table of contents

Appendix Fig S1. ENTREP/FAM189A2 and its exon 5-skipping variant.

Appendix Fig S2. Schematic presentation of expression vectors used in the study.

Appendix Fig S3. Shotgun MS analysis.

Appendix Fig S4. The imunoblot analyses of lentivirally transduced MCF-7 crispr cells.

Appendix Table S1. Lists of proteins identified by the yeast two-hybrid screening.

Appendix Table S2. Raw data of the ubiquitin-AQUA/PRM analysis.

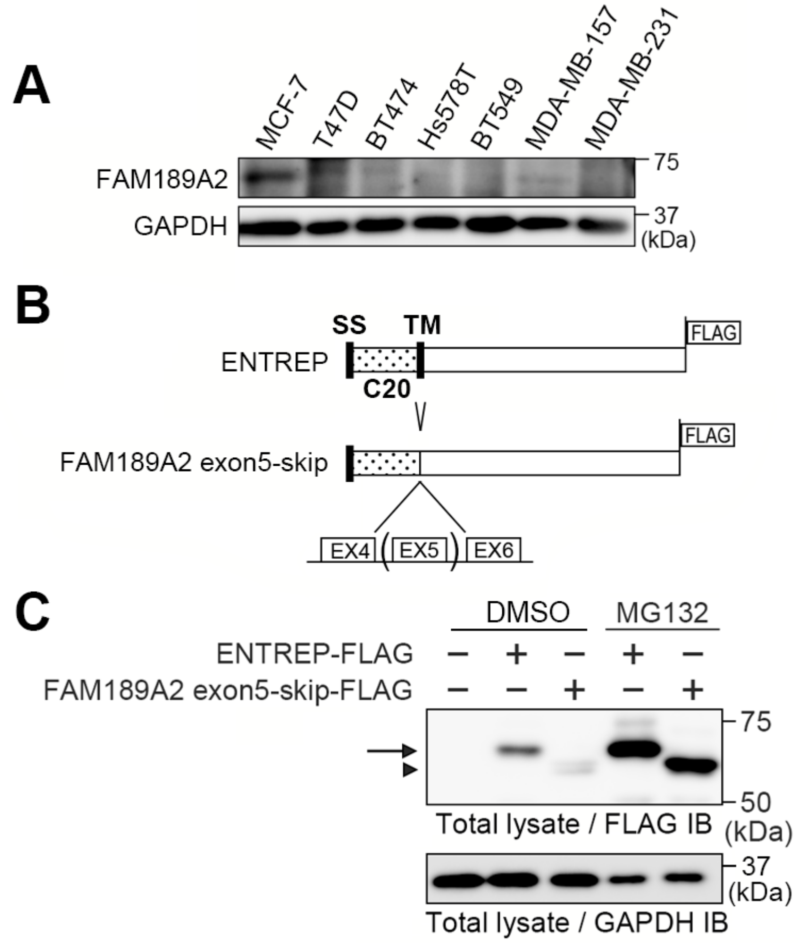

Appendix Fig S1. ENTREP/FAM189A2 and its exon 5-skipping variant.

A The immunoblot analyses of human breast cancer cell lines. Data shown are representative of at least three independent experiments. **B** Schema of ENTREP/ FAM189A2 and its exon 5-skipping variant. SS, signal sequence; CD20, CD20-homology extracellular domain; TM, transmembrane domain. **C** The immunoblot analyses using transiently transfected HEK293T cells. 24 hours after transfection, cells were treated with MG132 or its vehicle (DMSO) for 3 hours. *Arrow*, ENTREP-FLAG; *Arrowhead*, FAM189A2 exon5-skip-FLAG. Note that the volume of MG132-treated samples applied (at right two lanes) was a half of non-MG132 treated samples applied, as indicated by GAPDH. Data shown are representative of two independent experiments.

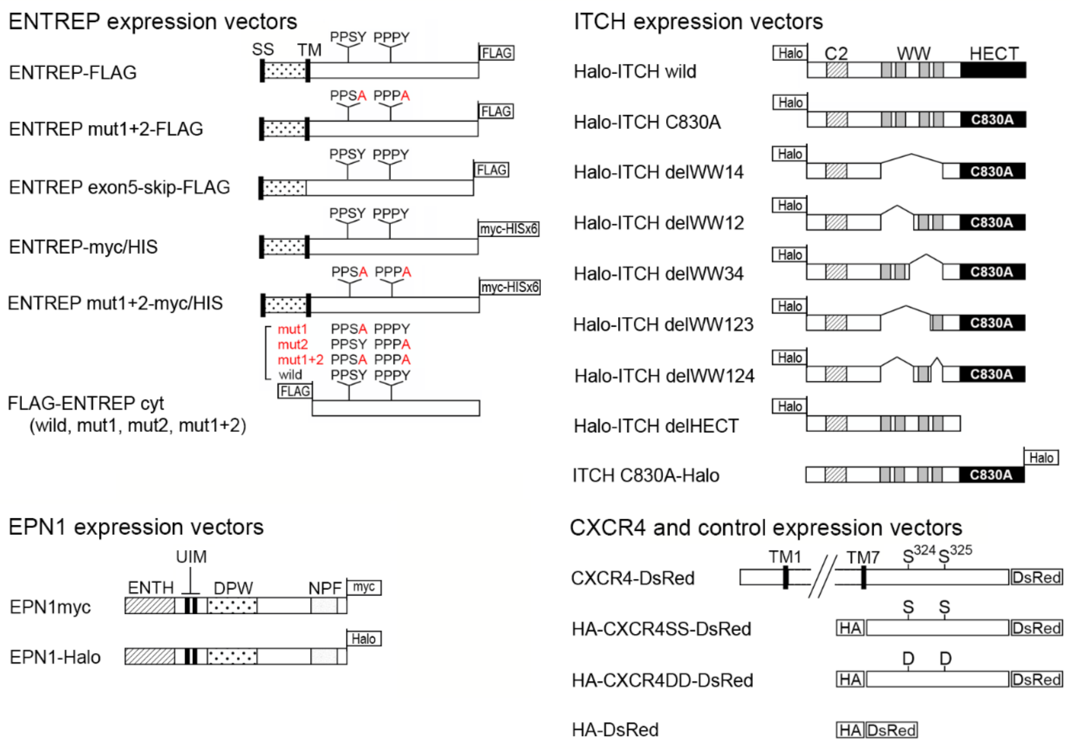

Appendix Fig S2. Schematic presentation of expression vectors used in the study. SS, signal sequence; CD20, CD20-homology extracellular domain; TM, transmembrane domain (ENTREP). ENTH, epsin N-terminal homology domain; UIM, ubiquitin-interacting motif; DPW, AP-2 binding Asp-Pro-Trp/Phe sequences; NPF, Asn-Pro-Phe triplets (EPN1). C2, C2 domain; WW, the tryptophan-tryptophan domain; HECT, the homologous to E6-AP carboxyl terminus domain (ITCH). TM1 and TM7, the first and seventh transmembrane domain, respectively (CXCR4).

Appendix Fig S3. Shotgun MS analysis.

#A of ENTREP

$^{273}SK_{GG}SDPVLHPSEER^{285}$

#1	b+	b2+	b3+	Seq.	y+	y2+	y3+	#2
1	88.03930	44.52329	30.01795	S				13
2	330.17719	165.59224	110.73058	K-GG	1507.74995	754.37861	503.25483	12
3	417.20922	209.10825	139.74126	S	1265.61206	633.30967	422.54220	11
4	532.23617	266.62172	178.08357	D	1178.58003	589.79365	393.53153	10
5	629.28893	315.14810	210.43449	P	1063.55308	532.28018	355.18921	9
6	728.35734	364.68231	243.45730	V	966.50032	483.75380	322.83829	8
7	841.44141	421.22434	281.15199	L	867.43191	434.21959	289.81549	7
8	978.50032	489.75380	326.83829	H	754.34784	377.67756	252.12080	6
9	1075.55308	538.28018	359.18921	P	617.28893	309.14810	206.43449	5
10	1162.58511	581.79619	388.19989	S	520.23617	260.62172	174.08357	4
11	1291.62770	646.31749	431.21409	E	433.20414	217.10571	145.07290	3
12	1420.67030	710.83879	474.22828	E	304.16155	152.58441	102.05870	2
13				R	175.11895	88.06311	59.04450	1

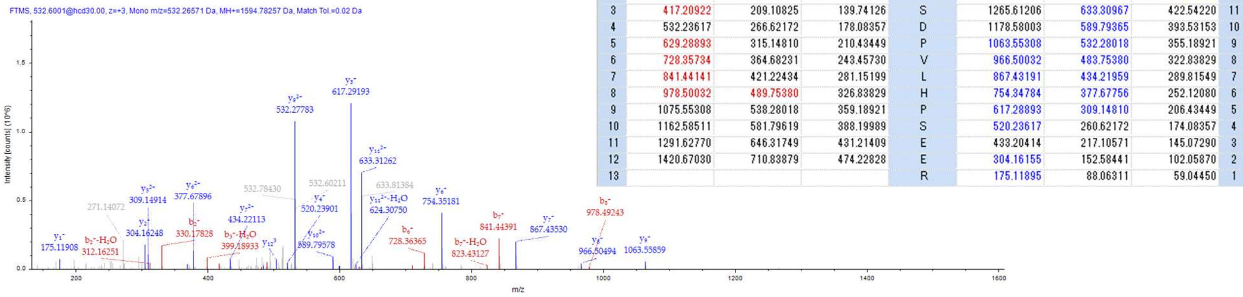

$^{324}SLIDYK_{GG}SYMDTK^{335}$

#1	b+	b2+	b3+	Seq.	y+	y2+	y3+	#2
1	88.03930	44.52329	30.01795	S				12
2	201.12337	101.06532	67.71264	L	1506.71447	753.86087	502.90967	11
3	314.20743	157.60735	105.40733	I	1393.63041	697.31884	465.21499	10
4	429.23438	215.12083	143.74964	D	1280.54634	640.77681	427.52030	9
5	592.29770	296.65249	198.10409	Y	1165.51940	583.26334	389.17798	8
6	834.43559	417.72144	278.81672	K-GG	1002.45607	501.73167	334.82354	7
7	921.46762	461.23745	307.82739	S	760.31818	380.66273	254.11091	6
8	1084.53095	542.76911	362.18183	Y	673.28615	337.14672	225.10024	5
9	1231.56635	616.28681	411.19363	M-Oxidat…	510.22283	255.61505	170.74579	4
10	1346.59329	673.80029	449.53595	D	363.18743	182.09735	121.73399	3
11	1447.64097	724.32412	483.21851	T	248.16048	124.58388	83.39168	2
12				K	147.11280	74.06004	49.70912	1

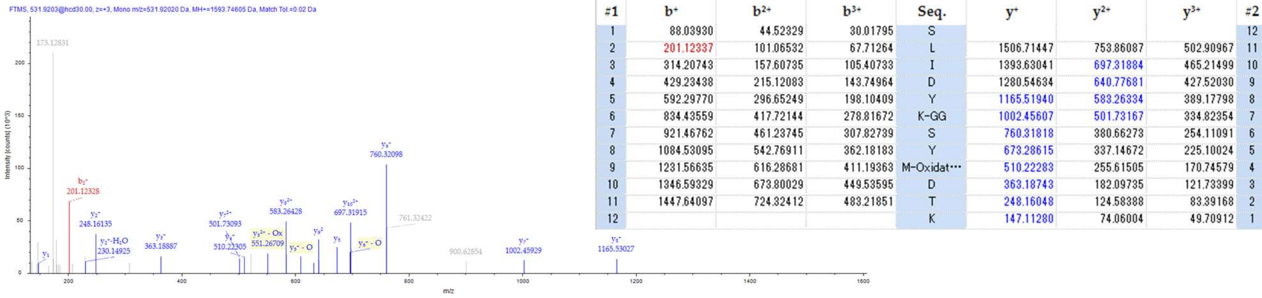

#B of ENTREP

$^{273}SK_{GG}SDPVLHPSEER^{285}$

#1	b+	b2+	b3+	Seq.	y+	y2+	y3+	#2
1	88.03930	44.52329	30.01795	S				13
2	330.17719	165.59224	110.73058	K-GG	1507.74995	754.37861	503.25483	12
3	417.20922	209.10825	139.74126	S	1265.61206	633.30967	422.54220	11
4	532.23617	266.62172	178.08357	D	1178.58003	589.79365	393.53153	10
5	629.28893	315.14810	210.43449	P	1063.55308	532.28018	355.18921	9
6	728.35734	364.68231	243.45730	V	966.50032	483.75380	322.83829	8
7	841.44141	421.22434	281.15199	L	867.43191	434.21959	289.81549	7
8	978.50032	489.75380	326.83829	H	754.34784	377.67756	252.12080	6
9	1075.55308	538.28018	359.18921	P	617.28893	309.14810	206.43449	5
10	1162.58511	581.79619	388.19989	S	520.23617	260.62172	174.08357	4
11	1291.62770	646.31749	431.21409	E	433.20414	217.10571	145.07290	3
12	1420.67030	710.83879	474.22828	E	304.16155	152.58441	102.05870	2
13				R	175.11895	88.06311	59.04450	1

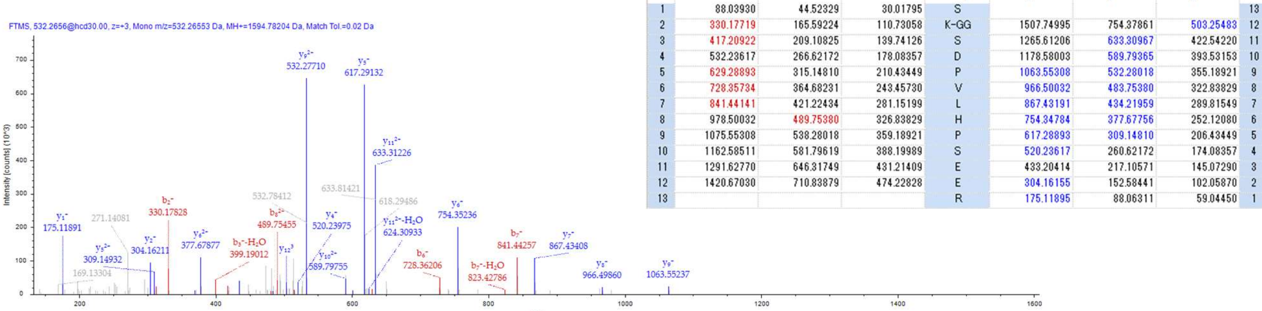

$^{324}SLIDYK_{GG}SYMDTK^{335}$

#1	b+	b2+	b3+	Seq.	y+	y2+	y3+	#2
1	88.03930	44.52329	30.01795	S				12
2	201.12337	101.06532	67.71264	L	1506.71447	753.86087	502.90967	11
3	314.20743	157.60735	105.40733	I	1393.63041	697.31884	465.21499	10
4	429.23438	215.12083	143.74964	D	1280.54634	640.77681	427.52030	9
5	592.29770	296.65249	198.10409	Y	1165.51940	583.26334	389.17798	8
6	834.43559	417.72144	278.81672	K-GG	1002.45607	501.73167	334.82354	7
7	921.46762	461.23745	307.82739	S	760.31818	380.66273	254.11091	6
8	1084.53095	542.76911	362.18183	Y	673.28615	337.14672	225.10024	5
9	1231.56635	616.28681	411.19363	M-Oxidat…	510.22283	255.61505	170.74579	4
10	1346.59329	673.80029	449.53595	D	363.18743	182.09735	121.73399	3
11	1447.64097	724.32412	483.21851	T	248.16048	124.58388	83.39168	2
12				K	147.11280	74.06004	49.70912	1

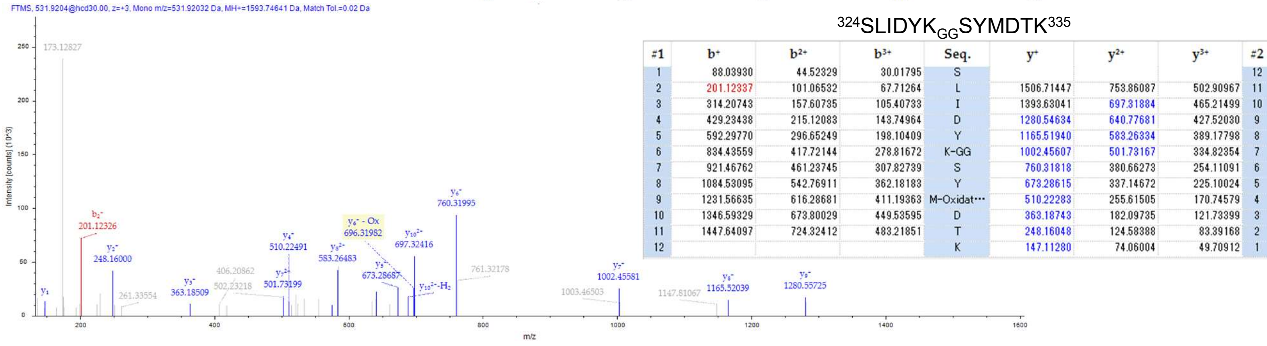

#C of ENTREP

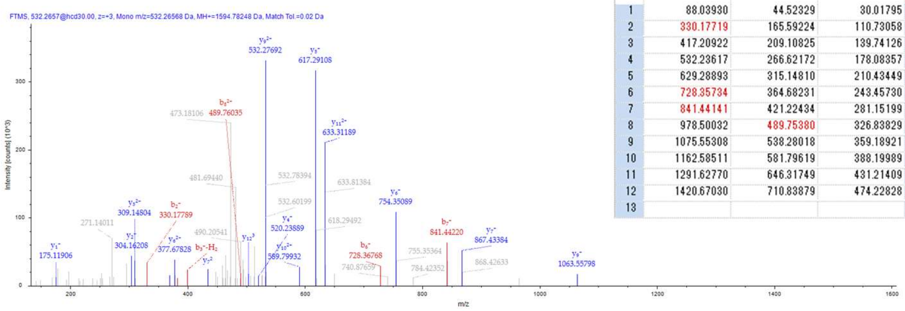

$^{273}SK_{GG}SDPVLHPSEER^{285}$

#1	b⁺	b²⁺	b³⁺	Seq.	y⁺	y²⁺	y³⁺	#2
1	88.03930	44.52329	30.01795	S				13
2	330.17719	165.59224	110.73058	K-GG	1507.74995	754.37861	503.25483	12
3	417.20922	209.10825	139.74126	S	1265.61206	633.30967	422.54220	11
4	532.23617	266.62172	178.08357	D	1178.58003	589.79365	393.53153	10
5	629.28893	315.14810	210.43449	P	1063.55308	532.28018	355.18921	9
6	728.35734	364.68231	243.45730	V	966.50032	483.75380	322.83829	8
7	841.44141	421.22434	281.15199	L	867.43191	434.21959	289.81549	7
8	978.50032	489.75380	326.83829	H	754.34784	377.67756	252.12080	6
9	1075.55308	538.28018	359.18921	P	617.28893	309.14810	206.43449	5
10	1162.58511	581.79619	388.19989	S	520.23617	260.62172	174.08357	4
11	1291.62770	646.31749	431.21409	E	433.20414	217.10571	145.07290	3
12	1420.67030	710.83879	474.22828	E	304.16155	152.58441	102.05870	2
13				R	175.11895	88.06311	59.04450	1

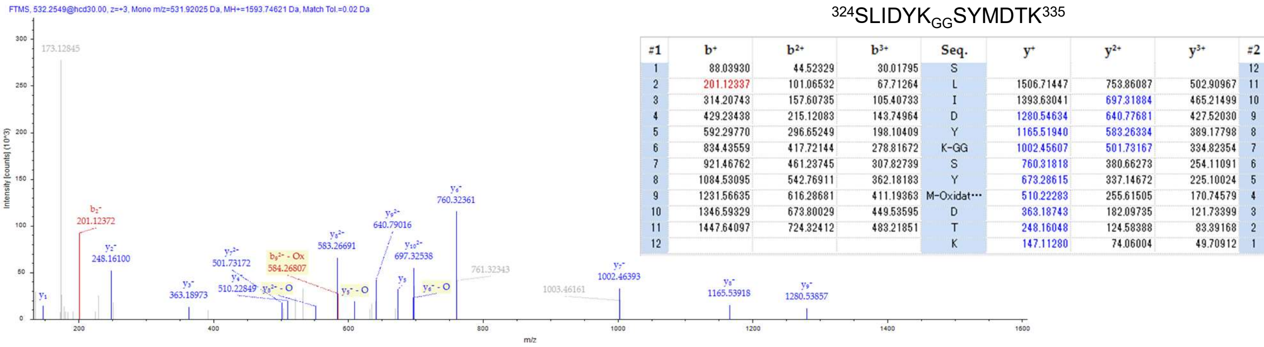

$^{324}SLIDYK_{GG}SYMDTK^{335}$

#1	b⁺	b²⁺	b³⁺	Seq.	y⁺	y²⁺	y³⁺	#2
1	88.03930	44.52329	30.01795	S				12
2	201.12337	101.06532	67.71264	L	1506.71447	753.86087	502.90967	11
3	314.20743	157.60735	105.40733	I	1393.63041	697.31884	465.21499	10
4	429.23438	215.12083	143.74964	D	1280.54634	640.77681	427.52030	9
5	592.29770	296.65249	198.10409	Y	1165.51940	583.26334	389.17798	8
6	834.43559	417.72144	278.81672	K-GG	1002.45607	501.73167	334.82354	7
7	921.46762	461.23745	307.82739	S	760.31818	380.66273	254.11091	6
8	1084.53095	542.76911	362.18183	Y	673.28615	337.14672	225.10024	5
9	1231.56635	616.28681	411.19363	M-Oxidat···	510.22283	255.61505	170.74579	4
10	1346.59329	673.80029	449.53595	D	363.18743	182.09735	121.73399	3
11	1447.64097	724.32412	483.21851	T	248.16048	124.58388	83.39168	2
12				K	147.11280	74.06004	49.70912	1

$^{362}SVLK_{GG}SDEEHMEEAITSASFLEQIMAPLQPSTSR^{394}$

#1	b⁺	b²⁺	b³⁺	b⁴⁺	Seq.	y⁺	y²⁺	y³⁺	y⁴⁺	#2
1	88.03930	44.52329	30.01795	22.76528	S					33
2	187.10772	94.05750	63.04076	47.53239	V	3721.75715	1861.38221	1241.25723	931.19474	32
3	300.19178	150.59953	100.73545	75.80340	L	3622.68873	1811.84800	1208.23443	906.42764	31
4	542.32967	271.66847	181.44808	136.33788	K-GG	3509.60467	1755.30597	1170.53974	878.15662	30
5	629.36170	315.18449	210.45875	158.09588	S	3267.46678	1634.23703	1089.82711	817.62215	29
6	744.38864	372.69796	248.80107	186.85262	D	3180.43475	1590.72101	1060.81643	795.86415	28
7	873.43124	437.21926	291.81526	219.11327	E	3065.40781	1533.20754	1022.47412	767.10741	27
8	1002.47383	501.74055	334.82946	251.37391	E	2936.36521	1468.68625	979.45992	734.84676	26
9	1139.53274	570.27001	380.51577	285.63864	H	2807.32262	1404.16495	936.44573	702.58611	25
10	1286.56814	643.78771	429.52756	322.39749	M-Oxidat···	2670.26371	1335.63549	890.75942	668.32138	24
11	1415.61073	708.30901	472.54176	354.65814	E	2523.22831	1262.11779	841.74762	631.56254	23
12	1544.65333	772.83030	515.55596	386.91879	E	2394.18572	1197.59650	798.73342	599.30189	22
13	1615.69044	808.34886	539.23500	404.67807	A	2265.14312	1133.07520	755.71923	567.04124	21
14	1728.77451	864.89089	576.92969	432.94908	I	2194.10601	1097.55664	732.04019	549.28196	20
15	1829.82218	915.41473	610.61225	458.21100	T	2081.02195	1041.01461	694.34550	521.01094	19
16	1916.85421	958.93074	639.62292	479.96901	S	1979.97427	990.49077	660.66294	495.74902	18
17	1987.89133	994.44930	663.30196	497.72829	A	1892.94224	946.97476	631.65226	473.99102	17
18	2074.92335	1037.96532	692.31264	519.48630	S	1821.90513	911.45620	607.97323	456.23174	16
19	2221.99177	1111.49952	741.33544	556.25340	F	1734.87310	867.94019	578.96255	434.47373	15
20	2335.07583	1168.04155	779.03013	584.52442	L	1587.80468	794.40598	529.93975	397.70663	14
21	2464.11842	1232.56285	822.04433	616.78506	E	1474.72062	737.86395	492.24506	369.43561	13
22	2593.16102	1297.08415	865.05852	649.04571	Q-Deami···	1345.67803	673.34265	449.23086	337.17496	12
23	2706.24508	1353.62618	902.75321	677.31673	I	1216.63543	608.82136	406.21666	304.91432	11
24	2853.28048	1427.14388	951.76501	714.07558	M-Oxidat···	1103.55137	552.27932	368.52197	276.64330	10
25	2924.31760	1462.66244	975.44405	731.83486	A	956.51597	478.76162	319.51017	239.88445	9
26	3021.37036	1511.18882	1007.79497	756.09805	P	885.47886	443.24307	295.83114	222.12517	8
27	3134.45442	1567.73085	1045.48966	784.36906	L	788.42609	394.71668	263.48022	197.86198	7
28	3262.51300	1631.76014	1088.17585	816.38371	Q	675.34203	338.17465	225.78553	169.59096	6
29	3359.56576	1680.28652	1120.52677	840.64690	P	547.28345	274.14536	183.09933	137.57632	5
30	3446.59779	1723.80253	1149.53745	862.40491	S	450.23069	225.61898	150.74841	113.31313	4
31	3547.64547	1774.32637	1183.22001	887.66683	T	363.19866	182.10297	121.73774	91.55512	3
32	3634.67750	1817.84239	1212.23068	909.42483	S	262.15098	131.57913	88.05518	66.29320	2
33					R	175.11895	88.06311	59.04450	44.53520	1

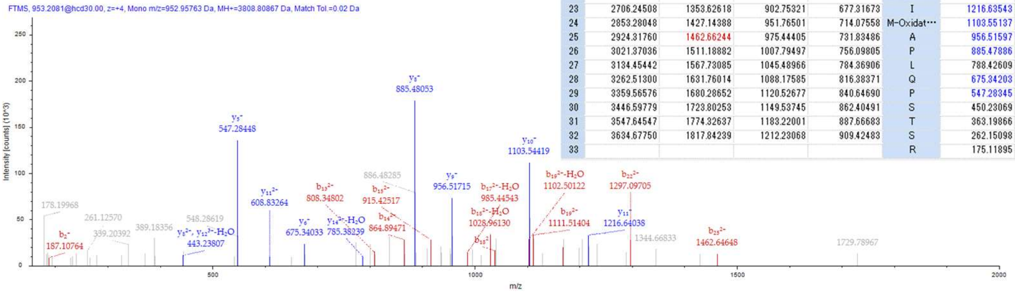

Appendix Fig S3. Shotgun MSanalysis.
Band #A, B and C of ENTREP were analyzed.
K274, K329 (#A-C) and K365 (#C) were ubiquitinated.

Tsunoda T, et al. Appendix Fig S4

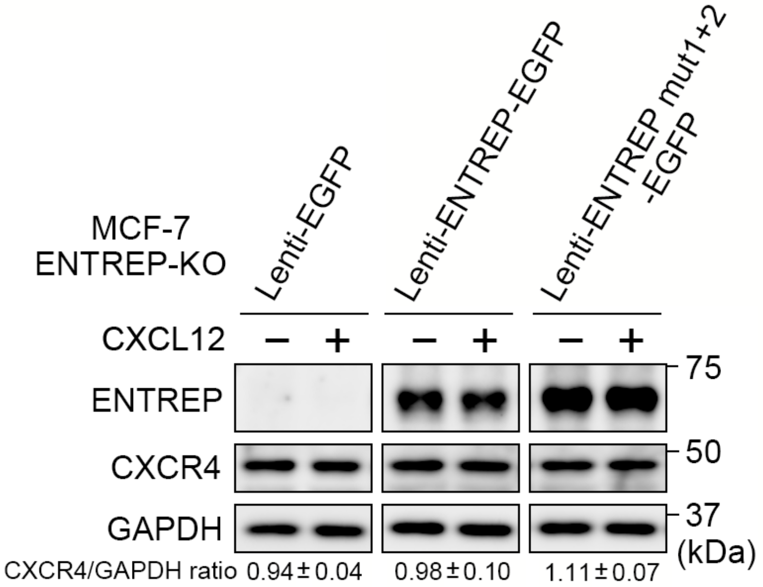

Appendix Fig S4. The imunoblot analyses of lentivirally transduced MCF-7 ENTREP-KO cells. MCF-7 ENTREP-KO cells were lentivirally transduced with one of ENTREP-EGFP, ENTREP mut1+2-EGFP, and control EGFP. Cells were treated with 100ng/ml CXCL12 or its vehicle for 1 hour and served for the immunoblot analyses. To detect ENTREP, anti-X123 3C7 antibody (Santa-Cruz Biotechnology) was used. The immunoblot analyses were carried out using six independent samples, and their blot bands were semi-quantified using ImageJ software. CXCR4/GAPDH ratio was calculated as a ratio of GAPDH-adjusted CXCR4 expression of CXCL12-treated sample to GAPDH-adjusted CXCR4 expression of the vehicle-treated sample. CXCR4/GAPDH ratios shown as mean ± SD (n=6). P-values were obtained by Student's t-tests and $P < 0.005$ was considered as statistically significant. No significant difference was observed.

Appendix Table S1. Lists of proteins identified by the yeast two-hybrid screening. The yeast two-hybrid screening was done with the amino acid 104-450 of ENTREP as a bait. Proteins identified in the human screen were ranked by predicted biological scores (PBS), provided by algorithms of Hybrigenics Services; A, very high confidence; B, high confidence; C, good confidence; D, moderate confidence, including potential false positives; E, interactions involving highly connected prey domains, warning of nonspecific interaction.

vector	Gene Name (Best Match)	GenBank ID (NCBI)	Gene ID (NCBI)	Global PBS
pB66	Homo sapiens - ANKRD13D	GID: 269315851	338692	D
pB66	Homo sapiens - ARIH1	GID: 187761372	25820	D
pB66	Homo sapiens - ASCC2	GID: 339275990	84164	C
pB27	Homo sapiens - BAG3	GID: 62530382	9531	D
pB66	Homo sapiens - DCUN1D1	GID: 809279622	54165	A
pB66	Homo sapiens - EPN1	GID: 194248094	29924	C
pB66	Homo sapiens - EPN2	GID: 156671216	22905	B
pB66	Homo sapiens - GRIPAP1	GID: 531034753	56850	C
pB66	Homo sapiens - HECW2	GID: 756140906	57520	D
pB66	Homo sapiens - HERC2	GID: 380420371	8924	B
pB66	Homo sapiens - HUWE1	GID: 531034782	10075	A
pB66	Homo sapiens - ITCH	GID: 380420334	83737	D
pB66	Homo sapiens - KHNYN	GID: 589908372	23351	D
pB66	Homo sapiens - LOC133957	GID: 34222184	133957	B
pB66	Homo sapiens - MAD2L2	GID: 187960072	10459	D
pB66	Homo sapiens - MYO6 var2	GID: 665505982	4646	A
pB66	Homo sapiens - NEDD4L	GID: 345478681	23327	D
pB66	Homo sapiens - OTUD4	GID: 767932132	54726	C
pB66	Homo sapiens - PAX6	GID: 386642908	5080	E
pB66	Homo sapiens - PCNXL3	GID: 574957105	399909	C
pB66	Homo sapiens - PIK3C2A	GID: 157671928	5286	D
pB66	Homo sapiens - PIK3C2B	GID: 194097346	5287	D
pB66	Homo sapiens - POLH	GID: 634743303	5429	D
pB66	Homo sapiens - POLI	GID: 154350219	11201	D
pB66	Homo sapiens - RNF115	GID: 523498727	27246	D
pB66	Homo sapiens - RNF123	GID: 375298724	63891	E
pB66	Homo sapiens - RNF168	GID: 300863109	165918	D
pB66	Homo sapiens - RNF216	GID: 319803088	54476	C
pB66	Homo sapiens - RPS27A	GID: 294459919	6233	A
pB66	Homo sapiens - SPRTN	GID: 766944320	83932	D
pB66	Homo sapiens - SYNE2	GID: 118918402	23224	D
pB66	Homo sapiens - TNIP1	GID: 356874785	10318	C
pB66	Homo sapiens - TRIM32	GID: 153792581	22954	C
pB66	Homo sapiens - TTRAP	GID: 23510347	51567	D
pB66	Homo sapiens - UBA52	GID: 77539054	7311	A
pB66	Homo sapiens - UBADC1	GID: 7705380	10422	D
pB66	Homo sapiens - UBB	GID: 528524469	7314	C
pB66	Homo sapiens - UBC	GID: 601984519	7316	A
pB66	Homo sapiens - UBQLN1 variant 1	GID: 194328681	29979	A
pB66	Homo sapiens - UBQLN4	GID: 747811795	56893	D
pB66	Homo sapiens - UBR5	GID: 544583485	51366	B
pB66	Homo sapiens - UBXN6	GID: 283806624	80700	D
pB66	Homo sapiens - UIMC1	GID: 313151223	51720	C
pB66	Homo sapiens - USF2	GID: 46877103	7392	D
pB66	Homo sapiens - USP13	GID: 215598687	8975	A
pB66	Homo sapiens - USP5	GID: 148727330	8078	B
pB66	Homo sapiens - WWC2	GID: 156546889	80014	D
pB66	Homo sapiens - WWP2	GID: 394581978	11060	D
pB66	Homo sapiens - YWHAE	GID: 195546907	7531	E
pB66	Homo sapiens - YWHAZ var2	GID: 208973236	7534	E

Appendix Table S2. Raw data of the ubiquitin-AQUA/PRM .

Raw Data (fmol)

	#A of control				#A of ENTREP				#B of control				#B of ENTREP				#C of control				#C of ENTREP			
	#1	#2	#3	average	#1	#2	#3	average	#1	#2	#3	average	#1	#2	#3	average	#1	#2	#3	average	#1	#2	#3	average
EST	0.80	0.21	0.22	0.41	78.80	14.59	14.45	35.95	0.36	0.31	0.18	0.28	78.03	29.63	29.73	45.80	0.79	0.40	0.20	0.46	55.10	22.08	23.95	33.71
K6	0.00	0.00	0.00	0.00	0.00	0.00	0.00	0.00	0.00	0.00	0.00	0.00	0.05	0.01	0.02	0.03	0.00	0.00	0.00	0.00	0.18	0.07	0.04	0.10
K11	0.01	0.01	0.06	0.03	0.05	0.03	0.03	0.04	0.01	0.02	0.01	0.01	0.17	0.06	0.08	0.10	0.03	0.02	0.01	0.02	0.55	0.16	0.16	0.29
K27	0.00	0.01	0.00	0.01	0.01	0.00	0.01	0.01	0.00	0.01	0.03	0.01	0.01	0.01	0.00	0.00	0.02	0.01	0.01	0.01	0.03	0.02	0.02	0.02
K29	0.01	0.00	0.00	0.00	0.01	0.09	0.01	0.03	0.01	0.00	0.07	0.03	0.03	0.02	0.14	0.06	0.00	0.01	0.00	0.00	0.14	0.03	0.10	0.09
K33	0.00	0.00	0.00	0.00	0.01	0.00	0.00	0.00	0.00	0.00	0.00	0.00	0.03	0.01	0.01	0.01	0.00	0.00	0.00	0.00	0.03	0.00	0.00	0.01
K48	0.05	0.01	0.01	0.02	0.39	0.05	0.07	0.17	0.01	0.00	0.00	0.00	2.45	0.73	1.03	1.40	0.04	0.02	0.00	0.02	3.31	1.02	0.95	1.76
K63	0.00	0.00	0.00	0.00	2.92	0.54	0.54	1.33	0.00	0.00	0.00	0.00	18.10	5.35	6.20	9.88	0.00	0.00	0.00	0.00	12.13	4.11	3.99	6.74
M1	0.00	0.00	0.00	0.00	0.00	0.00	0.00	0.00	0.00	0.00	0.00	0.00	0.00	0.00	0.00	0.00	0.00	0.00	0.00	0.00	0.00	0.00	0.00	0.00

Background Subtracted Data (fmol)

	#A of ENTREP				#B of ENTREP				#C of ENTREP			
	#1	#2	#3	average	#1	#2	#3	average	#1	#2	#3	average
EST	78.00	14.38	14.23	35.5367	77.67	29.32	29.55	45.5133	54.31	21.68	23.75	33.2467
K6	0	0	0	0	0.05	0.01	0.02	0.02667	0.18	0.07	0.04	0.09667
K11	0.04	0.02	0	0.02	0.16	0.04	0.072	0.09067	0.52	0.14	0.154	0.27133
K27	0.004	0	0.002	0.002	0.002	0	0	0.00067	0.01	0.01	0.014	0.01133
K29	0	0.09	0.007	0.03233	0.024	0.02	0.07	0.038	0.14	0.025	0.099	0.088
K33	0.01	0	0	0.00333	0.03	0.005	0.008	0.01433	0.03	0.002	0.002	0.01133
K48	0.34	0.04	0.06	0.14667	2.441	0.73	1.03	1.40033	3.27	1	0.948	1.73933
K63	2.92	0.54	0.54	1.33333	18.1	5.35	6.2	9.88333	12.13	4.11	3.99	6.74333
M1	0	0	0	0	0	0	0.004	0.00133	0	0	0	0
total Ub (EST+K63)	80.92	14.92	14.77	36.87	95.77	34.67	35.75	55.40	66.44	25.79	27.74	39.99
linkage sum	3.31	0.69	0.61	1.54	20.81	6.16	7.40	11.46	16.28	5.36	5.25	8.96
linkage percentage to total Ub	4.10	4.62	4.12	4.17	21.73	17.75	20.71	20.68	24.50	20.77	18.91	22.41
mono/end cap	77.61	14.23	14.16	35.33	74.96	28.52	28.35	43.94	50.16	20.43	22.49	31.03
mono/end cap to total Ub	95.90	95.38	95.88	95.83	78.27	82.25	79.29	79.32	75.50	79.23	81.09	77.59
