# Supplementary material for: ENTREP/FAM189A2 encodes a new ITCH ubiquitin ligase activator that is downregulated in breast cancer
Source: EMBO Rep. 2021 Dec 20;23(2):e51182. doi: 10.15252/embr.202051182 (PMC8811627; doi:10.15252/embr.202051182)
Supplement: Supplementary file 2 — Expanded View Figures PDF [file EMBR-23-e51182-s001.pdf]

Expanded View Figures

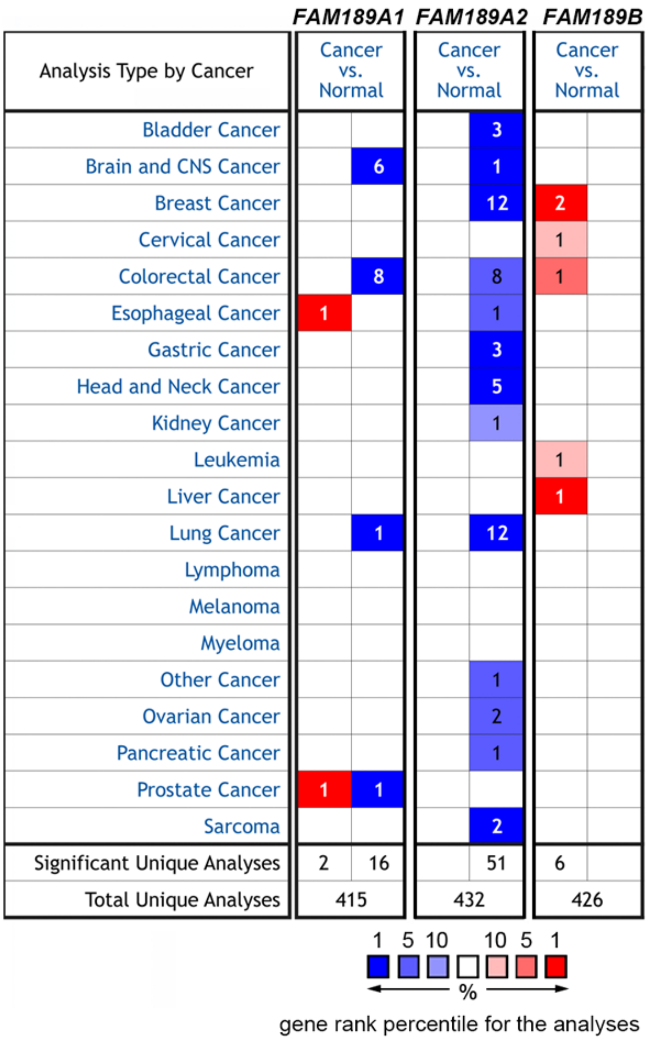

Figure EV1. The Oncomine database analyses of *FAM189A1*, *ENTREP/FAM189A2*, and *FAM189B* expression in various types of cancer.

Number indicates the number of datasets of gene expression analyses.

© 2021 The Authors

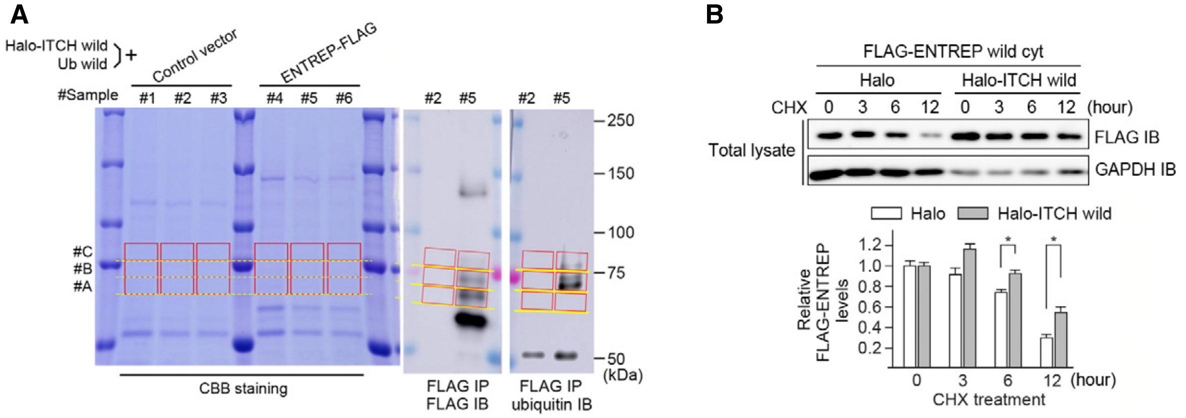

Figure EV2. Ubiquitination of ENTREP.

A Sample preparation for the ubiquitin-AQUA/PRM analysis. The immunoprecipitated samples using anti-FLAG antibody under the denaturing condition were separated by the SDS-PAGE and served for the immunoblot analyses and the Coomassie Brilliant Blue (CBB) gel staining. The gel area corresponding to the band #A, #B, and #C of the immunoblot were excised from the CBB-stained gel and used for ubiquitin-AQUA/PRM and shotgun MS analyses. The immunoblot image of FLAG IP/FLAG IB is the same with that of Fig 3B. We analyzed samples from three biological replicates (#1-3 of the control samples and #4-6 of ENTREP samples). The raw data of the ubiquitin-AQUA/PRM analysis are listed on Appendix Table S2 and the result of the shotgun MS is in Appendix Fig S3.

B The cycloheximide chase assay. Twenty-four hours after transfection, HEK293T cells were incubated with 50 mg/ml cycloheximide for the indicated time periods and served for the immunoblot analyses. The immunoblot analyses were carried out using six independent samples, and their blot bands were semi-quantified using ImageJ software. The relative FLAG-ENTREP expression levels were calculated as a ratio of GAPDH-adjusted FLAG-ENTREP at each time points and presented as a mean + SD from six biological replicates. *P*-values obtained by Student's *t*-tests and $P < 0.05$ was considered as statistically significant. *$P < 0.05$. Note that, in the immunoblot analyses, the volume of Halo-ITCH transfected samples applied was a half of Halo-transfected samples applied, as indicated by GAPDH.

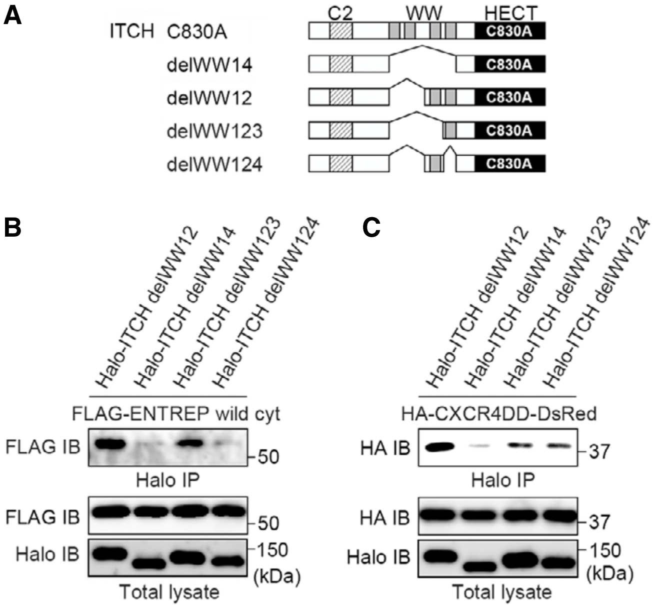

Figure EV3. The ITCH WW domain responsible for the association with ENTREP and CXCR4.

A Schema of the expression vectors of ITCH deletion mutants.

B, C The immunoprecipitation analysis. FLAG-ENTREP wild cyt co-precipitated with Halo-ITCH delWW123 but not with delWW124, whereas HA-CXCR4DD-DsRed co-precipitated equally with either Halo-ICTH delWW123 or delWW124. Data shown are representative of at least two independent experiments.

© 2021 The Authors

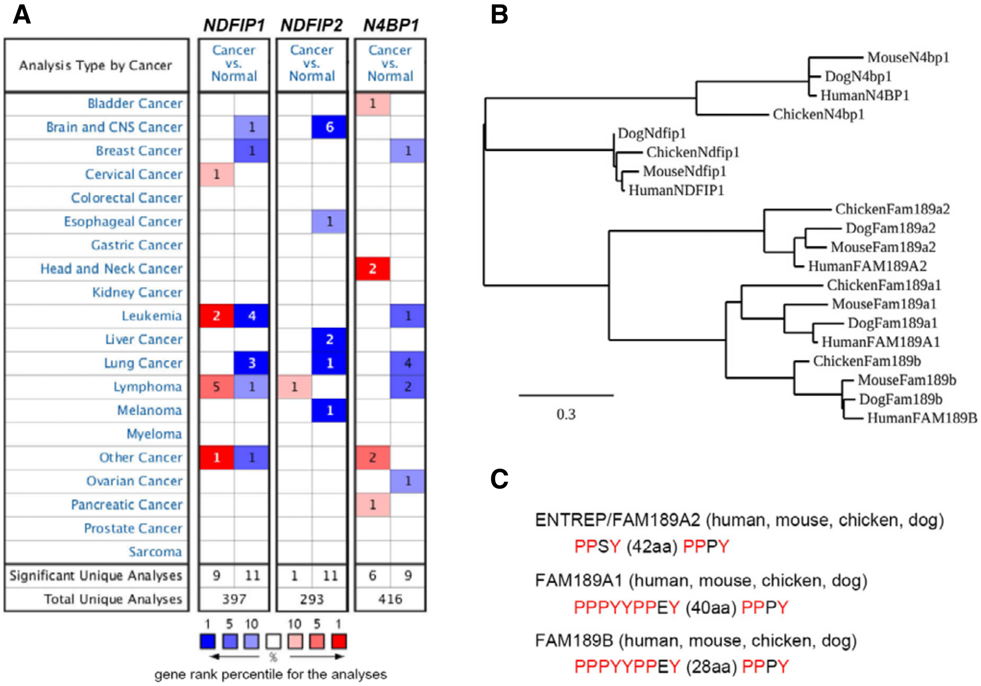

Figure EV4. Comparison of *NDFIP1*, *N4BP1*, and *FAM189 family*.

A The Oncomine database analysis of *NDFIP1*, *NDFIP2*, and *N4BP1* expression in various types of cancer. Number indicates the number of datasets of gene expression analyses.

B Phylogenetic analysis of *FAM189A1*, *ENTREP/FAM189A2*, *FAM189B*, *NDFIP1*, and *N4BP1*. The coding DNA sequences of these genes were analyzed using Phylogeny.fr software (https://www.phylogeny.fr/). Analyzed sequences were as follows: human *FAM189A1*, NM_015307.1; mouse *Fam189a1*, NM_183087.4; chicken *Fam189a1*, XM_025154007.1; dog *Fam189a1*, XM_025438066.1; human *FAM189A2*, NM_001127608.2; mouse *Fam189a2*, NM_001114174.1; chicken *Fam189a2*, XM_424828.6; dog *Fam189a2*, XM_022421428.1; human *FAM189B*, NM_006589.3; mouse *Fam189b*, NM_001014995.2; chicken *Fam189b*, XM_025143513.1; dog *Fam189b*, XM_005622739.2; human *N4BP1*, NM_153029.4; mouse *N4bp1*, NM_030563.2; chicken *N4bp1*, NM_001030570.1; dog *N4bp1*, XM_022411581.1; human *NDFIP1*, NM_030571.4; mouse *Ndfip1*, NM_001355749.1; chicken *Ndfip1*, XM_414658.5; dog *Ndfip1*, XM_022408883.1.

C The comparison of PPxY motif. FAM189A1 and FAM189B contain the overlapped PPxY sequences which are separated with PPPY by 40 aa and 28 aa, respectively.

© 2021 The Authors
