# Supplementary material for: ENTREP/FAM189A2 encodes a new ITCH ubiquitin ligase activator that is downregulated in breast cancer
Source: EMBO Rep. 2021 Dec 20;23(2):e51182. doi: 10.15252/embr.202051182 (PMC8811627; doi:10.15252/embr.202051182)
Supplement: Supplementary file 3 [file EMBR-23-e51182-s003.pdf]

EMBO reports

# *ENTREP/FAM189A2* encodes a new ITCH ubiquitin ligase activator that is downregulated in breast cancer

**Takumi Tsunoda, Miho Riku, Norika Yamada, Hikaru Tsuchiya, Takuya Tomita, Minako Suzuki, Mari Kizuki, Akihito Inoko, Hideaki Ito, Kenta Murotani, Hideki Murakami, Yasushi Saeki and Kenji Kasai**
**DOI: 10.15252/embr.202051182**

*Corresponding author(s): Kenji Kesai, Aichi Medical University, Japan (kkasai@aichi-med-u.ac.jp)*

## Review Timeline:

| | |
|---|---|
| Submission Date: | 26th Jun 20 |
| Editorial Decision: | 7th Aug 20 |
| Revision Received: | 30th Apr 21 |
| Editorial Decision: | 1st Jun 21 |
| Revision Received: | 9th Oct 21 |
| Editorial Decision: | 8th Nov 21 |
| Revision Received: | 13th Nov 21 |
| Accepted: | 29th Nov 21 |

*Editor: Deniz Senyilmaz Tiebe*

## Transaction Report:

(Note: With the exception of the correction of typographical or spelling errors that could be a source of ambiguity, letters and reports are not edited. Depending on transfer agreements, referee reports obtained elsewhere may or may not be included in this compilation. Referee reports are anonymous unless the Referee chooses to sign their reports.)

**1st Editorial Decision** **7th Aug 2020**

Dear Prof. Kasai,

Thank you for the submission of your research manuscript to our journal, which was now seen by three referees, whose reports are copied below.

We concur with the referees that proposed role of ENTREP in regulation of CXCR4 endocytosis via ITCH mediated ubiquitination is in principle very interesting. However, referees also raise important concerns that need to be addressed to consider publication here.

I find the reports informed and constructive, and believe that addressing the concerns raised will significantly strengthen the manuscript.

Given these positive recommendations, we would like to invite you to revise your manuscript with the understanding that the referee concerns (as in their reports) must be fully addressed and their suggestions taken on board. Please address all referee concerns in a complete point-by-point response. Acceptance of the manuscript will depend on a positive outcome of a second round of review. It is EMBO reports policy to allow a single round of revision only and acceptance or rejection of the manuscript will therefore depend on the completeness of your responses included in the next, final version of the manuscript.

We generally allow three months as standard revision time. As a matter of policy, competing manuscripts published during this period will not negatively impact on our assessment of the conceptual advance presented by your study. However, we request that you contact the editor as soon as possible upon publication of any related work, to discuss how to proceed. Should you foresee a problem in meeting this three-month deadline, please let us know in advance and we may be able to grant an extension.

*** Temporary update to EMBO Press scooping protection policy:
We are aware that many laboratories cannot function at full efficiency during the current COVID-19/SARS-CoV-2 pandemic and have therefore extended our 'scooping protection policy' to cover the period required for a full revision to address the experimental issues highlighted in the editorial decision letter. Please contact the scientific editor handling your manuscript to discuss a revision plan should you need additional time, and also if you see a paper with related content published elsewhere.***

IMPORTANT NOTE: we perform an initial quality control of all revised manuscripts before re-review. Your manuscript will FAIL this control and the handling will be DELAYED if the following APPLIES:
1. A data availability section providing access to data deposited in public databases is missing (where applicable).
2. Your manuscript contains statistics and error bars based on n=2. Please use scatter plots in these cases.

Supplementary/additional data: The Expanded View format, which will be displayed in the main HTML of the paper in a collapsible format, has replaced the Supplementary information. You can submit up to 5 images as Expanded View. Please follow the nomenclature Figure EV1, Figure EV2 etc. The figure legend for these should be included in the main manuscript document file in a section called Expanded View Figure Legends after the main Figure Legends section. Additional Supplementary material should be supplied as a single pdf labeled Appendix. The Appendix includes a table of content on the first page with page numbers, all figures and their legends. Please follow the nomenclature Appendix Figure Sx throughout the text and also label the figures according to this nomenclature. For more details please refer to our guide to authors.

Please note that for all articles published beginning 1 July 2020, the EMBO Reports reference style will change to the Harvard style for all article types. Details and examples are provided at
https://www.embopress.org/page/journal/14693178/authorguide#referencesformat

When submitting your revised manuscript, please carefully review the instructions that follow below. Failure to include requested items will delay the evaluation of your revision.

1) a .docx formatted version of the manuscript text (including legends for main figures, EV figures and tables). Please make sure that the changes are highlighted to be clearly visible.

2) individual production quality figure files as .eps, .tif, .jpg (one file per figure).

3) a .docx formatted letter INCLUDING the reviewers' reports and your detailed point-by-point responses to their comments. As part of the EMBO Press transparent editorial process, the point-by-point response is part of the Review Process File (RPF), which will be published alongside your paper. For more details on our Transparent Editorial Process, please visit our website:
https://www.embopress.org/page/journal/14693178/authorguide#transparentprocess
You are able to opt out of this by letting the editorial office know (emboreports@embo.org). If you do opt out, the Review Process File link will point to the following statement: "No Review Process File is available with this article, as the authors have chosen not to make the review process public in this case."

4) a complete author checklist, which you can download from our author guidelines (<http://embor.embopress.org/authorguide>). Please insert information in the checklist that is also reflected in the manuscript. The completed author checklist will also be part of the RPF.

5) Please note that all corresponding authors are required to supply an ORCID ID for their name upon submission of a revised manuscript (<https://orcid.org/>). Please find instructions on how to link your ORCID ID to your account in our manuscript tracking system in our Author guidelines (<http://embor.embopress.org/authorguide>).

6) We replaced Supplementary Information with Expanded View (EV) Figures and Tables that are collapsible/expandable online. A maximum of 5 EV Figures can be typeset. EV Figures should be cited as 'Figure EV1, Figure EV2" etc... in the text and their respective legends should be included in the main text after the legends of regular figures.

- For the figures that you do NOT wish to display as Expanded View figures, they should be bundled together with their legends in a single PDF file called *Appendix*, which should start with a short Table of Content. Appendix figures should be referred to in the main text as: "Appendix Figure S1, Appendix Figure S2" etc. See detailed instructions regarding expanded view here: <http://embor.embopress.org/authorguide#expandedview>.

- Additional Tables/Datasets should be labeled and referred to as Table EV1, Dataset EV1, etc. Legends have to be provided in a separate tab in case of .xls files. Alternatively, the legend can be supplied as a separate text file (README) and zipped together with the Table/Dataset file.

7) We would also encourage you to include the source data for figure panels that show essential data.

Numerical data should be provided as individual .xls or .csv files (including a tab describing the data). For blots or microscopy, uncropped images should be submitted (using a zip archive if multiple images need to be supplied for one panel). Additional information on source data and instruction on how to label the files are available <http://embor.embopress.org/authorguide#sourcedata>.

8) Our journal encourages inclusion of *data citations in the reference list* to directly cite datasets that were re-used and obtained from public databases. Data citations in the article text are distinct from normal bibliographical citations and should directly link to the database records from which the data can be accessed. In the main text, data citations are formatted as follows: "Data ref: Smith et al, 2001" or "Data ref: NCBI Sequence Read Archive PRJNA342805, 2017". In the Reference list, data citations must be labeled with "[DATASET]". A data reference must provide the database name, accession number/identifiers and a resolvable link to the landing page from which the data can be accessed at the end of the reference. Further instructions are available at <http://embor.embopress.org/authorguide#datacitation>.

9) Please make sure to include a Data Availability Section before submitting your revision - if it is not applicable, make a statement that no data were deposited in a public database. Primary datasets (and computer code, where appropriate) produced in this study need to be deposited in an appropriate public database (see <http://embor.embopress.org/authorguide#dataavailability>).

Please remember to provide a reviewer password if the datasets are not yet public.

The accession numbers and database should be listed in a formal "Data Availability " section (placed after Materials & Method) that follows the model below. Please note that the Data Availability Section is restricted to new primary data that are part of this study.

# Data availability

The datasets (and computer code) produced in this study are available in the following databases:

- RNA-Seq data: Gene Expression Omnibus GSE46843 (https://www.ncbi.nlm.nih.gov/geo/query/acc.cgi?acc=GSE46843)
- [data type]: [name of the resource] [accession number/identifier/doi] ([URL or identifiers.org/DATABASE:ACCESSION])

*** Note - All links should resolve to a page where the data can be accessed. ***

10) Regarding data quantification, please ensure to specify the name of the statistical test used to generate error bars and P values, the number (n) of independent experiments underlying each data point (not replicate measures of one sample), and the test used to calculate p-values in each figure legend. Discussion of statistical methodology can be reported in the materials and methods section, but figure legends should contain a basic description of n, P and the test applied.

Please note that error bars and statistical comparisons may only be applied to data obtained from at least three independent biological replicates.
Please also include scale bars in all microscopy images.

We would also welcome the submission of cover suggestions, or motifs to be used by our Graphics Illustrator in designing a cover.

I look forward to seeing a revised version of your manuscript when it is ready. Please let me know if you have questions or comments regarding the revision.

Yours sincerely,

Deniz Senyilmaz Tiebe

Deniz Senyilmaz Tiebe, PhD
Editor
EMBO Reports

Referee #1:

This manuscript reports the observation that FAM189A2/ENTREP downregulation impacts the prognosis of breast cancer patients. It further identifies FAM189A2/ENTREP as an interactor of the ubiquitin ligase ITCH and the endocytic adaptor EPN1 and characterizes the interactions, localization, and functional interplay between these proteins, along with the chemokine receptor CXCR4, which, like ENTREP, is a substrate of ITCH.
The manuscript, while interesting, is rather awkwardly written. This is reflected in a number of grammatical and structural issues that need careful editing. Specifically, a lot of information that ought to be in the introduction is mentioned in the results section. In addition, I feel that the functional/cell-based claims made in this manuscript should be consolidated with orthogonal techniques. Furthermore, the conclusion that ITCH forms non-degradative, K63-linked chains on CXCR4 and EPN1 is not sufficiently back up by data. In sum, this manuscript requires considerable improvement in order to meet the rigorous standards of EMBO Rep.

Specific comments:

Introduction:
- Overall, the introduction is rather short and not particularly informative. Apart from the last paragraph, which is a summary of the new findings, there are just a few rather general sentences leading to the question studied. I feel that the introduction needs to be significantly more detailed and provide a more specific expert introduction to ITCH/FAM189A2 and the cellular context studied.

- The authors should rephrase the sentence stating that E3 ligases function at the "most critical step" of the ubiquitination cascade. Each step is necessary and thus critical. Maybe the authors intended to refer to specificity?

- The authors appear to state that U-box ligases only occur in Arabidopsis, which is incorrect.

- The authors should say that ligases are classified into 4 main types (rather than "4 types"), since they do not mention the recently discovered RCR ligase (of which it is yet unclear whether it is a class or a single member).

- When introducing the fact that HECT ligases can be allosterically autoinhibited, the authors should also Chen et al., Mol Cell, 2017 (for WWP2), Sander et al., eLife 2017 (for HUWE1), and Attali et al., EMBO J 2017 (for NEDD4).

Results:

- Some of the information that is missing in the introduction appears within the results section. This information should be moved. For example, on page 6, there is some useful information on the NEDD4-subfamily and ENP1/2 that should have been mentioned in the intro. Likewise, the paragraph at the end of page 8 (continuing on page 9) belongs into the introduction etc.

- Figure 2C:
This experiment is performed under non-denaturing conditions. As such, it is not clear whether the observed smears result from co-IPed, ubiquitinated proteins or represent ubiquitinated forms of ENTREP. The authors need to state this - AND/OR - perform the experiment with His-tagged ubiquitin and IP under denaturing conditions (i.e. Nickel-pulldown, followed by Western against the substrate). Furthermore, in the current figure, the authors should show the total lysate FLAG-IB as a control.

The authors conclude that ubiquitination occurs mainly through K63. While plausible from the literature, this is not visible in their experiment, since there is a clear difference between the ubiquitin WT and K63-only lanes. From this experiment, one can only conclude that more ubiquitination occurs through K63 than K48. Obviously, there are either additional lysines involved in the modification and/or the mutation of 6 lysines at once in the K63-only and K48-only mutants, respectively, affects activity. These options should be investigated by using individual K/R-mutants.
Also, more experiments should be done to support the hypothesis that "Active ITCH ubiquitinates ENTREP primarily through Lys63-linked ubiquitination, which may protect ENTREP protein from degradation and further enhance its association with EPN1." (see discussion). Such experiments could include: cycloheximide-chase experiments to confirm that the ITCH-mediated ubiquitination of ENTREP is not degradative; and the above-mentioned experiments with Lys-to-Arg mutants of ubiquitin to demonstrate that the chains are indeed primarily linked via Lys63.

- Figure 2 D:
The expression of Flag-ENTREP as well as myc-EPN1 is overall higher in +ITCH setup (see input). This may explain higher levels of EPN1 in myc-pulldown instead of ITCH having an effect. Therefore, I recommend to include catalytically dead ITCH to ensure that it is the ubiquitination of ENTREP that enhances binding.
Why is there a smear in the no-ITCH lane (FLAG IP, HA IB)? The authors should comment on the possibility of ubiquitination by endogenous ITCH or other E3s.
Also, marker positions should be indicated.

- In general, it would be helpful if the authors commented on their choice of cell lines. Why Cos7 cells on page 7 row 25? Why MCF-7 cells instead of HMEC on page 5 row 22.

- Figure 3C:
The input of Halo-ITCH is lower for delW14 and much lower for delW34, That CXCR4 favours WW34 is thus not convincingly confirmed. Thus, the authors should show equal levels or adjust their interpretation.

- Fig. 4 C:
I am not convinced that the amount of CXCR4 in the cytoplasm is "substantial"

- Figure 3 F:
Here, the same criticism applies as to 2C. The authors should transfect His-Ubi and perform a Nickel-pull down under denaturing conditions in order to separate ubiquitination of CXCR4 from potentially ubiquitinated interaction partners.
Further, the authors state that Fig 3F shows that "ITCH did not appreciably ubiquitinate CXCR4" (page 10). I wonder whether this statement also holds for monoubiquitinated CXCR4, since the blot is cropped just below 50 kDa. If no, the authors should replace the word "ubiquitinate" by "polyubiquitinate"

- Figure 5C:
How can both ENTREP and ITCH occupy the same WW domain of ITCH (which they must since CXCR4 is co-precipitated in ENTREP-pulldowns through ITCH)? How do the authors think about this? Please elaborate on this in the discussion.

- In general, I feel that the IF data should be either quantified or confirmed by an orthogonal technique, such as proximity ligation, crosslinking mass spec etc..

Discussion

There is no clear separation between the results and discussion part, which should be indicated.

Methods

The authors should provide more detail on the position of the tags in the individual constructs. This is particularly necessary for the HECT constructs, since these ligases are known to be sensitive to C-terminal tagging.

Referee #2:

In this study, Tsunoda et al. identify the previously uncharacterised protein FAM189A2 as a regulator of the HECT E3 ligase ITCH. In an earlier study the authors had identified FAM189A2 as a down regulated gene in several types of breast cancer (Riku et al., 2016).

In the current study, the authors propose that FAM189A2 recruits ITCH to the plasma membrane and enhances ITCH-mediated ubiquitination of the chemokine receptor CXCR4. Furthermore, ubiquitination of FAM189A2 by ITCH is suggested to enhance the association of FAM189A2 with EPN1, promoting endocytosis of the FAM189A2-ITCH-CXCR4 complex. As a result, loss of FAM189A2 function is linked to enhanced chemotaxis and stemness of MCF-7 breast cancer cells. In line with this model, the authors propose renaming FAM189A2 to Endosomal Transmembrane binding with EPsin (ENTREP).

While this study is conceptually interesting, we feel that part of the model is not sufficiently supported by the current experimental data presented, and thus suggest further basic experimentation to clarify key points.

Notably the authors describe ENTREP as an 'adaptor' of ITCH. In our view, the term adaptor implies that ENTREP mediates the interaction between two proteins, in this case ITCH and CXCR4 - by binding to both simultaneously. Instead the proposed model (Fig 5C) shows that ENTREP binds to and recruits ITCH to the membrane and activates ITCH - but does not bind to CXCR4. More appropriately, ENTREP might be considered an 'activator' or 'recruiter' of ITCH, although neither of these functions are explicitly demonstrated by the current data.

Major points
1) Based on the model, ITCH should be recruited to the plasma membrane in an ENTREP-dependent manner. Is this indeed the case in cells? The authors should be able to use their MCF-7crispr k/o cell line or the ITCH (delWW14) or ENTREP (mut1+mut2) mutants to investigate this point. These experiments may require the use of the ITCH active site mutant or an inhibitor to block downstream signalling and endocytosis.
2) Many of the experiments imply that the observed effects and ubiquitination are mediated by ITCH E3 ligase activity. However, this could potentially also be mediated by other E3 ligases in the cell, or by a scaffolding function of ITCH. The authors should include the ITCH C830A active site mutant in all of the relevant experiments (Figs. 2C, 2D, 2E, 3F) to exclude these possibilities.
3) The study mainly relies on overexpression experiments using an ENTREP construct that only contains the cytoplasmic region of the protein. This artificial system may heavily affect localisation and function of ENTREP, a transmembrane protein in nature.
4) The authors show that ITCH, ENTREP, EPN1 and CXCR4 form a stable complex. For example, they are able to pull-down Halo-ITCH and CXCR4 with an anti-FLAG antibody directed against FLAG-ENTREP (Fig 3D). This implies that all the pull-downs contain a mixture of these and potentially other proteins. In the ubiquitination experiments (Figs. 2C, 2D and 3F), the authors only blot for HA-Ub but not for the proposed substrate. The authors should include blots showing molecular weight shift of these substrates.

Minor points
1) ITCH is known to form K29, 48 and K63 ubiquitin chains. However, the authors only test for K48 and K63 chains in Fig 2C. They should include a K29 only ubiquitin mutant.
2) Fig 2D lane 3 (FLAG-ENTREP/HALO/EPN1myc/HA-ubiquitin) shows a HA-ubiquitin smear indicating E3 ligase activity. This is different to Fig 2C lane 3, which almost shows the same conditions; the only difference being absence of EPN1myc in Fig2C. Is this a real effect potentially mediated by EPN1 or an experimental artifact?
3) The colocalization in Fig 2E is not very convincing as the overexpressed proteins are present in the entire cell. Also, in light of our major point 3 above, can the authors visualise endogenous protein or use a lower level of overexpression? It is also unclear where the insets have been taken from. This should be indicated.
4) Fig 3C: The HALO-ITCH expression levels vary heavily between the different mutants and seem to correlate with the amount of protein pulled down. This experiment should be repeated with more consistent expression levels. Fig 3D shows that more comparable expression of these constructs can be achieved.
5) Fig 3E: The authors imply that increased ENTREP expression leads to increased ENTREP binding to ITCH, which in turn leads to increased CXCR4 recruitment. While the latter is evident from this figure, the authors should also blot for FLAG-ENTREP to show the first point.
6) Fig 4C: How does the GFP-ENTREP overexpression compare to the endogenous ENTREP expression levels in wild-type cells?
7) Fig 5A: Individual data points should be included in these graphs.
8) Materials and methods for the pull downs and Western blots need to be included.
9) Most of the blots do not contain MW markers. These need to be added.
10) The blots are heavily cropped and full blots should be shown in a supplement.
11) The HALO blots are often split to show HALO-ITCH in one strip and HALO alone in another strip. The authors should consider showing this in a single frame. This is particularly confusing in Fig 3D, where the 2nd FLAG IP/Halo IB strip is completely empty.
12) The error bars in Fig 1A are not defined.
13) Typos:
a. Fig 2C: 'ENP1myc' should be 'EPN1myc'
b. Page 10: "NDFIP1 binds the WW domain of ITCH, opens its intermolecular structure" - should read "intramolecular"

Referee #3:

In this manuscript, its is suggested that the gene product FAM189A2 (termed here as ENTREP) associate with the C2-WW-HECT-containing E3 ubiquitin ligase ITCH and the endocytic-marker epsin1/2 (EPIN) with the overall effect to promote the ubiquitination, vesicular trafficking and degradation of the ITCH substrate CXCR4. Functionally, ENTREP is suggested to promote the ability of ITCH to downregulate the ability of CXCL12-CXCR4 signalling axis to promote chemotaxis and anchorage-independent cell growth of breast cancer cells. To provide support of the suggested conclusions, additional controls and experiments are required.

Specific comments:

1. Figure 1A, mRNA? Protein level? MCF7 higher mRNA level, is that reflected at the protein level? The molecular subtypes of all cancer cell lines should be included in the manuscript. What is known about itch and CXCR4 expression in these cell lines.
2. The implication of ENTREP correlation with RFS but not with overall survival should be alluded to in the discussion. The term "intrinsic subtypes" in paragraph 1 of page 5 should be defined/descried
3. Is the exon 5-less ENTREP isoform expressed at the protein level? if so, does it differ in function from TM-containing version? At least this point should be a point of speculation in the discussion. In this regard, is this the isoform used in the biochemical interaction studies?
4. The yeast two-hybrid screen identified many interactors for the cytoplasmic version of ENTREP which was used as the bait. A rationale should be provided for the focus on testing the hits ITCH and EPIN1/2 for further analyses.
5. To confirm yeast two hybrid-results, coips were performed on cells overexpressing the proteins being investigated. It is not clear why the TM-containing ENTREP protein is not used in these coips. In terms of WW-domain-dependent interactions, are each or both of WW1 and 4 required equivalently for such interactions? Overall, it would be important to investigate/confirm ENTREP-ITCF-EPN1 association at endogenous level of these proteins.
6. The Lysine residues and the nature of the mutation for the HA-ubiquitin should be clearly stated in figure 2C. Inclusion of a ubiquitin with all lysine residues converted arginine to prevent poly-ubiquitination of a substrate would be recommended as a negative control in figure 2C. Data showing the ubiquitination assays in which cells are not treated with MG132 will help reveal the importance of ubiquitination for ENTREP protein stability. In this regard, it is not clear how ENTREP stabilization by ITCH was assessed. In figure 2C, it appears that Ub(K48) to be involved in polyubiquitination of ENTREP. In addition, K63 does not appear to be sufficient to generate the same degree of poly-ubiquitination as that of wild type ubiquitin. These data and implication should be discussed. Overall, a FLAG immunoblotting of the ENTREP immunocomplexes (anti-FLAG IP) should be shown and used in the normalization of the ubiquitinated species to that of unmodified ENTREP for each FLAG-immunoprecipitated lysate sample. Relating to figure 2D, as ITCH1 expression seems to increase the protein level of EPN1 and ENTREP, is the apparent increase in the ENTRP-EPN1 association under such condition largely due to an increase in their abundance? An ITCH-interaction mutant ENTREP (YA) should be tested for ability to coip EPN1, in the presence and absence of ITCH, to provide further evidence for the conclusion that ITCH-mediated ubiquitination of ENTREP promotes its association with EPN1. Inclusion of ITCH and ENTREP without EPN1 should be considered as a control, especially for assessing the HA/ubiquitination signal of the FLAG IP. Results of the immunofluorescence experiments and suggested colocalization/trafficking presented in figures 2E and 2F are hard to ascertain an should be subjected to quantitative analysis. Overall, ubiquitination, association and IF analyses should be quantified to support any conclusions stated in the manuscript. The number of independent repeats should be clearly stated. MW markers or sized should be added to the blots in this figure and elsewhere where appropriate.
7. To provide further evidence for ENTREP being a substrate for ITCH, experiments should be carried out using endogenous complement of the proteins, or at least with overexpressed TM-containing ENTREP. Same should be considered for experiments shown in Figure 3D with respect to CXCR4-ENTRP association and role of ITCH in promoting an interaction (again CXCR4 seems to increase with and ITCH-ENTREP co-expression). In Figure 3F, what is the evidence of ubiquitination signal in the CXCR4 IP due to CXCR4 and not a coiping protein like ENTREP? Immunoblots of CXCR4 of the IP and lysates should be included. In addition, the CXCR4 IPs should be probed with ENTREP and ITCH. MW markers should be indicated. It is not clear if proteasome inhibition was used here.
8. For the data shown in Figure 4, how many clones and where different gr used for the ENTREP KO using the crispr approach. CXCR4 protein abundance seems to be low in the KO, which is somewhat seems to be contradictory to the expected outcome (Figure 4A). Quantifying the protein abundance of CXCR4 in the KO obtained from independent experiments should be statistically analysed and presented to address the question. In this regard, has the effect of CXCL12 on CXCR4 in these lysates been tested, since CXXL13 appears to increase overall pixel numbers of CXCR4. Quantification of pixel numbers and intensity (as well as localization) and statistical analyses will also help in data interpretation. Rescue approach should include, in addition to wild type ENTREP, the ITCH-interaction mutant ENTREP to provide more support for the interplay between ITCH-ENTREP in the functional analysis shown in figures 4 and 5.

**Response to Reviewers**

Dear Reviewers,

Thank you very much for your interest in, thoughtful comments on, and invaluable suggestions to our study. We also thank you very much for the extended revision time you have allowed, which we indeed needed under the difficult condition of Covid-19 spreading.

We have carried out many experiments with your suggestions in mind and are now very excited to share these results with you. As you see in the point-to-point reply below, we have modified our interpretation about the ubiquitin linkage and EPN1-binding of ENTREP/FAM189A2. The new interpretation, we believe, more clearly highlights a unique role of ENTREP/FAM189A2 in ITCH-mediated CXCR4 desensitization.

We hope we have thoroughly addressed your comments in the revised manuscript.

**Overview of the Figures in the revised manuscript:**

Fig 1: not changed.

Fig 2: major changes include:

- new Fig 2C (ubiquitination of ENTREP), showing the results of nickel-pull down assay under the denaturing condition.
- new Fig 2D (EPN1-binding of ENTREP), indicating no enhancement of EPN1-binding by ubiquitination.

Fig 3: major changes include:

- new Fig 3F (ubiquitination of CXCR4), showing the results of nickel-pull down assay under the denaturing condition.

Fig 4: major changes include:

- new Fig 4C (expression of endogenous CXCR4), showing the expression of endogenous CXCR4 in lentivirally-transduced MCF-7cripsr cells.

Fig 5: major changes include:

- new Fig 5A (migration assay) as a dot-plot presentation.
- new Fig 5C, illustrating modified concept on a role of ENTREP in ITCH-mediated CXCR4 desensitization.

**Overview of the Expanded View Figures:**

Fig EV1: not changed.

Fig EV2: not changed.

Fig EV3: major changes are as below.

- new Fig EV3A (the immunofluorescence staining of transfected Cos7 cells), showing the localization of ITCH and ENTREP.
- new Fig EV3B (the proximity ligation assay), showing the images of PLA and its quantitative results.
- new Fig EV3C (the cycloheximide chase assay), showing a mild effect of ITCH on the stability of ENTREP protein.

Fig EV4: major changes are as below.

- new Fig EV4B and EV4C (the immunoprecipitation analyses), showing the ITCH WW domain responsible for the association with ENTREP and the carboxyl-terminal tail of CXCR4.

Fig EV5: major changes are as below.

- new Fig EV5A-C containing old Fig EV 3, EV4A and EV4B.

(Table EV1, we moved to Appendix section, because of the limited number of FIG EVs.)

**Overview of the Appendix Figures and Table in the revised manuscript:**

Appendix Fig S1: new in the revision.

- Appendix Fig S1A (the immunoblot), showing the expression of ENTREP protein in the human breast cancer cell lines.
- Appendix Fig S1B-C (the immunoblot of the exon 5-skipping transcript variant of ENTREP), showing the protein instability of the variant.

Appendix Fig S2: new in the revision.

- the schematic summary of the expression vectors used in this study.

Appendix Fig S3: new in the revision.

- the immunoblot analyses of lentivirally-transduced MCF-7cripsr cells.

Appendix Table S1: same to the original Table EV1.

**Point-to-point reply to the Reviewers:**

Black letters are the reviewers' comments; blue letters are our reply.

**Referee #1:**

This manuscript reports the observation that FAM189A2/ENTREP downregulation impacts the prognosis of breast cancer patients. It further identifies FAM189A2/ENTREP as an interactor of the ubiquitin ligase ITCH and the endocytic adaptor EPN1 and characterizes the interactions, localization, and functional interplay between these proteins, along with the chemokine receptor CXCR4, which, like ENTREP, is a substrate of ITCH.

The manuscript, while interesting, is rather awkwardly written. This is reflected in a number of grammatical and structural issues that need careful editing. Specifically, a lot of information that ought to be in the introduction is mentioned in the results section. In addition, I feel that the functional/cell-based claims made in this manuscript should be consolidated with orthogonal techniques. Furthermore, the conclusion that ITCH forms non-degradative, K63-linked chains on CXCR4 and EPN1 is not sufficiently back up by data. In sum, this manuscript requires considerable improvement in order to meet the rigorous standards of EMBO Rep.

**Response-** Thank you very much for your comments. As for a manuscript format, we appreciate your understanding that our manuscript was submitted to the *Report* section and it should have a combined *Results and Discussion*.

Specific comments:

Introduction:

- Overall, the introduction is rather short and not particularly informative. Apart from the last paragraph, which is a summary of the new findings, there are just a few rather general sentences leading to the question studied. I feel that the introduction needs to be significantly more detailed and provide a more specific expert introduction to ITCH/FAM189A2 and the cellular context studied.

**Response-** Thank you for your comments. We rewrote the introduction according your advice.

- The authors should rephrase the sentence stating that E3 ligases function at the "most critical step" of the ubiquitination cascade. Each step is necessary and thus critical. Maybe the authors intended to refer to specificity?
- The authors appear to state that U-box ligases only occur in Arabidopsis, which is incorrect.
- The authors should say that ligases are classified into 4 main types (rather than "4 types"), since they do not mention the recently discovered RCR ligase (of which it is yet unclear whether it is a class or a single member).

**Response-** We rephrased and fixed in the revision. Please see line 4, 7, 10 and 11 in page 3.

- When introducing the fact that HECT ligases can be allosterically autoinhibited, the authors should also Chen et al., Mol Cell, 2017 (for WWP2), Sander et al., eLife 2017 (for HUWE1), and Attali et al., EMBO J 2017 (for NEDD4).

**Response-** Thank you for your introduction. Please see line 9 in page 4.

Results:

- Some of the information that is missing in the introduction appears within the results section. This information should be moved. For example, on page 6, there is some useful information on the NEDD4-subfamily and ENP1/2 that should have been mentioned in the intro. Likewise, the paragraph at the end of page 8 (continuing on page 9) belongs into the introduction etc.

**Response-** Thank you for your suggestion. We fixed this. Please see line 16 in page 3 to line 5 in page 4.

- Figure 2C:

This experiment is performed under non-denaturing conditions. As such, it is not clear whether the observed smears result from co-IPed, ubiquitinated proteins or represent ubiquitinated forms of ENTREP. The authors need to state this - AND/OR - perform the experiment with His-tagged ubiquitin and IP under denaturing conditions (i.e. Nickel-pulldown, followed by Western against the substrate). Furthermore, in the current figure, the authors should show the total lysate FLAG-IB as a control.

The authors conclude that ubiquitination occurs mainly through K63. While plausible from the literature, this is not visible in their experiment, since there is a clear difference between the ubiquitin WT and K63-only lanes. From this experiment, one can only conclude that more ubiquitination occurs through K63 than K48. Obviously, there are either additional lysines involved in the modification and/or the mutation of 6 lysines at once in the K63-only and K48-only mutants, respectively, affects activity. These options should be investigated by using individual K/R-mutants.

**Response-** Thank you very much for your comments. We now understand that our original manuscript was immature in terms of examining the ubiquitination of ENTREP and CXCR4. In the revision, we carried out the nickel-pull down assay under the denaturing condition. As shown in new Fig 2C, the full length ENTREP harboring myc/HISx6-tag at the carboxyl terminus was transiently transfected along with

HA-tagged ubiquitin vectors in HEK293T cell. After MG132 treatment, the cell extracts were prepared in the guanidine-containing lysis buffer and served for the affinity purification with Ni-IDA resin. The resin was washed by the urea-containing washing buffer and then eluted in the imidazole-containing elution buffer. The elutants were used for the immunoblot analysis.

In the original manuscript, we used K48- or K63-ubiquitin vector to examine ENTREP ubiquitination, concluding that K63-linkage was major for ENTREP ubiquitination. But as a result of the nickel-pull down assay under the denaturing condition, we have now found that K27, 29 and 33-linked ubiquitination were much more prominent than K63-linked one. ITCH reportedly participates in these types of ubiquitination (Chastagner et al., 2006, Huang et al., 2010, Yin et al., 2019). Therefore, we have replaced the original Fig 2C with new Fig 2C. Please see line 22 in page 8 to line 2 in page 9. As for the experiments using K/R-mutants, which we thought crucial, we have not finished yet because we did not see the selectivity of ubiquitin linkage and might need a various K/R-mutants.

We would like to have your comments on the result of our pull-down assay.

Also, more experiments should be done to support the hypothesis that "Active ITCH ubiquitinates ENTREP primarily through Lys63-linked ubiquitination, which may protect ENTREP protein from degradation and further enhance its association with EPN1." (see discussion). Such experiments could include: cycloheximide-chase experiments to confirm that the ITCH-mediated ubiquitination of ENTREP is not degradative; and the above-mentioned experiments with Lys-to-Arg mutants of ubiquitin to demonstrate that the chains are indeed primarily linked via Lys63.

**Response-** We conducted the cycloheximide chase assay, the result of which indicated a mild stabilizing effect of ITCH on ENTREP protein (new Fig EV3C; line 17 to 21 in page 9). As for the EPN1 association, please see our response below.

- Figure 2 D:

The expression of Flag-ENTREP as well as myc-EPN1 is overall higher in +ITCH setup (see input). This may explain higher levels of EPN1 in myc-pulldown instead of ITCH having an effect. Therefore, I recommend to include catalytically dead ITCH to ensure that it is the ubiquitination of ENTREP that enhances binding.

Why is there a smear in the no-ITCH lane (FLAG IP, HA IB)? The authors should comment on the possibility of ubiquitination by endogenous ITCH or other E3s.

Also, marker positions should be indicated.

**Response-** Thank you for your comments. We found that ITCH-mediated ubiquitin linkage on ENTREP seemed to be multi-monoubiquitin rather than polyubiquitin (new Fig 2C; line 10 to 12 in page 9). EPN1 reportedly binds K63-linked polyubiquitin chain but it shows extremely poor affinity for monoubiquitin (Hawryluk et al., 2006). With your comments in mind, we carefully repeated the immunoprecipitation analyses using ITCH mutant. As shown in new Fig 2D, ITCH C830A did not obviously reduce the ENTREP co-precipitation of EPN1 and ENTREP mut1+2 mutant similarly co-precipitated in the presence of active ITCH. Based on these results, we modify our interpretation: the EPN1 association of ENTREP was not solely dependent on its ubiquitination (line 6 to 15 in page 9).

Importantly, when co-transfected with EPN1myc, the expression of ENTREP-FLAG was detected at the plasma membrane and in the cytoplasm (Fig 2F). And the proximity ligation assay revealed the ENTREP-EPN1 association at the plasma membrane and in the cytoplasm (Fig EV3B). co-transfection of Halo-ITCH enhanced ENTREP endocytosis, whereas it did not have a significant effect on the endocytosis of ENTREP mut1+2 mutant (Fig 2F). These evidences indicate that the endocytosis of ENTREP was primarily induced by the association with EPN1 and further enhanced by the association with ITCH.

We fixed protein size marker.

- In general, it would be helpful if the authors commented on their choice of cell lines. Why Cos7 cells on page 7 row 25? Why MCF-7 cells instead of HMEC on page 5 row 22.

**Response-** We used transfected Cos7 cells to analyze the subcellular localization of ENTREP, because their large cytoplasmic area makes the immunofluorescence observation easier.

When started this study, we simply expected a loss-of-function type mutation of *ENTREP* in MCF-7 cells, because *ENTREP* expression was downregulated in the other cancer cell lines. But the result of its sequencing signified to us that MCF-7, which reportedly shows a slow migration and has a weak potential of mammosphere formation, would be unique in the expression of wild type ENTREP.

- Figure 3C:

The input of Halo-ITCH is lower for delW14 and much lower for delW34, That CXCR4 favours WW34 is thus not convincingly confirmed. Thus, the authors should show equal levels or adjust their interpretation.

**Response-** In new Fig 3B and 3C, we presented the immunoblot of total lysate/Halo IB, which were longer exposer images of the original Fig 3B and 3C to show the presence of delWW34 protein in the inputs. We agree that the inputs of delWW34 were not same to those of the other delWWs. But in this condition, FLAG-ENTREP co-precipitated with delWW34 in Fig 3B, and delWW34, but not HA-CXCR4DD-DsRed, co-precipitated with FLAG-ENTREP in Fig 3D.

- Fig. 4 C:

I am not convinced that the amount of CXCR4 in the cytoplasm is "substantial"

**Response-** Thank you for your comments. Your concern is related with those mentioned by Reviewer #2 and #3, so we here introduce the experiments and their results shown in new Fig 4A, 4C and appendix Fig S3.

In the original Fig 4C, we transiently transfected MCF-7crispr cells with either ENTREP-EGFP or its control EGFP plasmid vector. But it was difficult for us to quantify the endogenous CXCR4 expression of these cells; more importantly, the expression level of endogenous CXCR4 differs from cell to cell. Therefore, we prepared lentivirus for the expression of ENTREP-EGFP, ENTREP mut1+2-EGFP and control EGFP, transduced them into MCF-7cripsr cells and served the cells for the immunoblot analyses (Appendix Fig S3) and the immunofluorescence staining (new Fig 4C). The immunoblot analyses revealed that the expression of ENTREP-EGFP, ENTREP mut1+2-EGFP or control EGFP did not affect the endogenous CXCR4 expression, whether with or without 1 hour treatment of CXCL12 (Appendix Fig S3). In this condition, we performed the immunofluorescence staining and examined the localization of CXCR4 under a laser confocal microscopy. As shown in new Fig 4C, a non-negligible amount of CXCR4 was observed in the cytoplasm of ENTREP-EGFP expressing cells after CXCL12 treatment (line 23 in page 13 to line 8 in page 14).

We realized that the cell morphology of lentivirally-transduced cells was slightly different from those of the cells transiently transfected with plasmid vectors: we currently assume the possibility that the exposure to Lipofectamine and/or the rapid and strong expression of proteins by plasmid vectors would affect the cell morphology.

- Figure 3 F:

Here, the same criticism applies as to 2C. The authors should transfect His-Ubi and perform a Nickel-pull down under denaturing conditions in order to separate ubiquitination of CXCR4 from potentially ubiquitinated interaction partners.

Further, the authors state that Fig 3F shows that "ITCH did not appreciably ubiquitinate

CXCR4" (page 10). I wonder whether this statement also holds for monoubiquitinated CXCR4, since the blot is cropped just below 50 kDa. If no, the authors should replace the word "ubiquitinate" by "polyubiquitinate"

**Response-** Thank you very much for your comments. As mentioned above, we carried out the nickel-pull down assay under the denaturing condition to analyze CXCR4 ubiquitination. First, we transfected HEK293T cells with the full length CXCR4-myc/HISx6 vector and HA-tagged ubiquitin vector along with either ITCH or ITCH plus ENTREP and served the cells for the nickel-pull down assay under the denaturing condition. However, we saw myc-positive signals, which were either positive or negative with HA, at the top of the gel and in the smear. These relate, in our view, to our failure to have a nice separation of these pull-downed samples in the SDSPAGE.

CXCR4 harbors seven-transmembrane domains and is thought to be polyubiquitinated by ITCH. So, we modified the assay by using HISx6-tagged ubiquitin along with HA-CXCR4DD-DsRed, which harbors the carboxyl-terminal tail of CXCR4 but not the transmembrane domain. As shown in new Fig 3F, we found that FLAG-ENTREP enhances the ITCH-mediated polyubiquitination of HA-CXCR4DD-DsRed (line 8 to 10 in page 13). In this experiment, we were not able to estimate the effect of CXCL12 treatment because of the lack of the extracellular ligand-binding domain in HA-CXCR4DD-DsRed.

- Figure 5C:

How can both ENTREP and ITCH occupy the same WW domain of ITCH (which they must since CXCR4 is co-precipitated in ENTREP-pulldowns through ITCH)? How do the authors think about this? Please elaborate on this in the discussion.

**Response-** Thank you for your comments. For the revision, we constructed ITCH delWW123 (deletion of WW1, WW2 and WW3) and delWW124 (deletion of WW1, WW2 and WW4) and examined which WW domain was responsible for the association. The immunoprecipitation analyses revealed that WW4 of ITCH was responsible for the association with ENTREP, whereas WW3 and WW4 seem to equally contribute the association with HA-CXCR4DD-DsRed (new Fig EV4B and EV4C). We mentioned this result in the revised manuscript (line 15 to 22 in page 12).

- In general, I feel that the IF data should be either quantified or confirmed by an orthogonal technique, such as proximity ligation, crosslinking mass spec etc..

**Response-** In the original manuscript, we have not examined whether the full length ENTREP associated with ITCH or EPN1 and we also did not show where the

association was detected in cells. We thus employed the proximity ligation assay (PLA) for the revision. When co-transfected with the full length ENTREP-FLAG, catalytic-dead ITCH C830A-Halo produced many PLA signals in the cell area including the plasma membrane: co-transfection of the PPxY-motif mutated full length ENTREP mut1+2-FLAG did not show a significant number of PLA signals, even though its expression seemed to be overlapped with that of ITCH C830A-Halo at the plasma membrane (new Fig EV3A and EV3B). These evidences indicated that the full length ENTREP associates with ITCH at the plasma membrane in the PPxY motif-dependent manner (line 12 to 22 in page 8). We also found the association between the full length ENTREP-FLAG and EPN1-Halo by the PLA (new Fig EV3B; line 5 to 6 in page 9). For the PLA, we used EPN1-Halo, instead of EPN1myc used in the immunoprecipitation analyses. We appreciate your understanding that we needed rabbit anti-tag antibody, instead of mouse anti-myc antibody, to detect EPN1 expression for the PLA (line 13 to 22 in page 34).

Discussion

There is no clear separation between the results and discussion part, which should be indicated.

**Response-** We appreciate your understanding that our manuscript was submitted to the *Report* section and it should have a combined *Results and Discussion*.

Methods

The authors should provide more detail on the position of the tags in the individual constructs. This is particularly necessary for the HECT constructs, since these ligases are known to be sensitive to C-terminal tagging.

**Response-** We apologize for the missing of vector information: in the revision, we attached the schematic summary of the expression vectors used in this study as Appendix Fig S2.

**Referee #2:**

In this study, Tsunoda et al. identify the previously uncharacterised protein FAM189A2 as a regulator of the HECT E3 ligase ITCH. In an earlier study the authors had identified FAM189A2 as a down regulated gene in several types of breast cancer (Riku et al., 2016).

In the current study, the authors propose that FAM189A2 recruits ITCH to the plasma membrane and enhances ITCH-mediated ubiquitination of the chemokine receptor CXCR4. Furthermore, ubiquitination of FAM189A2 by ITCH is suggested to enhance the association of FAM189A2 with EPN1, promoting endocytosis of the FAM189A2-ITCH-CXCR4 complex. As a result, loss of FAM189A2 function is linked to enhanced chemotaxis and stemness of MCF-7 breast cancer cells. In line with this model, the authors propose renaming FAM189A2 to Endosomal Transmembrane binding with EPsin (ENTREP).

While this study is conceptually interesting, we feel that part of the model is not sufficiently supported by the current experimental data presented, and thus suggest further basic experimentation to clarify key points.

Notably the authors describe ENTREP as an 'adaptor' of ITCH. In our view, the term adaptor implies that ENTREP mediates the interaction between two proteins, in this case ITCH and CXCR4 - by binding to both simultaneously. Instead the proposed model (Fig 5C) shows that ENTREP binds to and recruits ITCH to the membrane and activates ITCH - but does not bind to CXCR4. More appropriately, ENTREP might be considered an 'activator' or 'recruiter' of ITCH, although neither of these functions are explicitly demonstrated by the current data.

Major points

1) Based on the model, ITCH should be recruited to the plasma membrane in an ENTREP-dependent manner. Is this indeed the case in cells? The authors should be able to use their MCF-7crispr k/o cell line or the ITCH (delWW14) or ENTREP (mut1+mut2) mutants to investigate this point. These experiments may require the use of the ITCH active site mutant or an inhibitor to block downstream signalling and endocytosis.

**Response-** Thank you very much for your comments. We first examined the expression of endogenous ITCH in lentivirally-transduced MCF-7cripsr cells (Figure 1 for reviewer only; attached below). In the cells expressing either control EGFP or mut1+2 mutant, endogenous ITCH was not clearly overlapped with EGFP at the plasma membrane (*arrowheads*). And in the cells expressing ENTREP-EGFP, some of endogenous ITCH and EGFP was overlapped in the cytoplasm but a faint co-localization was seen at the plasma membrane. We thought this result reasonable: even though ENTREP-EGFP could recruit endogenous ITCH to the plasma membrane, the ITCH-bound ENTREP would be efficiently endocytosed (Fig 2F). Therefore, on the basis of these experiments, we did not reach the conclusion whether ENTREP recruits ITCH to the plasma membrane. We next employed overexpression system: we transiently transfected Cos7

cells with catalytic-dead ITCH C830A-Halo either with or without ENTREP-FLAG. As shown in Fig EV3A, we observed Halo signal at the plasma membrane of the cells without ENTREP co-transfection; this membranous expression pattern of Halo was same to those of the cells co-transfected with either ENTREP-FLAG or PPxY-mutated ENTREP-mut1+2-FLAG, though mut1+2 mutant did not produce a significant number of signals at the plasma membrane in the proximity ligation assay (Fig EV3B). These results indicated that in this overexpression system ITCH relocates, in an ENTREP-independent manner, to the plasma membrane and there meets and associates with ENTREP in the PPxY-motif-dependent manner (line 12 to 22 in page 8). Based on these results, we modified Fig 5C to illustrate the ENTREP-independent relocation of ITCH to the plasma membrane.

With your comments in mind as well as these results, we agree with you on rewording *adaptor*. We change *adaptor* to *activator* in the title and the text of the revised manuscript.

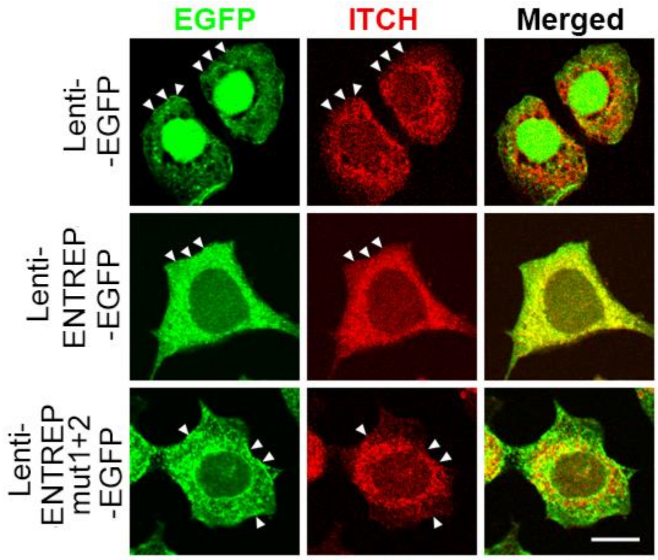


**Figure 1 for reviewer's attention. Endogenous ITCH expression in lentivirally-transduced MCF-7cripsr cells.** After fixation and permeabilization, the cells were stained with anti-ITCH antibody (D8Q6D; Cell signaling Technology), followed by the second antibody staining. *Scale bar*, 10μm.

2) Many of the experiments imply that the observed effects and ubiquitination are mediated by ITCH E3 ligase activity. However, this could potentially also be mediated by other E3 ligases in the cell, or by a scaffolding function of ITCH. The authors should

include the ITCH C830A active site mutant in all of the relevant experiments (Figs. 2C, 2D, 2E, 3F) to exclude these possibilities.

**Response-** To verify ubiquitination of ENTREP and CXCR4, we carried out the nickel-pull down assay under the denaturing condition in the revision (response to Referee #1). Briefly, the full length ENTREP harboring myc/HISx6-tag at the carboxyl terminus was transiently transfected along with HA-tagged ubiquitin vectors in HEK293T cell. After MG132 treatment, the cell extracts were prepared in the guanidine-containing lysis buffer and served for the affinity purification with Ni-IDA resin. The resin was washed by the urea-containing washing buffer and then eluted in the imidazole-containing elution buffer. The elutants were used for the immunoblot analysis. As for CXCR4 ubiquitination, we used HA-CXCR4DD-DsRed, instead of CXCR4-myc, along with HISx6-ubiquitin vector for the nickel-pull down assay under the denaturing condition.

As for the original Fig 2C, we changed to new Fig 2C: ITCH C830A did not ubiquitinate ENTREP (line 22 in page 8 to line 2 in page 9).

As for the original Fig 3F, we changed to new Fig 3F: ITCH C830A did not contribute to the ENTREP-mediated enhancement of HA-CXCR4DD-DsRed ubiquitination. We saw a weak ubiquitination signal of HA-DsRed (at the first lane) and HA-CXCR4DD-DsRed (at the last lane): we suppose that the endogenous activity of other E3 ligases might contribute to these background effects (line 8 to 10 in page 13).

As for the original Fig 2D, we changed to new Fig 2D and we have to modify our interpretation of the association between ENTREP and EPN1. As mentioned above (response to Referee #1), ITCH-mediated ubiquitin linkage on ENTREP seemed to be multi-monoubiquitin rather than polyubiquitin (new Fig 2C; line 10 to 12 in page 9). EPN1 reportedly shows extremely poor affinity for monoubiquitin (Hawryluk et al., 2006). Consistently, ITCH C830A did not obviously reduce the ENTREP co-precipitation of EPN1 and ENTREP mut1+2 mutant similarly co-precipitated in the presence of active ITCH (new Fig 2D). Based on these results, we now think that the EPN1 association of ENTREP was not solely dependent on its ubiquitination (line 6 to 15 in page 9).

As for the original Fig 2E, we changed to new Fig 2F: there we employed the PPxY-motif mutant ENTREP mut1+2-FLAG, instead of ITCH C830A, because we examined in this experiment whether ITCH-enhanced endocytosis of ENTREP was dependent on its association with ITCH (line 5 to 17 in page 10).

3) The study mainly relies on overexpression experiments using an ENTREP construct that only contains the cytoplasmic region of the protein. This artificial system may

heavily affect localisation and function of ENTREP, a transmembrane protein in nature.

**Response-** Thank you for your comments. With your comments in mind, we conducted the proximity ligation assay (PLA) for the revision to verify the association of the full length ENTREP in the cells. The PLA revealed that co-transfection of the full length ENTREP-FLAG along with catalytic-dead ITCH C830A-Halo produced many PLA signals in the cell area including the plasma membrane, whereas co-transfection of the PPxY-motif mutant ENTREP mut1+2-FLAG did not produce a significant number of PLA signals, even though the expression of the mut1+2 mutant was overlapped with that of ITCH C830A-Halo at the plasma membrane (new Fig EV3A and EV3B). These evidences indicated the association of full length ENTREP and ITCH at the plasma membrane in the PPxY motif-dependent manner (line 12 to 22 in page 8). Similarly, we also found the association of full length ENTREP-FLAG and EPN1-Halo (new Fig EV3B; line 5 to 6 in page 9).

4) The authors show that ITCH, ENTREP, EPN1 and CXCR4 form a stable complex. For example, they are able to pull-down Halo-ITCH and CXCR4 with an anti-FLAG antibody directed against FLAG-ENTREP (Fig 3D). This implies that all the pull-downs contain a mixture of these and potentially other proteins. In the ubiquitination experiments (Figs. 2C, 2D and 3F), the authors only blot for HA-Ub but not for the proposed substrate. The authors should include blots showing molecular weight shift of these substrates.

**Response-** Thank you very much for your comments. First of all, we seriously took concerns for the ubiquitination of ENTREP and CXCR4. To verify these, we conducted in the revision the nickel-pull down assay under the denaturing condition (new Fig 2C and new Fig 3F). This experimental condition using the guanidine or urea-containing buffer aimed to disrupt a protein association and to simply examine whether ENTREP or CXCR4 would be really ubiquitinated: we successfully demonstrated that ITCH ubiquitinated ENTREP (new Fig 2C) and that ENTREP enhanced ITCH-mediated ubiquitination of HA-CXCR4DD-DsRed (new Fig 3F). In these experiments, unfortunately, we were not able to demonstrate the presence of the protein complex.

As for new Fig 2D, we have not examined the ENTREP-ITCH-EPN1 complex. When preparing the original manuscript, we thought that ITCH-mediated ubiquitination would enhance the ENTREP association with EPN1, because EPN1 harbors the ubiquitin-interacting motif (UIM) responsible for binding with various ubiquitinated proteins. But based on the results of carefully-conducted immunoprecipitation for the revision, we realized that the ubiquitination of ENTREP did not significantly participate in

its association with EPN1 (new Fig 2D), indicating the possibility that an undetermined region, rather than the UIM, of EPN1 would be responsible for the association with ENTREP (line 6 to 15 in page 9). As shown in new Fig 2D, we would need the deletion mutant(s) of EPN1, which lacks the responsible region for binding with ENTREP, in order to demonstrate an ENTREP-ITCH-EPN1 ternary complex. We agree with you that the immunoprecipitation using such a deletion EPN1 mutant(s) would be important in the future direction of research.

Minor points

1) ITCH is known to form K29, 48 and K63 ubiquitin chains. However, the authors only test for K48 and K63 chains in Fig 2C. They should include a K29 only ubiquitin mutant.

**Response-** Thank you very much for your comments. As mentioned above, we carried out the nickel-pull down assay under the denaturing condition, and in the assay we employed HA-tagged K0, K27, K29, K33 ubiquitin vectors (new Fig 2C). As a result, we concluded that K27, 29 and 33-linked ubiquitination were much more prominent than K63. ITCH reportedly participates in these types of ubiquitination (Chastagner et al., 2006, Huang et al., 2010, Yin et al., 2019). In the revision, we mentioned about these results (line 22 in page 8 to line 2 in page 9; line 15 to 21 in page 9).

2) Fig 2D lane 3 (FLAG-ENTREP/HALO/EPN1myc/HA-ubiquitin) shows a HA-ubiquitin smear indicating E3 ligase activity. This is different to Fig 2C lane 3, which almost shows the same conditions; the only difference being absence of EPN1myc in Fig2C. Is this a real effect potentially mediated by EPN1 or an experimental artifact?

**Response-** As mentioned above, through the nickel-pull down assay under the denaturing condition (new Fig 2C), the PLA (new Fig EV3B), the immunofluorescence staining (new Fig 3F) as well as a carefully-conducted immunoprecipitation assay using ITCH C830A (new Fig 2D), we have to modify our interpretation: the EPN1 association of ENTREP was not solely dependent on its ubiquitination. We mentioned these results in line 3 to 15 in page 9. As for a HA-ubiquitin smear in the original Fig 2D, we appreciate your understanding that we are currently not able to distinguish a real effect from an experimental artifact.

3) The colocalization in Fig 2E is not very convincing as the overexpressed proteins are present in the entire cell. Also, in light of our major point 3 above, can the authors visualise endogenous protein or use a lower level of overexpression? It is also unclear where the insets have been taken from. This should be indicated.

**Response-** We transfered the original Fig 2E as new Fig 2F of the revised manuscript. Because of limitation of antibody available for the immunofluorescence staining, we failed to demonstrate the co-localization of endogenous ENTREP, EPN1 and ITCH in the cells. To examine whether the co-localization really indicates the association, we conducted the PLA as mentioned above.

In new Fig 2E and 2F, we attached the indication for the insets.

4) Fig 3C: The HALO-ITCH expression levels vary heavily between the different mutants and seem to correlate with the amount of protein pulled down. This experiment should be repeated with more consistent expression levels. Fig 3D shows that more comparable expression of these constructs can be achieved.

**Response-** As mentioned above (response to referee #1), we present new Fig 3B and 3C, which were longer exposer images of the original Fig 3B and 3C to show the presence of delWW34 protein in the inputs.

We agree that the inputs of delWW34 were not same to those of the other delWWs. But in this condition, FLAG-ENTREP co-precipitated with delWW34 in Fig 3B, and delWW34, but not HA-CXCR4DD-DsRed, co-precipitated with FLAG-ENTREP in Fig 3D.

5) Fig 3E: The authors imply that increased ENTREP expression leads to increased ENTREP binding to ITCH, which in turn leads to increased CXCR4 recruitment. While the latter is evident from this figure, the authors should also blot for FLAG-ENTREP to show the first point.

**Response-** We include Halo IP/ FLAG IB in new Fig 3E, showing a weak increase of FLAG-ENTREP co-precipitation.

6) Fig 4C: How does the GFP-ENTREP overexpression compare to the endogenous ENTREP expression levels in wild-type cells?

**Response-** In the original Fig 4C, we tried to rescue MCF-7crispr cells by the transfection of ENTREP-expressing plasmid vector. We know that it was probably much higher than endogenous ENTREP level of parental MCF-7 cells: therefore, a function-dead control, ENTREP mut1+2-EGFP, should be required for a proper estimation of such overexpression experiments. In new Fig 4C, we present the images of lentivirally-transduced cells, in which the expression level of ENTREP might be much more than that of parental MCF-7 cells (data not shown). But we employed either EGFP or ENTREP mut1+2-EGFP as a control of ENTREP-EGFP and the overexpression of

any of wild type, mut1+2 mutant, or EGFP alone did not affect the expression level of endogenous CXCR4 (Appendix Fig S3).

7) Fig 5A: Individual data points should be included in these graphs.

**Response-** We changed the original Fig 5A to new Fig 5A as a dot-plot presentation.

8) Materials and methods for the pull downs and Western blots need to be included.

**Response-** We mentioned in the Materials and Methods section of the revision (line 6 to 20 in page 20).

9) Most of the blots do not contain MW markers. These need to be added.

**Response-** We fixed this.

10) The blots are heavily cropped and full blots should be shown in a supplement.

11) The HALO blots are often split to show HALO-ITCH in one strip and HALO alone in another strip. The authors should consider showing this in a single frame. This is particularly confusing in Fig 3D, where the 2nd FLAG IP/Halo IB strip is completely empty.

**Response-** In new Fig 3D, we present the original, uncropped images of the immunoblot: we did the immunoblot analysis (total lysate and FLAG IP) using anti-Halo antibody, followed by anti-HA antibody on the same membrane. Therefore, new Fig 3D contains Halo-positive bands and HA-positive bands.

12) The error bars in Fig 1A are not defined.

**Response-** These SD were not so big: the error bars were attached in Fig 1A.

13) Typos:

a. Fig 2C: 'ENP1myc' should be 'EPN1myc'

b. Page 10: "NDFIP1 binds the WW domain of ITCH, opens its intermolecular structure" - should read "intramolecular"

**Response-** Thank you for your comments. We fixed these.

**Referee #3:**

In this manuscript, its is suggested that the gene product FAM189A2 (termed here as

ENTREP) associate with the C2-WW-HECT-containing E3 ubiquitin ligase ITCH and the endocytic-marker epsin1/2 (EPIN) with the overall effect to promote the ubiquitination, vesicular trafficking and degradation of the ITCH substrate CXCR4. Functionally, ENTREP is suggested to promote the ability of ITCH to downregulate the ability of CXCL12-CXCR4 signalling axis to promote chemotaxis and anchorage-independent cell growth of breast cancer cells. To provide support of the suggested conclusions, additional controls and experiments are required.

Specific comments:

1. Figure 1A, mRNA? Protein level? MCF7 higher mRNA level, is that reflected at the protein level? The molecular subtypes of all cancer cell lines should be included in the manuscript. What is known about itch and CXCR4 expression in these cell lines.

**Response-** Fig 1A shows the mRNA expression as mentioned in the figure legend.

We attach preliminary data for the expression of *CXCR4* and *ITCH* mRNA as below (Figure 2 for reviewer only). When compared with immortalized MEC4tertshp16 cells, *CXCR4* and *ITCH* mRNA expression were not so high in MCF-7 cells. ENTREP transduction into BT474 would probably produce obvious changes rather than MCF-7: however, BT474 is a luminal-B type breast cancer cell line and the Kaplan-Meier plotter did not predict a significant impact of *ENTREP* expression on the long-term prognosis of luminal-B patients. Therefore, in this study, we employed MCF-7 cells, which is a luminal-A subtype cell line highly expressing endogenous ENTREP.

We add the information of human breast cancer cell lines in the Materials and Methods (line 6 to 9 in page 18).

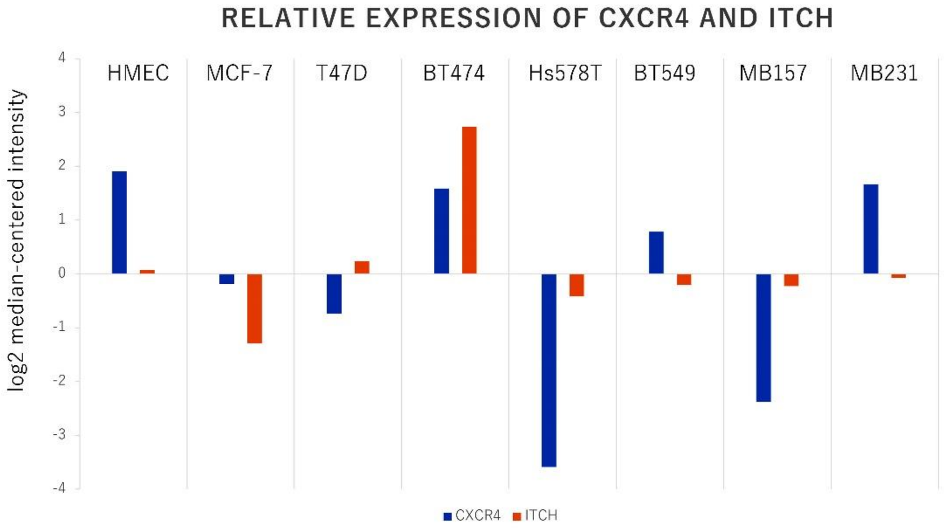


**Figure 2 for reviewer's attention. mRNA expression of *CXCR4* and *ITCH*.** The

mRNA expression of *CXCR4* and *ITCH* in the primary human mammary epithelium HMEC and human breast cancer cell lines. The mRNA expression was normalized to the immortalized normal human mammary epithelium HMEC4tertshp16. *Bar* indicates a mean from three independent samples.

2. The implication of ENTREP correlation with RFS but not with overall survival should be alluded to in the discussion. The term "intrinsic subtypes" in paragraph 1 of page 5 should be defined/descried

**Response-** We mentioned our speculation (line 23 in page 14 to line 2 in page 15). And we add a brief explain for the intrinsic subtypes (line 1 to 3 in page 6).

3. Is the exon 5-less ENTREP isoform expressed at the protein level? if so, does it differ in function from TM-containing version? At least this point should be a point of speculation in the discussion. In this regard, is this the isoform used in the biochemical interaction studies?

**Response-** We present the immunoblot analysis in Appendix Fig S1C, showing the exon 5-skipping variant less stable than ENTREP/FAM189A2. We mentioned this result and the reason why we focused on ENTREP/FAM189A2 but not the exon 5-skipping variant (line 4 to 10 in page 7).

4. The yeast two-hybrid screen identified many interactors for the cytoplasmic version of ENTREP which was used as the bait. A rationale should be provided for the focus on testing the hits ITCH and EPIN1/2 for further analyses.

**Response-** We mentioned this in line 18 to 24 in page 7.

5. To confirm yeast two hybrid-results, coips were performed on cells overexpressing the proteins being investigated. It is not clear why the TM-containing ENTREP protein is not used in these coips. In terms of WW-domain-dependent interactions, are each or both of WW1 and 4 required equivalently for such interactions? Overall, it would be important to investigate/confirm ENTREP-ITCF-EPN1 association at endogenous level of these proteins.

**Response-** In the original submission, we did not examine the association between the full length ENTREP and ITCH or EPN1 by the immunoprecipitation analyses: in the revision, we employed the proximity ligation assay (PLA) using the full length ENTREP-FLAG and catalytic-dead ITCH C830A-Halo or EPN1-Halo in Cos7 cells (new Fig EV3B). When co-transfected with the full length ENTREP-FLAG, ITCH C830A-Halo

produced many PLA signals in the cell area including the plasma membrane. And co-transfection of the PPxY-motif mutated full length ENTREP mut1+2-FLAG did not show a significant number of PLA signals, even though its expression seemed to be overlapped with that of ITCH C830A-Halo at the plasma membrane (new Fig EV3A and EV3B). These results indicated the PPxY motif-dependent association between full length ENTREP and ITCH at the plasma membrane (line 12 to 22 in page 8). Similarly, we confirmed the association of full length ENTREP-FLAG and EPN1-Halo by the PLA (new Fig EV3B; line 5 to 6 in page 9).

As for the ITCH WW domain responsible for the ENTREP association, we constructed ITCH delWW123 (deletion of WW1, WW2 and WW3) and delWW124 (deletion of WW1, WW2 and WW4) and detailed which WW domain was responsible for the association. The immunoprecipitation analyses using these mutants revealed that the WW4 of ITCH was responsible for the association with ENTREP, whereas either WW3 or WW4 seems to contribute to the association with HA-CXCR4DD-DsRed (new Fig EV4B and EV4C). We mentioned this result in the revised manuscript (line 15 to 22 in page 12).

We failed to co-precipitate the endogenous complex of ENTREP-ITCH-EPN1 from the cells: the co-precipitation of ITCH and EPN1 along with transmembrane-spanning ENTREP protein was technically difficult.

6. The Lysine residues and the nature of the mutation for the HA-ubiquitin should be clearly stated in figure 2C. Inclusion of a ubiquitin with all lysine residues converted arginine to prevent poly-ubiquitination of a substrate would be recommended as a negative control in figure 2C. Data showing the ubiquitination assays in which cells are not treated with MG132 will help reveal the importance of ubiquitination for ENTREP protein stability. In this regard, it is not clear how ENTREP stabilization by ITCH was assessed. In figure 2C, it appears that Ub(K48) to be involved in polyubiquitination of ENTREP. In addition, K63 does not appear to be sufficient to generate the same degree of poly-ubiquitination as that of wild type ubiquitin. These data and implication should be discussed. Overall, a FLAG immunoblotting of the ENTREP immunocomplexes (anti-FLAG IP) should be shown and used in the normalization of the ubiquitinated species to that of unmodified ENTREP for each FLAG- immunoprecipitated lysate sample. Relating to figure 2D, as ITCH1 expression seems to increase the protein level of EPN1 and ENTREP, is the apparent increase in the ENTRP-EPN1 association under such condition largely due to an increase in their abundance? An ITCH-interaction mutant ENTREP (YA) should be tested for ability to coip EPN1, in the presence and

absence of ITCH, to provide further evidence for the conclusion that ITCH-mediated ubiquitination of ENTREP promotes its association with EPN1. Inclusion of ITCH and ENTREP without EPN1 should be considered as a control, especially for assessing the HA/ubiquitination signal of the FLAG IP. Results of the immunofluorescence experiments and suggested colocalization/trafficking presented in figures 2E and 2F are hard to ascertain an should be subjected to quantitative analysis. Overall, ubiquitination, association and IF analyses should be quantified to support any conclusions stated in the manuscript. The number of independent repeats should be clearly stated. MW markers or sized should be added to the blots in this figure and elsewhere where appropriate.

**Response-** Thank you very much for your comments. Based on a serious concern for the ENTREP ubiquitination (response to referee#1), we conducted the nickel-pull down assay under the denaturing condition (new Fig 2C). Briefly, the full length ENTREP harboring myc/HISx6-tag at the carboxyl terminus was transiently transfected along with HA-tagged ubiquitin vectors (wild, K27, K29, K33, K48, K63 and K0 in which all lysine residues were mutated) in HEK293T cell. After 3 hours treatment with MG132 (mentioned in the figure legend), the cell extracts were prepared in the guanidine-containing lysis buffer and served them for the affinity purification with Ni-IDA resin. The resin was washed by the urea-containing washing buffer and then eluted in the imidazole-containing elution buffer. The elutants were analyzed by the immunoblot analysis. This experimental condition successfully demonstrated the ITCH-mediated ubiquitination of ENTREP (new Fig 2C). However, as the result of the nickel-pull down assay mentioned above, we now realize that K27, 29 and 33-linked ubiquitination was much more prominent than K63. ITCH reportedly participates in these types of ubiquitination (Chastagner et al., 2006, Huang et al., 2010, Yin et al., 2019). In the revision, we therefore changed the original Fig 2C to new Fig 2C and mentioned this (line 22 in page 8 to line 2 in page 9). And we also found that ITCH-mediated ubiquitin linkage on ENTREP seemed to be multi-monoubiquitin rather than polyubiquitin (new Fig 2C): we detected HA-positive band in the K0 ubiquitin-transfected sample, indicating mono-ubiquitination on ENTREP. The K27, 29 and 33-ubiquitin transfected samples produced ladder-like HA-positive bands, indicating multi-monoubiquitin rather than polyubiquitin. We also conducted the cycloheximide chase assay, the result of which indicated a mild stabilizing effect of ITCH on ENTREP protein (new Fig EV3C; line 15 to 21 in page 9).

As mentioned above, we found that ITCH-mediated ubiquitin linkage on ENTREP seemed to be multi-monoubiquitin rather than polyubiquitin (new Fig 2C). And

EPN1 reportedly binds K63-linked polyubiquitin chain but it shows extremely poor affinity for monoubiquitin (Hawryluk et al., 2006). So, in the revision, we carefully repeated the immunoprecipitation analyses using ITCH mutant. As shown in new Fig 2D, ITCH C830A did not obviously reduce the ENTREP co-precipitation of EPN1 and ENTREP mut1+2 mutant similarly co-precipitated in the presence of active ITCH. Based on these results, we modified our interpretation: the EPN1 association of ENTREP was not solely dependent on its ubiquitination (line 13 to 15 in page 9).

Importantly, when co-transfected with EPN1myc, the expression of ENTREP-FLAG was detected at the plasma membrane and in the cytoplasm (Fig 2F). And the PLA also revealed the association of ENTREP and EPN1 at the plasma membrane and in the cytoplasm (Fig EV3B). However, co-transfection of Halo-ITCH induced ENTREP endocytosis more efficiently, whereas it did not enhance the endocytosis of ENTREP mut1+2 mutant (Fig 2F). These evidences indicate the endocytosis of ENTREP was induced by the association with EPN1 and further enhanced by the association with ITCH. These experiments were repeated at least three times and we found the results were consistent. We added protein marker on the blot images in the revision.

7. To provide further evidence for ENTREP being a substrate for ITCH, experiments should be carried out using endogenous complement of the proteins, or at least with overexpressed TM-containing ENTREP. Same should be considered for experiments shown in Figure 3D with respect to CXCR4-ENTRP association and role of ITCH in promoting an interaction (again CXCR4 seems to increase with and ITCH-ENTREP co-expression). In Figure 3F, what is the evidence of ubiquitination signal in the CXCR4 IP due to CXCR4 and not a coiping protein like ENTREP? Immunoblots of CXCR4 of the IP and lysates should be included. In addition, the CXCR4 IPs should be probed with ENTREP and ITCH. MW markers should be indicated. It is not clear if proteasome inhibition was used here.

**Response-** As mentioned above, we failed to co-precipitate the endogenous complex of either ENTREP-ITCH or ENTREP-EPN1 from the cells: the co-precipitation of these along with transmembrane-spanning ENTREP protein was technically difficult. Instead, we employed the proximity ligation assay as mentioned above: the PLA revealed the association of the full length ENTREP with either catalytic-dead ITCH C830A-Halo or EPN1-Halo in Cos7 cells (new Fig EV3B; line 12 to 22 in page 8).

For the revision, as mentioned above (referee #1), we carried out the nickel-pull down assay under the denaturing condition in order to examine CXCR4

ubiquitination. First, we transfected HEK293T cells with the full length CXCR4-myc/HISx6 vector and HA-tagged ubiquitin vector along with either ITCH or ITCH plus ENTREP and served the cells for the nickel-pull down assay under the denaturing condition. However, we saw myc-positive signals, which were either positive or negative with HA, at the top of the gel and in the smear. These relate, in our view, to our failure to have a nice separation of these pull-downed samples in the SDSPAGE. CXCR4 harbors seven-transmembrane domains and was thought to be polyubiquitinated by ITCH. Therefore, we modified the assay by using HISx6-tagged ubiquitin along with HA-CXCR4DD-DsRed harboring the carboxyl-terminal tail of CXCR4 but not the transmembrane domain. As shown in new Fig 3F, we found that ENTREP enhanced the ITCH-mediated polyubiquitination of HA-CXCR4DD-DsRed. In this experiment, we were not able to examine whether CXCL12-induced ubiquitination of CXCR4 was enhanced by ENTREP, so we rephased as follows: ENTREP induced ITCH-mediated polyubiquitination of HA-CXCR4DD-DsRed (line 8 to 10 in page 13). The protein marker was added in the revised figures and usage of MG132 was mentioned in the figure legends.

8. For the data shown in Figure 4, how many clones and where different gr used for the ENTREP KO using the crispr approach. CXCR4 protein abundance seems to be low in the KO, which is somewhat seems to be contradictory to the expected outcome (Figure 4A). Quantifying the protein abundance of CXCR4 in the KO obtained from independent experiments should be statistically analysed and presented to address the question. In this regard, has the effect of CXCL12 on CXCR4 in these lysates been tested, since CXXL13 appears to increase overall pixel numbers of CXCR4. Quantification of pixel numbers and intensity (as well as localization) and statistical analyses will also help in data interpretation. Rescue approach should include, in addition to wild type ENTREP, the ITCH-interaction mutant ENTREP to provide more support for the interplay between ITCH-ENTREP in the functional analysis shown in figures 4 and 5.

**Response-** We generated knockout clones by using one type of guide DNA as mentioned in the Materials and Methods line 12 to 20 in page 18). We prepared four independent lysates from MCF-7cripsr and its parental MCF-7 cells respectively and examined the expression of CXCR4 and GADPH as mentioned in the figure legend. We found that the expression ratio of CXCR4/GAPDH was not significantly different between knockout cells and parental cells (new Fig 4A; line 14 to 22 in page 32). In the original Fig 4C, we used knockout cells transiently transfected with either ENTREP-EGFP or its control plasmid vectors. But it was difficult for us to quantify the

expression of endogenous CXCR4 and more importantly, the expression level of endogenous CXCR4 differs from cell to cell. Therefore, we prepared lentivirus for the expression of ENTREP-EGFP, ENTREP mut1+2-EGFP and control EGFP. We transduced these into MCF-7cripsr cells and served transduced cells for the immunoblot analyses (Appendix Fig S3) and the immunofluorescence staining (new Fig 4C). The immunoblot analyses revealed that none of these lentiviral transductions changed in the expression of endogenous CXCR4, whether with or without 1 hour treatment of CXCL12 (Appendix Fig S3). In this condition, we performed the immunofluorescence staining and examined CXCR4 localization under a laser confocal microscopy (new Fig 4C). As shown in new Fig 4C, a not-negligible amount of CXCR4 was observed in the cytoplasm of ENTREP-EGFP expressing cells after CXCL12 treatment. We realized that the morphology was slightly different between cells transiently transfected with plasmid vectors (original Fig 4C) and those stably transduced by lentivirus (new Fig 4C). We currently assume the possibility that the exposure to Lipofectamine and the rapid and strong expression of proteins by plasmid vectors would affect the cell morphology.

Dear Prof. Kasai,

Thank you for submitting your preliminary point-by-point response to the remaining referee concerns. I have now looked at your points carefully. I appreciate that you can address the outstanding concerns raised and see that the proposed experiments will strengthen the manuscript. We have thus decided to give you another chance to revise the manuscript.

However, I would like to point out that the next decision will be final, and that we need strong support from the referees to consider publication here.

I look forward to seeing a revised version of your manuscript when it is ready. Please let me know if you have questions or comments regarding the revision.

Kind regards,

Deniz Senyilmaz Tiebe

Deniz Senyilmaz Tiebe, PhD
Editor
EMBO Reports

Referee #1:

The authors have performed extensive revisions. Unfortunately, however, not all points were addressed adequately. In my eyes, the manuscript would therefore need considerable additional work:

For example, the authors have performed a pull-down experiment under denaturing conditions (new Figure 2C), as I had requested. In contrast to their previous data, this experiment shows increased substrate ubiquitination in the presence of the K27/29/33 single-Lys-mutants of ubiquitin. However, it is unclear whether the observed higher-order bands are due to ubiquitin chain formation or multi-mono-ubiquitination. Furthermore, several features of the new experiment are odd:
- Why do the K27/29/33 mutants give rise to more substrate ubiquitination than the WT?
- Why do the K48- and K63-only mutants show a loss of mono-ubiquitination of the substrate, while the K0-mutant can still monoubiquitinate?
If these effects are real, they should be quantified based on at least three experimental replicates. Furthermore, seeing a stimulation of activity towards ubiquitin mutants in which 6 residues have been altered (K27-only, K29-only, K33-only) strongly corroborates my previous request that this experiment should be performed with single Lys-to-Arg (K6R, K11R, K27R etc) mutants. In order to come to a meaningful conclusion, such experiments must be conducted. Based on the result, the authors should also interrogate the existence of chains (rather than multi-monoubiquitination on the substrate, e.g. using linkage-specific antibodies.
Right now, the text refers to "Lysine-27, 29 and 33-linked multi-monoubiquitin", which makes no sense.
I want to emphasize that the requested experiments are standard in the field (while even more advanced methods, such as quantitative MS are routineously employed), and the reagents readily available. I am therefore not satisfied with the statement "As for the experiments using K/R-mutants, which we thought crucial, we have not finished yet because we did not see the selectivity of ubiquitin linkage and might need a various K/R-mutants"
Sorting out the type of ubiquitin modification that is formed on ENTREP is also relevant for the interpretation of the consecutive experiments (e.g. the cycloheximide chase assay and effecs on ENTREP stability).

Figure 2D:
I am worried about the fact that the authors obtain a different result upon repeating their experiment than in the previous version of the manuscript. No explanation is provided for this, except "With your comments in mind, we carefully repeated the immunoprecipitation analyses using ITCH mutant. As shown in new Fig 2D, ITCH C830A did not obviously reduce the ENTREP co-precipitation of EPN1 and ENTREP mut1+2 mutant similarly co-precipitated in the presence of active ITCH. Based on these results, we modify our interpretation".
Under these circumstances, I can only reiterate my advice that the authors should repeat their experiment three times and report a quantification with error bars. This has also been requested by Reviewer 3 and should be done in all blots/IFs, based on which quantitative conclusions are drawn.

Referee #2:

During the revision of their manuscript now titled "ENTREP/FAM189A2 downregulated in breast cancer encodes a new activator for ITCH ubiquitin ligase to regulate ubiquitination and endocytosis of CXCR4", Tsunoda et al. addressed most, but not all, of

our comments and suggestions. The authors added significant amounts of new data, and especially the pull-down experiments under denaturing conditions, the PLA experiments and addition of the catalytically-dead ITCH C830A mutant, significantly strengthen or refine some of the conclusions of the initial manuscript.

Based on the revised data, there is a strong case that ITCH interacts with ENTREP via an WW1-4/PPxY motif interaction and that ITCH directly ubiquitinates ENTREP. The interaction between ENTREP and ITCH furthermore increases ubiquitination of the ITCH substrate CXCR4. ENTREP also interacts with EPN1 in an ITCH/ubiquitin-independent manner and facilitates EPN1 and ITCH endocytosis and thus controls CXCR4 downstream signalling.

Some of the newly added data does not support their initial model, which they now adjusted accordingly. In part because of these changes to the manuscript, parts of it are now quite difficult to follow and several open issues/concerns remain as detailed below.

1) Page 9 line 15: "EPN1 association of ENTREP was not solely dependent on its ubiquitination". What do the authors mean by this term? Is it dependent on ubiquitination or not? Based on the data it seems that ENTREP ubiquitination by ITCH has no effect on its interaction with EPN1. Thus, the only conclusion the authors can draw is that EPN1 association of ENTREP was not dependent on ITCH-mediated ubiquitination. Still in their final model figure, ENTREP ubiquitination by ITCH is prominently featured and it looks like this would recruit EPN1 as EPN1 is drawn in close proximity to one of the ubiquitin molecules. The model should be adjusted to reflect that ITCH-mediated ubiquitination on ENTREP does not affect EPN1 recruitment.
2) In the new manuscript the authors describe that ITCH forms "Lysine-27, 29 and 33-linked multi-monoubiquitin rather than polyubiquitin" on ENTREP. How do the authors derive this conclusion? And what do the authors mean by "Lysine-27, 29 and 33-linked multi-monoubiquitin"? A monoubiquitin on a substrate does not utilise any of the ubiquitin lysine residues. Lysine-27 etc. become only relevant in polyubiquitin chains (di-ubiquitin at the minimum). Fig 2C shows significantly less ENTREP ubiquitination with K0 ubiquitin and mostly mono-ubiquitination (top blot) suggesting that short poly-ubiquitin chains (mostly linked via K27, K29 or K33) are formed on ENTREP by ITCH. This needs to be clarified in the manuscript.
3) The authors should consider renaming their ENTREP KO cell line throughout the manuscript. MCF-7cripsr is not a good descriptor.
4) The K27, K29 and K33 ubiquitin vectors are not well described. I assume these are ubiquitin with only a single lysine at K27, K29 or K33. This should be better described in the figure legends (and Methods).
5) The statement "ITCH and other HECT-type E3 ligases reportedly synthesize K27, K29 or K33-linked ubiquitin chains on various substrates" (p.9 l.17). This rather general statement is incorrect/misleading. Different HECT-type E3 ligases have different chain type specificity. Also, on p. 17 l. 11, the authors write that ITCH also forms K63-linked ubiquitin chains on WWOX.
6) In many figures, information is missing how often these experiments were conducted and if the results were consistent between experiments. This needs to be added. Examples include figures 2B-F, 3B-G, 4C.
7) The error bars in Fig 1A are still not defined in the figure legend. The authors need to state whether these are SD or SEM or something else.
8) P. 15, l. 8/9: "Plasma membrane-localized ENTREP associates with ITCH and converts it from the intramolecularly closed, inactive form to the active form by binding to the WW domain." This statement is very speculative and there is no data in the manuscript that would indicate such a mechanism.
9) Despite the authors' claim, the Western Blotting is still not described in the Methods.
10) Typos:
a. p. 14, l 24: CXCL2 should read CXCL12

**Point-to-point reply to the Reviewers:**

Black letters are the reviewers’ comments; blue letters are our reply.

**Referee #1:**

The authors have performed extensive revisions. Unfortunately, however, not all points were addressed adequately. In my eyes, the manuscript would therefore need considerable additional work:

For example, the authors have performed a pull-down experiment under denaturing conditions (new Figure 2C), as I had requested. In contrast to their previous data, this experiment shows increased substrate ubiquitination in the presence of the K27/29/33 single-Lys-mutants of ubiquitin. However, it is unclear whether the observed higher-order bands are due to ubiquitin chain formation or multi-mono-ubiquitination. Furthermore, several features of the new experiment are odd:

- Why do the K27/29/33 mutants give rise to more substrate ubiquitination than the WT?
- Why do the K48- and K63-only mutants show a loss of mono-ubiquitination of the substrate, while the K0-mutant can still monoubiquitinate?

If these effects are real, they should be quantified based on at least three experimental replicates. Furthermore, seeing a stimulation of activity towards ubiquitin mutants in which 6 residues have been altered (K27-only, K29-only, K33-only) strongly corroborates my previous request that this experiment should be performed with single Lys-to-Arg

(K6R, K11R, K27R etc) mutants. In order to come to a meaningful conclusion, such experiments must be conducted. Based on the result, the authors should also interrogate the existence of chains (rather than multi-monoubiquitination on the substrate, e.g. using linkage-specific antibodies.

Right now, the text refers to "Lysine-27, 29 and 33-linked multi-monoubiquitin", which makes no sense.

I want to emphasize that the requested experiments are standard in the field (while even more advanced methods, such as quantitative MS are routineously employed), and the reagents readily available. I am therefore not satisfied with the statement "As for the experiments using K/R-mutants, which we thought crucial, we have not finished yet because we did not see the selectivity of ubiquitin linkage and might need a various K/R-mutants"

Sorting out the type of ubiquitin modification that is formed on ENTREP is also relevant for the interpretation of the consecutive experiments (e.g. the cycloheximide chase assay and effecs on ENTREP stability).

**Response-** Thank you very much for your comments. Our premature and confusing estimation in the previous manuscript was drawn from the poor results of nickel-pulldown assay: with our hands, the nickel-beads used for the assay did not yield much pulldowned protein. Unfortunately, we did not completely solve the problem with the beads, which might have bound ENTREP-myc/HIS very weakly or hardly released it in the elution buffer. Therefore, for this revision, we increased the volume of beads in the assay using HA-tagged wild type of ubiquitin (HA-ub wild), and this modification, though it produced a weak background, allowed three independent experiments to show consistent results: ITCH produced three bands of HA-ub wild-incorporating ENTREP (new Fig 2C in this revision).

We then repeated the nickel-pulldown assay with K-mutants of HA-tagged ubiquitin vectors and found that all types of K-mutants showed two or three HA-positive ENTREP bands, as attached below. Next, we constructed the expression vectors for HA-tagged K/R-mutants (K27R, K29R, K33R, K27+29R, K27+33R, K29+33R and K27+29+33R) and used them for the nickel-pulldown assay under the denaturing condition. As with K-mutants, K/R-mutants showed two or three HA-positive bands, as attached below.

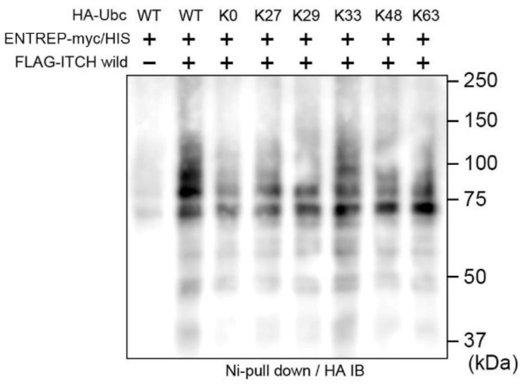


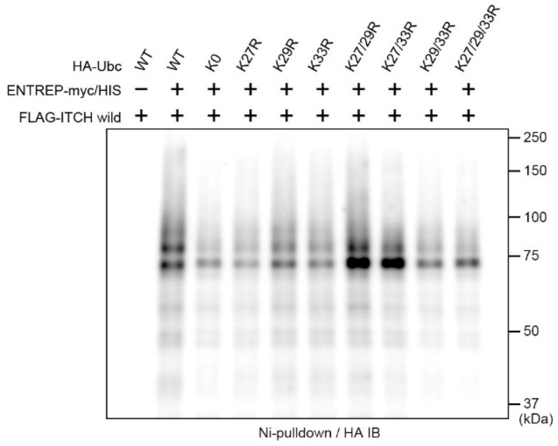


From these results, we assumed a multi-monoubiquitination of ENTREP. Once we assumed so, we were stuck for an answer why K27/29/33-mutants showed more intense bands than the wild did, as you asked regarding the previous manuscript. Under this circumstance, we decided to retract the data and estimation of K-mutant-using nickel-pulldown assay in the previous manuscript.

In order to conclude the ubiquitination of ENTREP, we asked Drs. Yasushi Saeki, Hikaru Tsuchiya and Takuya Tomita to determine the ENTREP ubiquitination by MS-based analysis of the ubiquitin absolute quantification/parallel reaction monitoring (AQUA/PRM) method (reference 1-3, listed below). The results by the method revealed that ITCH modifies ENTREP with a multi-monoubiquitin, and to a lesser extent, with K63-linked ubiquitin (new Fig 2D and Fig EV2E; new Appendix Table S2 for raw data of ubiquitin-AQUA/PRM). Furthermore, the shotgun MS analysis revealed ubiquitination of K274, K329 and K365 of ENTREP (new Appendix Fig S3). This shotgun MS analysis failed to cover all of lysine residues of ENTREP protein, but we think that its result supports that of ubiquitin-AQUA/PRM analysis.

We would like you to accept our revised conclusion of ENTREP ubiquitination, which was based on the MS-based analysis.

Reference 1: Tsuchiya H, Tanaka K, Saeki Y (2013) The parallel reaction monitoring method contributes to a highly sensitive polyubiquitin chain quantification. *Biochem Biophys Res Commun* 436: 223-229

Reference 2: Tsuchiya H, Ohtake F, Arai N, Kaiho A, Yasuda S, Tanaka K, Saeki Y (2017) In Vivo Ubiquitin Linkage-type Analysis Reveals that the Cdc48-Rad23/Dsk2 Axis Contributes to K48-Linked Chain Specificity of the Proteasome. *Mol Cell* 66: 488-502

Reference 3: Kaiho-Soma A, Akizuki Y, Igarashi K, Endo A, Shoda T, Kawase Y, Demizu Y, Naito M, Saeki Y, Tanaka K et al (2021) TRIP12 promotes small-molecule-induced degradation through K29/K48-branched ubiquitin chains. *Mol Cell* 81: 1411-1424

Figure 2D:
I am worried about the fact that the authors obtain a different result upon repeating their experiment than in the previous version of the manuscript. No explanation is provided for this, except "With your comments in mind, we carefully repeated the immunoprecipitation analyses using ITCH mutant. As shown in new Fig 2D, ITCH C830A did not obviously reduce the ENTREP co-precipitation of EPN1 and ENTREP mut1+2 mutant similarly co-precipitated in the presence of active ITCH. Based on these results, we modify our interpretation".
Under these circumstances, I can only reiterate my advice that the authors should repeat their experiment three times and report a quantification with error bars. This has also been requested by Reviewer 3 and should be done in all blots/IFs, based on which quantitative conclusions are drawn.
**Response-** Thank you very much for your comments. We repeated the immunoprecipitation analysis three times and their quantitation was included in new Fig 2E.

**Referee #2:**
During the revision of their manuscript now titled "ENTREP/FAM189A2 downregulated in breast cancer encodes a new activator for ITCH ubiquitin ligase to regulate ubiquitination and endocytosis of CXCR4", Tsunoda et al. addressed most, but not all, of our comments and suggestions. The authors added significant amounts of new data, and especially the pull-down experiments under denaturing conditions, the PLA experiments and addition of the catalytically-dead ITCH C830A mutant, significantly strengthen or refine some of the conclusions of the initial manuscript.
Based on the revised data, there is a strong case that ITCH interacts with ENTREP via an WW1-4/PPxY motif interaction and that ITCH directly ubiquitinates ENTREP. The interaction between ENTREP and ITCH furthermore increases ubiquitination of the ITCH substrate CXCR4. ENTREP also interacts with EPN1 in an ITCH/ubiquitin-independent manner and facilitates EPN1 and ITCH endocytosis and thus controls CXCR4 downstream signalling.
Some of the newly added data does not support their initial model, which they now

adjusted accordingly. In part because of these changes to the manuscript, parts of it are now quite difficult to follow and several open issues/concerns remain as detailed below.

1) Page 9 line 15: "EPN1 association of ENTREP was not solely dependent on its ubiquitination". What do the authors mean by this term? Is it dependent on ubiquitination or not? Based on the data it seems that ENTREP ubiquitination by ITCH has no effect on its interaction with EPN1. Thus, the only conclusion the authors can draw is that EPN1 association of ENTREP was not dependent on ITCH-mediated ubiquitination. Still in their final model figure, ENTREP ubiquitination by ITCH is prominently featured and it looks like this would recruit EPN1 as EPN1 is drawn in close proximity to one of the ubiquitin molecules. The model should be adjusted to reflect that ITCH-mediated ubiquitination on ENTREP does not affect EPN1 recruitment.

**Response-** Thank you very much for your comments. We rephrased in this revised manuscript as follow: "these evidences indicated that the association of ENTREP with EPN1 was not dependent on the ubiquitination of ENTREP" (page10, line 6-8 of new manuscript). We also adjusted Fig 5C, in which EPN1 does not attach to ubiquitin on ENTREP.

2) In the new manuscript the authors describe that ITCH forms "Lysine-27, 29 and 33-linked multi-monoubiquitin rather than polyubiquitin" on ENTREP. How do the authors derive this conclusion? And what do the authors mean by "Lysine-27, 29 and 33-linked multi-monoubiquitin"? A monoubiquitin on a substrate does not utilise any of the ubiquitin lysine residues. Lysine-27 etc. become only relevant in polyubiquitin chains (di-ubiquitin at the minimum). Fig 2C shows significantly less ENTREP ubiquitination with K0 ubiquitin and mostly mono-ubiquitination (top blot) suggesting that short poly-ubiquitin chains (mostly linked via K27, K29 or K33) are formed on ENTREP by ITCH. This needs to be clarified in the manuscript.

**Response-** Thank you very much for your comments. As mentioned above, for this revision we repeated the nickel-pulldown assay using increased volume of beads and HA-tagged wild type of ubiquitin (HA-ub wild) (new Fig 2C in this revision). And to conclude the ubiquitination of ENTREP, we employed MS-based ubiquitin-AQUA/PRM analysis (new Fig 2D and Fig EV2E; new Appendix Table S2) as well as shotgun MS analysis (new Appendix Fig S3), which is preliminary. Based on these results, we conclude that ITCH modifies ENTREP with a multi-monoubiquitin and, to a lesser extent, with K63-linked ubiquitin.

As we wrote above, we have not completely solved the problem with the beads,

which might have bound ENTREP-myc/HIS very weakly or hardly released it in the elution buffer. Therefore, we have decided to retract the data and estimation of K-mutant-using nickel-pulldown assay of the previous manuscript, in which we used a small volume of the nickel-beads. Instead, we would like you to accept the results of MS-based analysis showing the status of ITCH-mediated ubiquitination of ENTREP.

3) The authors should consider renaming their ENTREP KO cell line throughout the manuscript. MCF-7cripsr is not a good descriptor.
**Response-** We renamed it MCF-7-ko in this revision.

4) The K27, K29 and K33 ubiquitin vectors are not well described. I assume these are ubiquitin with only a single lysine at K27, K29 or K33. This should be better described in the figure legends (and Methods).
5) The statement "ITCH and other HECT-type E3 ligases reportedly synthesize K27, K29 or K33-linked ubiquitin chains on various substrates" (p.9 l.17). This rather general statement is incorrect/misleading. Different HECT-type E3 ligases have different chain type specificity. Also, on p. 17 l. 11, the authors write that ITCH also forms K63-linked ubiquitin chains on WWOX.
**Response-** As mentioned above, we would like you to accept that we do not use the data of nickel-pulldown assay using K-mutants. We deleted the statement in new manuscript.

6) In many figures, information is missing how often these experiments were conducted and if the results were consistent between experiments. This needs to be added. Examples include figures 2B-F, 3B-G, 4C.
7) The error bars in Fig 1A are still not defined in the figure legend. The authors need to state whether these are SD or SEM or something else.
**Response-** We fixed them in this manuscript.

8) P. 15, l. 8/9: "Plasma membrane-localized ENTREP associates with ITCH and converts it from the intramolecularly closed, inactive form to the active form by binding to the WW domain." This statement is very speculative and there is no data in the manuscript that would indicate such a mechanism.
**Response-** We modified the statement as follow: Plasma membrane-localized ENTREP associates with ITCH by binding to its WW domain. ITCH modifies ENTREP mainly through the attachment of multi-monoubiquitin. In addition, ENTREP enhances the ITCH

association and polyubiquitination of ligand-stimulated CXCR4, which leads to the attachment of endocytic adaptors such as AP-2 and CLASPs on CXCR4 (Reider & Wendland, 2011). (page15, line 15-19).

9) Despite the authors' claim, the Western Blotting is still not described in the Methods.
10) Typos:
a. p. 14, l 24: CXCL2 should read CXCL12
**Response-** Thank you for your comments. We fixed them in this revision.

Dear Dr. Kasai,

Thank you for submitting your revised manuscript. It has now been seen by two of the original referees.

As you can see, the referee finds that the study is significantly improved during revision and recommends publication. However, I need you to address the editorial points below before I can accept the manuscript.

• Please address the remaining minor concerns of referees and provide point-by-point response.
• We note that currently the manuscript is in Report format. However, character count if the manuscript is ~31000, which is too high for this format. Please either make sure that main text is 25,000 (+/- 2,000) characters, excluding references and materials and methods. Or, consider converting one of the EV figures into a main figure and separating Results and Discussion sections. (please see https://www.embopress.org/page/journal/14693178/authorguide#manuscriptpreparation for more information).
• The title is currently too long. Please propose a title with 100 characters or less (spaces included).
• We note that the data citations in the reference list are missing the [DATASET] label. In the Reference list, data citations must be labeled with "[DATASET]". A data reference must provide the database name, accession number/identifiers and a resolvable link to the landing page from which the data can be accessed at the end of the reference. Further instructions are available at <http://embor.embopress.org/authorguide#datacitation>.
• Our production/data editors have asked you to clarify several points in the figure legends (see attached document). Please incorporate these changes to the latest version of the manuscript file.

Thank you again for giving us to consider your manuscript for EMBO Reports, I look forward to your minor revision.

Kind regards,

Deniz Senyilmaz Tiebe

--
Deniz Senyilmaz Tiebe, PhD
Scientific Editor
EMBO Reports

Referee #1:

The use of quantitative mass spectrometry was useful to sort out the issues regarding chain specificity that were discussed in the last 2 rounds.

I have two additional comments:
- As far as I can see, the MS experiment (Figure 2D) does not show any errors even though the figure legend and the methods section specify that "The result was based on 3 biological replicates" (Figure Legend 2D) and "means and standard deviations were calculated from 3 biological replicates". These errors should be included.

- I am wondering why there is a lot more FLAG-Entrep signal in all the Halo-Itch-expressing samples compared to the Halo-only samples. While the authors comment on the fact that they loaded half the volume for the ITCH-expressing samples, they do not provide an explanation (as far as I can see). Is this a result of the co-transfection?

Referee #2:

The authors have included additional data in the revised manuscript and replaced some of the previous experiments using ubiquitin mutants with more sophisticated AQUA-MS analysis. While the new data, again, does not agree with some of the previous results, in my view, the AQUA-MS analysis is the superior technique and thus strengthens confidence in the data that now makes a strong point for ENTREP modification by multi-monoubiquitination and short K63-linked ubiquitin chains. However, a few technical details and interpretation of the AQUA-MS data need to be clarified.

1) Fig 2D: The authors distinguish between monoubiquitin/end cap ubiquitin and K63-linked di-ub, e.g., for sample #B, represents ENTREP with di-Ub represents a mixture of 2x mono-Ub and a single K63 Di-Ub. Since every K63 Di-Ub has a single end-cap ubiquitin, the measured fractions of 17.5% K63 Di-Ub and 79.3% monoubiquitin/end cap would suggest that ~35% of all ubiquitin molecules are present in K63 Di-Ub chains and ~65% in multi-mono Ub. This should be clarified in the text. The statement on p.9, l.15 "We detected K63-linked ubiquitin at 3.61% (#A), 17.84% (#B) and 16.86% (#C) of total ubiquitin." is

misleading.
2) I appreciate that the authors renamed their CRISPR knockout cell line. However, for me the concern was not about using CRISPR or KO, but to include the gene name that was knocked-out to avoid any confusion by reader. I again suggest using MCF-7 ENTREP-KO or MCF-7 ENTREP-/- or similar.

**Response to Editor and Reviewers**

Dear Editor of *EMBO reports*,

Thank you very much for your thoughtful comments and advises on the revised manuscript. And we appreciate another round of chance to improve our manuscript. Here we submit the manuscript, which is re-formatted as *Article*.

We would appreciate it if you find our manuscript be suitable for publication as *Article* of *EMBO reports*.

**Point-to-point reply to the Editor:**

Black letters are the Editor's comments and advises; blue letters are our reply.

Please address the remaining minor concerns of referees and provide point-by-point response.

**Response-** We appreciate the Reviewers' comments. We attach the point-to-point reply to Reviewers after the reply to the Editor.

We note that currently the manuscript is in Report format. However, character count if the manuscript is ~31000, which is too high for this format. Please either make sure that main text is 25,000 (+/- 2,000) characters, excluding references and materials and methods. Or, consider converting one of the EV figures into a main figure and separating Results and Discussion sections. (please see https://www.embopress.org/page/journal/14693178/authorguide#manuscriptpreparation for more information).

**Response-** We changed the format of our manuscript: this new manuscript has the Results section separated from Discussion. And we adjusted jammed five main figures and five EV figures of previous manuscript to eight main figures along with four EV figures in this new manuscript. We appreciate it if you find this new manuscript be suitable as an *Article* of *EMBO reports*.

The title is currently too long. Please propose a title with 100 characters or less (spaces included).

**Response-** We shortened the title.

We note that the data citations in the reference list are missing the [DATASET] label. In

the Reference list, data citations must be labeled with "[DATASET]". A data reference must provide the database name, accession number/identifiers and a resolvable link to the landing page from which the data can be accessed at the end of the reference. Further instructions are available at http://embor.embopress.org/authorguide#datacitation.

**Response-** We fixed the reference list. But unfortunately, we failed Endonote software to delete () marks of references in the main text. We appreciate it if your production editors help us to fix this.

Our production/data editors have asked you to clarify several points in the figure legends (see attached document). Please incorporate these changes to the latest version of the manuscript file.

**Response-** Thank you very much for your instruction. We incorporated those in this new manuscript.

**Overview of the Figures in this new manuscript:**

| | |
|---|---|
| Fig 1: same to previous Fig 1. | |
| Fig 2 contains | **A** (not changed) |
| | **B** (same to previous EV2A) |
| | **C** (same to previous 2B) |
| | **D** (same to previous EV2B) |
| | **E** (same to previous EV2C) |
| | **F** (same to previous EV2D) |
| Fig 3 contains | **A** (same to previous 2C) |
| | **B** (same to previous 2D, with new schematic presentation) |
| Fig 4 contains | **A** (same to previous 2E) |
| | **B** (same to previous EV3A) |
| | **C** (same to previous EV3B) |
| | **D** (same to previous 2F) |
| | **E** (same to previous 2G) |
| Fig 5 contains | **A-G** (all same to previous 3A-3G) |
| Fig 6 contains | **A-C** (all same to previous 4A-4C, with changes of MCF-7-ko) |
| Fig 7 contains | **A** (same to previous 5A, with change of MCF-7-ko) |
| | **B** (same to previous 5B, with changes of MCF-7-ko) |
| Fig 8 contains | schematic presentation, which is same to previous 5C. |

**Overview of the Expanded View Figures:**

Fig EV1: same to previous EV1.

Fig EV2: previous EV2A, EV2B, EV2C, EV2D moved to main figures.

Fig EV3: same to previous EV4.

Fig EV4: same to previous EV5.

**Overview of the Appendix Figures and Table in the revised manuscript:**

Appendix Fig S1-S4: same to previous S1-4, with a change of MCF-7-ko (S4).

Appendix Table S1-S2: same to previous Table S1-S2.

**Point-to-point reply to the Reviewers:**

Black letters are the reviewers' comments; blue letters are our reply.

**Referee #1:**

The use of quantitative mass spectrometry was useful to sort out the issues regarding chain specificity that were discussed in the last 2 rounds.

I have two additional comments:

- As far as I can see, the MS experiment (Figure 2D) does not show any errors even though the figure legend and the methods section specify that "The result was based on 3 biological replicates" (Figure Legend 2D) and "means and standard deviations were calculated from 3 biological replicates". These errors should be included.

**Response-** Considering the readers' convenience, we demonstrated the ratios of ubiquitin as Circular graphs (Fig 3B of new manuscript). And the raw data of all samples analyzed by the ubiquitin-AQUA/PRM is available in Appendix Table S2.

- I am wondering why there is a lot more FLAG-Entrep signal in all the Halo-Itch-expressing samples compared to the Halo-only samples. While the authors comment on the fact that they loaded half the volume for the ITCH-expressing samples, they do not provide an explanation (as far as I can see). Is this a result of the co-transfection?

**Response-** As we wrote in previous manuscript, we mentioned again in this new manuscript as follow: we found that co-transfection of ITCH slightly stabilized ENTREP protein in the cycloheximide chase assay (Fig EV2B), but a role of ENTREP ubiquitination is to be determined in future studies (page 9, line 23-25 of this new manuscript).

Referee #2:

The authors have included additional data in the revised manuscript and replaced some of the previous experiments using ubiquitin mutants with more sophisticated AQUA-MS analysis. While the new data, again, does not agree with some of the previous results, in my view, the AQUA-MS analysis is the superior technique and thus strengthens confidence in the data that now makes a strong point for ENTREP modification by multi-monoubiquitination and short K63-linked ubiquitin chains. However, a few technical details and interpretation of the AQUA-MS data need to be clarified.

1) Fig 2D: The authors distinguish between monoubiquitin/end cap ubiquitin and K63-linked di-ub, e.g., for sample #B, represents ENTREP with di-Ub represents a mixture of 2x mono-Ub and a single K63 Di-Ub. Since every K63 Di-Ub has a single end-cap ubiquitin, the measured fractions of 17.5% K63 Di-Ub and 79.3% monoubiquitin/end cap would suggest that ~35% of all ubiquitin molecules are present in K63 Di-Ub chains and ~65% in multi-mono Ub. This should be clarified in the text. The statement on p.9, l.15 "We detected K63-linked ubiquitin at 3.61% (#A), 17.84% (#B) and 16.86% (#C) of total ubiquitin." is misleading.

**Response-** We thank you very much for your comments. Comparing the band size of #A-C with non-ubiquitinated ENTREP, the samples from gel area corresponding to #A-C are expected to contain one, two and three Ubs, respectively. Therefore, for instance, #B contains a mixture of two types of ENTREP molecules: ENTREP harboring two of single-Ub (two endo caps) OR ENTREP harboring one di-Ubs (which contains one K63 and one endo cap). Now we know K63 of #B is 17.84% (= one sixth of 100%), so the former molecule should be two thirds and the latter molecule should be one third of #B. To avoid misinterpretation, we deleted the statement, instead, attach a schematic presentation in this new manuscript (Fig 3B) to explain how to evaluate the results of MS analysis.

2) I appreciate that the authors renamed their CRISPR knockout cell line. However, for me the concern was not about using CRISPR or KO, but to include the gene name that was knocked-out to avoid any confusion by reader. I again suggest using MCF-7 ENTREP-KO or MCF-7 ENTREP-/- or similar.

**Response-** We fix this in this new manuscript.

Dear Dr. Kasai,

Thank you for submitting your revised manuscript. I have now looked at everything and all is fine. Therefore, I am very pleased to accept your manuscript for publication in EMBO Reports.

Congratulations on a nice work!

Kind regards,

Deniz Senyilmaz Tiebe
--
Deniz Senyilmaz Tiebe, PhD
Editor
EMBO Reports

--

At the end of this email I include important information about how to proceed. Please ensure that you take the time to read the information and complete and return the necessary forms to allow us to publish your manuscript as quickly as possible.

As part of the EMBO publication's Transparent Editorial Process, EMBO reports publishes online a Review Process File to accompany accepted manuscripts. As you are aware, this File will be published in conjunction with your paper and will include the referee reports, your point-by-point response and all pertinent correspondence relating to the manuscript.

If you do NOT want this File to be published, please inform the editorial office within 2 days, if you have not done so already, otherwise the File will be published by default [contact: emboreports@embo.org]. If you do opt out, the Review Process File link will point to the following statement: "No Review Process File is available with this article, as the authors have chosen not to make the review process public in this case."

Should you be planning a Press Release on your article, please get in contact with emboreports@wiley.com as early as possible, in order to coordinate publication and release dates.

Thank you again for your contribution to EMBO reports and congratulations on a successful publication. Please consider us again in the future for your most exciting work.

Yours sincerely,

Deniz Senyilmaz Tiebe, PhD
Editor
EMBO Reports

*********************************************************************************

THINGS TO DO NOW:

You will receive proofs by e-mail approximately 2-3 weeks after all relevant files have been sent to our Production Office; you should return your corrections within 2 days of receiving the proofs.

Please inform us if there is likely to be any difficulty in reaching you at the above address at that time. Failure to meet our deadlines may result in a delay of publication, or publication without your corrections.

All further communications concerning your paper should quote reference number EMBOR-2020-51182V4 and be addressed to emboreports@wiley.com.

Should you be planning a Press Release on your article, please get in contact with emboreports@wiley.com as early as possible, in order to coordinate publication and release dates.

EMBO PRESS

YOU MUST COMPLETE ALL CELLS WITH A PINK BACKGROUND

PLEASE NOTE THAT THIS CHECKLIST WILL BE PUBLISHED ALONGSIDE YOUR PAPER

Corresponding Author Name: Kenji Kasai
Journal Submitted to: EMBO reports
Manuscript Number: EMBOR-2020-51182V1

USEFUL LINKS FOR COMPLETING THIS FORM

http://www.antibodypedia.com
http://1degreebio.org
http://www.equator-network.org/reporting-guidelines/improving-bioscience-research-repor
http://grants.nih.gov/grants/olaw/olaw.htm
http://www.mrc.ac.uk/Ourresearch/Ethicsresearchguidance/Useofanimals/index.htm
http://ClinicalTrials.gov
http://www.consort-statement.org
http://www.consort-statement.org/checklists/view/32-consort/66-title
http://www.equator-network.org/reporting-guidelines/reporting-recommendations-for-tumo
http://datadryad.org
http://figshare.com
http://www.ncbi.nlm.nih.gov/gap
http://www.ebi.ac.uk/ega
http://biomodels.net/
http://biomodels.net/miriam/
http://jjj.biochem.sun.ac.za
http://oba.od.nih.gov/biosecurity/biosecurity_documents.html
http://www.selectagents.gov/

## Reporting Checklist For Life Sciences Articles (Rev. June 2017)

This checklist is used to ensure good reporting standards and to improve the reproducibility of published results. These guidelines are consistent with the Principles and Guidelines for Reporting Preclinical Research issued by the NIH in 2014. Please follow the journal's authorship guidelines in preparing your manuscript.

### A- Figures
### 1. Data
**The data shown in figures should satisfy the following conditions:**

- the data were obtained and processed according to the field's best practice and are presented to reflect the results of the experiments in an accurate and unbiased manner.
- figure panels include only data points, measurements or observations that can be compared to each other in a scientifically meaningful way.
- graphs include clearly labeled error bars for independent experiments and sample sizes. Unless justified, error bars should not be shown for technical replicates.
- if n< 5, the individual data points from each experiment should be plotted and any statistical test employed should be justified
- Source Data should be included to report the data underlying graphs. Please follow the guidelines set out in the author ship guidelines on Data Presentation.

### 2. Captions
**Each figure caption should contain the following information, for each panel where they are relevant:**

- a specification of the experimental system investigated (eg cell line, species name).
- the assay(s) and method(s) used to carry out the reported observations and measurements
- an explicit mention of the biological and chemical entity(ies) that are being measured.
- an explicit mention of the biological and chemical entity(ies) that are altered/varied/perturbed in a controlled manner.
- the exact sample size (n) for each experimental group/condition, given as a number, not a range;
- a description of the sample collection allowing the reader to understand whether the samples represent technical or biological replicates (including how many animals, litters, cultures, etc.).
- a statement of how many times the experiment shown was independently replicated in the laboratory.
- definitions of statistical methods and measures:
  - common tests, such as t-test (please specify whether paired vs. unpaired), simple χ2 tests, Wilcoxon and Mann-Whitney tests, can be unambiguously identified by name only, but more complex techniques should be described in the methods section;
  - are tests one-sided or two-sided?
  - are there adjustments for multiple comparisons?
  - exact statistical test results, e.g., P values = x but not P values < x;
  - definition of 'center values' as median or average;
  - definition of error bars as s.d. or s.e.m.

Any descriptions too long for the figure legend should be included in the methods section and/or with the source data.

**In the pink boxes below, please ensure that the answers to the following questions are reported in the manuscript itself. Every question should be answered. If the question is not relevant to your research, please write NA (non applicable). We encourage you to include a specific subsection in the methods section for statistics, reagents, animal models and human subjects.**

### B- Statistics and general methods

Please fill out these boxes (Do not worry if you cannot see all your text once you press return)

| | |
|---|---|
| 1.a. How was the sample size chosen to ensure adequate power to detect a pre-specified effect size? | Sample size was chosen based on previous studies in the field. |
| 1.b. For animal studies, include a statement about sample size estimate even if no statistical methods were used. | NA |
| 2. Describe inclusion/exclusion criteria if samples or animals were excluded from the analysis. Were the criteria pre-established? | NA |
| 3. Were any steps taken to minimize the effects of subjective bias when allocating animals/samples to treatment (e.g. randomization procedure)? If yes, please describe. | NA |
| For animal studies, include a statement about randomization even if no randomization was used. | NA |
| 4.a. Were any steps taken to minimize the effects of subjective bias during group allocation or/and when assessing results (e.g. blinding of the investigator)? If yes please describe. | NA |
| 4.b. For animal studies, include a statement about blinding even if no blinding was done | NA |
| 5. For every figure, are statistical tests justified as appropriate? | Yes. |
| Do the data meet the assumptions of the tests (e.g., normal distribution)? Describe any methods used to assess it. | Yes. two-way ANOVA, Statistical analysis |
| Is there an estimate of variation within each group of data? | Yes. |

| | |
|---|---|
| Is the variance similar between the groups that are being statistically compared? | Yes. |

## C- Reagents

| | |
|---|---|
| 6. To show that antibodies were profiled for use in the system under study (assay and species), provide a citation, catalog number and/or clone number, supplementary information or reference to an antibody validation profile. e.g., Antibodypedia (see link list at top right), 1DegreeBio (see link list at top right). | All antibodies used in the study were from commercial sources. Vendor and catalog number for each antibody are in the Materials and Methods (page 21). |
| 7. Identify the source of cell lines and report if they were recently authenticated (e.g., by STR profiling) and tested for mycoplasma contamination. | The sources of cell lines are in the Materials and Methods (page 18). |

* for all hyperlinks, please see the table at the top right of the document

## D- Animal Models

| | |
|---|---|
| 8. Report species, strain, gender, age of animals and genetic modification status where applicable. Please detail housing and husbandry conditions and the source of animals. | NA |
| 9. For experiments involving live vertebrates, include a statement of compliance with ethical regulations and identify the committee(s) approving the experiments. | NA |
| 10. We recommend consulting the ARRIVE guidelines (see link list at top right) (PLoS Biol. 8(6), e1000412, 2010) to ensure that other relevant aspects of animal studies are adequately reported. See author guidelines, under 'Reporting Guidelines'. See also: NIH (see link list at top right) and MRC (see link list at top right) recommendations. Please confirm compliance. | NA |

## E- Human Subjects

| | |
|---|---|
| 11. Identify the committee(s) approving the study protocol. | NA |
| 12. Include a statement confirming that informed consent was obtained from all subjects and that the experiments conformed to the principles set out in the WMA Declaration of Helsinki and the Department of Health and Human Services Belmont Report. | NA |
| 13. For publication of patient photos, include a statement confirming that consent to publish was obtained. | NA |
| 14. Report any restrictions on the availability (and/or on the use) of human data or samples. | NA |
| 15. Report the clinical trial registration number (at ClinicalTrials.gov or equivalent), where applicable. | NA |
| 16. For phase II and III randomized controlled trials, please refer to the CONSORT flow diagram (see link list at top right) and submit the CONSORT checklist (see link list at top right) with your submission. See author guidelines, under 'Reporting Guidelines'. Please confirm you have submitted this list. | NA |
| 17. For tumor marker prognostic studies, we recommend that you follow the REMARK reporting guidelines (see link list at top right). See author guidelines, under 'Reporting Guidelines'. Please confirm you have followed these guidelines. | NA |

## F- Data Accessibility

| | |
|---|---|
| 18: Provide a "Data Availability" section at the end of the Materials & Methods, listing the accession codes for data generated in this study and deposited in a public database (e.g. RNA-Seq data: Gene Expression Omnibus GSE39462, Proteomics data: PRIDE PXD000208 etc.) Please refer to our author guidelines for 'Data Deposition'.<br><br>Data deposition in a public repository is mandatory for:<br>a. Protein, DNA and RNA sequences<br>b. Macromolecular structures<br>c. Crystallographic data for small molecules<br>d. Functional genomics data<br>e. Proteomics and molecular interactions | ENTREP cDNA sequence found in this study has been registered at the GenBank, the EMBL-EBI and the DDBJ under the accession number LC496047.1 (https://www.ncbi.nlm.nih.gov/nuccore/LC496047.1). |
| 19. Deposition is strongly recommended for any datasets that are central and integral to the study; please consider the journal's data policy. If no structured public repository exists for a given data type, we encourage the provision of datasets in the manuscript as a Supplementary Document (see author guidelines under 'Expanded View' or in unstructured repositories such as Dryad (see link list at top right) or Figshare (see link list at top right). | NA |
| 20. Access to human clinical and genomic datasets should be provided with as few restrictions as possible while respecting ethical obligations to the patients and relevant medical and legal issues. If practically possible and compatible with the individual consent agreement used in the study, such data should be deposited in one of the major public access-controlled repositories such as dbGAP (see link list at top right) or EGA (see link list at top right). | NA |
| 21. Computational models that are central and integral to a study should be shared without restrictions and provided in a machine-readable form. The relevant accession numbers or links should be provided. When possible, standardized format (SBML, CellML) should be used instead of scripts (e.g. MATLAB). Authors are strongly encouraged to follow the MIRIAM guidelines (see link list at top right) and deposit their model in a public database such as Biomodels (see link list at top right) or JWS Online (see link list at top right). If computer source code is provided with the paper, it should be deposited in a public repository or included in supplementary information. | NA |

## G- Dual use research of concern

| | |
|---|---|
| 22. Could your study fall under dual use research restrictions? Please check biosecurity documents (see link list at top right) and list of select agents and toxins (APHIS/CDC) (see link list at top right). According to our biosecurity guidelines, provide a statement only if it could. | NA |
